# The effectiveness of psychological interventions for fatigue in cancer survivors: systematic review of randomised controlled trials

**DOI:** 10.1186/s13643-019-1230-2

**Published:** 2019-12-13

**Authors:** T. K. Corbett, A. Groarke, D. Devane, E. Carr, J. C. Walsh, B. E. McGuire

**Affiliations:** 1 NIHR ARC Wessex, School of Health Sciences, University of Southampton, Highfield, Southampton, SO17 1BJ UK; 2 School of Psychology, National University of Ireland Galway, Galway, Ireland; 3 School of Nursing and Midwifery, National University of Ireland Galway, Galway, Ireland

**Keywords:** Cancer, Psychological, Survivorship, Fatigue, Post-treatment, Cancer-related fatigue, Psychooncology, Review, Narrative review

## Abstract

**Background:**

Fatigue is a common symptom in cancer patients that can persist beyond the curative treatment phase. This systematic review evaluated the effectiveness of psychological interventions for cancer-related fatigue in post-treatment cancer survivors.

**Methods:**

We searched relevant online databases and sources of grey literature. Randomised controlled trials (RCTs) evaluating psychological interventions in adult cancer patients after the completion of treatment, with fatigue as an outcome measure, were included. Two review authors extracted data independently from the selected studies and assessed the methodological quality using the Cochrane Collaboration Risk of Bias Tool.

**Results:**

Thirty-three psychological interventions were identified. The sample size of the included studies varied between 28 and 409, with 4525 participants overall. Twenty-three of the included studies reported a significant effect of the interventions on reducing fatigue in cancer survivors. Most interventions focused on psychoeducation, mindfulness, cognitive or behaviour therapy-oriented strategies. However, studies differed widely in terms of measurement tools used to assess fatigue, mode, duration and frequency of the intervention delivery.

**Conclusions:**

This review showed some tentative support for psychological interventions for fatigue after cancer treatment. However, as the RCTs were heterogeneous in nature and the number of high-quality studies was limited, definitive conclusions are not yet possible. With the growing need for stage-specific research in cancer, this review sought to inform current practice and to summarise the existing evidence base of randomised controlled trials in the area.

**Systematic review registration:**

PROSPERO registration number: CRD42014015219.

## Highlights

- The majority of treatments comprise standard components of CBT, mindfulness and/or psychoeducation. Studies comparing active psychological therapies are scarce. There is insufficient high-quality evidence to recommend psychological treatment as having possible benefit for cancer-related fatigue in post-treatment cancer survivors. There is no reported evidence of adverse effects.
- The majority of the evidence is for the treatment of fatigue in those with breast cancer but there is insufficient evidence to indicate if the treatments are more effective for one type of cancer over another.
- The interventions appear to have had some impact on mood, self-efficacy to cope with fatigue and quality of life/functional impact of fatigue. However, there appeared to be little impact of the interventions on pain. Interventions designed specifically for CrF did not tend to assess sleep variables.
- With wide-ranging heterogeneity in study design and measures used to assess the outcomes, it is difficult to evaluate which format or elements reduce fatigue after cancer treatment. Furthermore, the optimum time to intervene after treatment has ended is not clear.

## Background

Cancer-related fatigue (CrF) is commonly defined as ‘a distressing, persistent, subjective sense of physical, emotional and/or cognitive tiredness or exhaustion related to cancer and/or cancer treatment that is not proportional to recent activity, and significantly interferes with usual functioning’ [1]. There is little understanding of the underlying aetiology of CrF [2] but it is considered a multidimensional symptom that is comprised of physical, mental, and emotional aspects [1, 3, 4].

There is limited evidence of the effectiveness of pharmacological interventions for the management of CrF [5]. However, some reviews of non-pharmacological interventions have indicated that psychological and activity-based interventions may be effective [2, 6]. Interventions that incorporate restorative approaches, supportive-expressive techniques and cognitive-behavioural psychosocial interventions may reduce levels of CrF [6, 7]. In this review, we have focused on psychological therapies designed to improve functioning and/or reduce the physical and psychological impact of CrF.

Psychological interventions such as cognitive-behavioural therapy (CBT) aim to influence or change cognitions, emotions, behaviours or a combination of these [8]. Interventions which target these processes may improve symptom management in CrF [9]. These therapies may increase knowledge, improve emotional adjustment and enhance quality of life, and have also been associated with improved coping skills, physical health and functional adjustment [6, 10]. Patients and healthcare professionals have been reported to have high expectations of, and relatively positive attitudes towards, psychological therapies [10].

There is some evidence that psychosocial interventions are effective in reducing fatigue in patients undergoing active treatment for cancer [8]. While biological insults such as cancer or cancer treatment may lead to fatigue symptoms during the treatment phase of those with cancer, behavioural and cognitive variables may prolong fatigue during to post-treatment phase [1]. However, it is still unclear whether psychological interventions are helpful for managing fatigue in post-treatment cancer survivors beyond the early diagnostic and treatment phase [11]. Consequently, there is a need to conduct a critical review of the literature pertaining to psychological interventions in post-treatment cancer survivorship.

### Objectives

This review systematically reviews and synthesizes the evidence from randomised controlled trials (RCTs) investigating the effectiveness of psychological interventions for persistent fatigue in people after the completion of cancer treatment.

## Methods

The review protocol was registered with the International Prospective Register of Systematic Reviews (PROSPERO) database (registration number: CRD42014015219) and the protocol has been published [12]. The review is reported in accordance with the Preferred Reporting Items for Systematic Reviews and Meta-Analyses (PRISMA) statement [13].

### Criteria for considering studies for this review

#### Types of studies

RCTs comparing psychological treatments with no intervention (i.e. usual care or wait list controls), attention controls or another intervention for CrF. Studies were included regardless of treatment intensity or duration, mode of treatment delivery (e.g. individual, group) or medium of treatment (e.g. in-person, online). We did not impose date restrictions. Studies found in the grey literature were included if a full-text paper in English was available, either through databases or through contact with the study authors.

#### Types of participants

Adults 18 years and older who had completed treatment for cancer regardless of gender, tumour type, and type of medical treatment received.

#### Types of interventions

We included studies that evaluated the effect of psychological therapies in the management of CrF. Interventions including psychotherapy and psycho-education were included. These interventions included those that provided advice or information (verbal, written, audio-visual or computer delivered material) in order to help people understand and manage CrF, strategies such as cognitive restructuring, coping skill development, meditation or relaxation techniques. Studies that combined psycho-behavioural and non-psychological methods were included only if the study had a predominant emphasis on a psychological element in the design. Studies were excluded if they did not employ a psychotherapeutic rationale or theory in the intervention design [12].

#### Types of outcome measures

Studies were required to have ‘fatigue’ as an outcome of interest. In line with Goedendorp et al. [8], studies were included if fatigue was measured with a questionnaire designed specifically to evaluate fatigue. Fatigue subscales that were part of a broader quality-of-life measure were also included, if specific fatigue-related data were available. Fatigue could also be measured with a visual analogue scale (VAS) or as part of a symptom list and scored as ‘present’ or ‘absent’. Fatigue could be measured in terms of characteristics such as intensity, distress, duration, frequency, or as dimensions such as physical fatigue, mental fatigue or general fatigue.

#### Secondary outcomes included

- Functional impact of fatigue (self-report questionnaires measures assessing the impact of fatigue on daily functioning)
- Fatigue self-efficacy (self-reported scales of control or self-efficacy in relation to fatigue)
- Mood (self-reported scales of depression, and/or anxiety, or distress)
- Global quality of life (self-report questionnaires measures assessing the impact of fatigue on quality of life).

#### Information sources

The following electronic databases were searched: Cochrane Central Register of Controlled Trials (CENTRAL), MEDLINE, EMBASE, CINAHL PsycINFO, Web of Science and CancerLit. Alterations were made to the search strategies as appropriate for each database. An example search strategy can be seen in Table 1 (See Additional file 3. For further details of the search strategies used). The original search was conducted on October 6th and 7th 2015 and was updated on the 22nd and 23rd of January 2018. Studies from 2014 to 2018 were assessed for inclusion based on the criteria followed in the original search.

**Table 1:** Sample search strategy: details of the terms searched in CINAHL database

	Search term
1	‘cancer survivors’ OR ‘neoplasm’/exp OR neoplasm OR surviv* OR ‘cancer’/exp OR cancer OR ‘remission’/exp OR remission OR ‘post treatment’
2	psychology OR psych*or AND behaviour AND therapy OR hypnosis OR relaxation OR imagery OR cognition OR psychotherapy OR cognit*
3	fatigue OR asthenic OR asthenia OR exhaustion OR exhausted OR ‘loss of energy’ OR ‘loss of vitality’ OR weary OR weariness OR weakness OR apathy OR apathetic OR lassitude OR lethargic OR lethargy OR sleepy OR sleepiness OR drowsy OR drowsiness OR tired OR tiredness
4	‘randomized controlled trial’ OR controlled OR clinical OR trial OR ‘random assignment’
5	1 AND 2 AND 3 AND 4

Unpublished and ongoing trials were identified by checking appropriate databases of current ongoing clinical research studies. Grey literature was searched using the OpenGrey database (www.opengrey.eu), which includes technical or research reports or doctoral dissertations. Conference papers from annual American Society of Clinical Oncology (ASCO) or International Psycho Oncology Society World Congress (IPOS) conferences were also searched. Other published, unpublished and ongoing trials were identified by checking trials and protocols published on the following clinical trials registers and websites.

• World Health Organization International Clinical Trials Registry Platform (WHO ICTRP; www.who.int/ictrp/en).

• metaRegister of Controlled Trials (mRCT; www.controlled-trials.com/mrct/).

• ClinicalTrials.gov (www.clinicaltrials.gov).

• www.cancer.gov/clinicaltrials.

Search methods for identification of studies

### Data collection and analysis

One review author (TC) conducted the initial search before screening titles. Titles that were clearly not relevant to this review were removed. Three review authors (TC, EC and BMG) independently screened the remaining titles and abstracts for their eligibility for inclusion. Ineligible studies were excluded at this stage, with each author recording the reason for rejection. Full-text copies were retrieved and screened if the title and abstract did not provide sufficient information concerning the inclusion criteria for this review. Copies of all studies that possibly or definitely met the inclusion criteria were also retrieved. Disagreements between the reviewers were resolved by discussion, with the involvement of another reviewer where agreement could not be reached (DD). Multiple reports of the same study were included as a single study, with each study identified by the lead author of the primary results paper.

### Data extraction and management

Review authors (TC, EC, AG and BMG) extracted data independently from the studies using a specifically designed data extraction form (see Table 2). Authors were contacted where further clarity regarding the study was required, or in order to obtain additional data.

**Table 2:** Details of the interventions included in the review

Study	Content	Strategies	Time since treatment	Mode	Duration	Delivered by	Control group
Bantum 2014 [14]	Multiple health behaviour change program.	Skills building; information; encouragement; action planning; building self-efficacy; improving diet; increasing exercise; stress management via relaxation training;· processing and communicating emotional experiences; fatigue management	Had completed primary treatment within last 5 years	Online	6 × weeks	Cancer survivors mentored by the principal investigators.	Waitlist control
Bennett 2007 [15]	Motivational interviewing	Careful listening; summarising; feedback; barrier identification; affirmation; building self-efficacy	Had completed primary treatment at least 6 months prior to the study	In-person/Telephone	3 × 10-min MI sessions. 20-min per phone call	Physical activity counsellor and master’s-prepared research assistant	Usual care
Blaes 2016 [16]	Mindfulness based cancer recovery programme was used.	A range of Mindfulness meditation techniques practiced during group sessions , Expected to practice home meditation for 45 minutes a day, keep a log of home practice sessions along with doing mindfulness reading and reflective exercises	Had completed primary treatment at least 6 months prior to the study	Group	8 weekly 2.5 h classes and a full day silent retreat	University Faculty trained and certified in MBCR programme	Waitlist control
Bower 2015 [17]	Mindfulness	Information; mindfulness; relaxation; meditation; gentle movement exercises (e.g. mindful walking); psychoeducation; problem solving; working with difficult thoughts and emotions; managing pain; cultivation of loving kindness.	Had completed primary treatment at least 3 months prior to the study	Group	6 weekly × 2-h sessions. Daily home-practice 5–20 min		Waitlist control
Bruggeman-Everts 2017 [18]	Two different Web-based interventions aimed at reducing CCRF: (1) Ambulant Activity Feedback (AAF), and (2) Web-based Mindfulness-Based Cognitive Therapy (eMBCT)	AAF: involves taking notice of the Personal Digital Assistant messages, responding to these messages by changing physical activity, reading the weekly feedback from the physiotherapist, reporting experiences, and replying to the feedback by email. eMBCT: reading the weekly information, doing mindfulness exercises while listening to the MP3 files, filling out logs with their experiences, reading the weekly feedback of the therapist, and replying to this feedback by email weekly	Had completed primary treatment at least 3 months prior to the study	Online	3/hours per week, 9 weeks	AAF : physiotherapist & eMBCT: psychologist	Compared two different guided Web-based interventions compared to an unguided active control condition receiving psycho-educational emails
Carlson 2016 [19]	Mindfulness -based cancer recovery programme ( MBCR) VS Supportive expressive group therapy	Both based on existing available programmes. Mindfulness conscious awareness cultivated through training in mindfulness meditation and gentle yoga practices. SET facliitated mutual support, enhancing emotional expresiveness and coping, detoxifying feelings around death	Had completed primary treatment at least 3 months prior to the study	Group	8 weekly sessions of 90 min each plus a 6 h workshop (total of 18 h)	Research Assistants	Compared two empirically supported group interventions: mindfulness-based cancer recovery (MBCR) and supportive-expressive group therapy (SET). These were also compared to a minimal-treatment control condition that was a 1-day didactic stress management seminar.
Dirksen 2008 [20]	CBT- insomnia	Stimulus control instructions;· sleep restriction therapy; sleep education and hygiene; cognitive strategies; sleep diaries; discussing progress.	Had completed primary treatment at least 3 months prior to the study	Group	2-weeks pre-treatment6-weeks × treatment : 4 × week classes (1–2 h) and 2 × week telephone (15 mins) 2-weeks post-treatment	Master’s level Registered Nurse therapist	Education
Dodds 2015 [21]	Cognitively-based compassion training	CBCT was delivered in eight weekly, 2-h classes through didactics, class discussion, and guided meditation practice. Participants were asked to meditate at least three times per week using audio recordings of guided meditations (average length 30 min), and to maintain a practice log.	Treated with adjuvant systemic chemotherapy within the past 10 years	Group and individual	8 weekly 2 h classes and home meditation 3 times a week	The interventionist was a clinically trainedPh.D. social work researcher and experienced 20-year meditator fulfilling requirements for CBCT teacher certification of the Emory University-Tibet Science Initiative (ETSI).	Waitlist control
Dolbeault 2009 [22]	Psycho-educational group based on CBT	Self-monitoring; problem-solving; cognitive restructuring; communicate; relaxation.	Had completed primary treatment at least two weeks prior to the study (within the last year)	Group	8 weekly × 2-h sessions,	Led by 2 therapists, either psychologists or psychiatrists trained in group therapy and BCT	Waitlist control
Espie 2008 [23]	CBT- insomnia	Stimulus control; sleep restriction; cognitive therapy strategies.	Had completed primary treatment at least four weeks (1 month) prior to the study	Group	5 weekly, 50-min sessions	Cancer nurses, mentored by clinical psychologist	Usual care
Ferguson 2016 [24]	CBT-MAAT: cognitive behavioral therapy, Memory and Attention Adaptation Training	The 4 MAAT components include: 1) education, 2) self-awareness training to identify, 3) stress management and self-regulation, 4) cognitive compensatory strategies training	Had completed primary treatment at least 6 months prior to the study	Videoconference device	8 visits of 30 to 45 min	clinical psychologist	Compared cognitive behavioural therapy (CBT) Memory and Attention Adaptation Training (MAAT), with an attention control condition.
Fillion 2008 [25]	Psycho-education and physical activity	Relaxation skills; coping strategies; links between thoughts, emotions, and fatigue; self-regulation techniques (e.g. self-recording and goal setting); decrease passive coping strategies (e.g. behavioural and social disengagement and naps); increase awareness of the benefits of exercise; adherence techniques; reinforcement self-efficacy, motivation, and positive outcomes.	Completed their initial cancer treatment no longer than 2 years before enrolment	Group	4 weekly group meetings of 2.5-h and 1 × short telephone booster session (5–15 min)	Kinesiologist, trained research nurses,	Usual care
Foster 2016 [26]	Self-efficacy to manage CrF	Defines CRF (possible causes and effects); goal setting and planning; diet, sleep, exercise, home life and work; thoughts and feelings; strategies for talking to others; patient stories; self-monitoring; feedback; automated weekly emails; reminders.	Any time point following primary cancer treatment (within last 5 years)	Online	6 weeks	online	Waitlist control
Freeman 2015 [27]	Imagery-based intervention	Education on the mind–body connection; impact of mental imagery and the sensate experience on physiological processes; apply learning and receive peer-feedback; identify maladaptive ‘passive imagery’ (e.g. automatic thoughts focused on fear/loss of control); create adaptive ‘active imagery’ (e.g. thoughts focused on empowering, meaning–making themes); practice ‘targeted imagery’; monitor the effects of imagery on mind–body health.	At least 6 weeks after completing cancer treatment	Group/ tele-medicine	5 weekly 4-h group sessions (live delivery or telemedicine delivery). First 4 sessions separated into 3 modules (25-min didactic education; 25-min of group interaction; 20–30 min guided imagery). Brief (< 10 min) weekly phone calls during intervention delivery and for 3 × months post-treatment.	Licensed professional counsellor, and a family medicine physician	Compared live and telemedicine deliveries of an imagery-based behavioural intervention. Also had a waitlist control condition.
Gielissen 2006 [28]	CBT	Focused on six perpetuating factors (six modules) of post-cancer fatigue, which were based on existing literature and experience in clinical practice:Coping with the experience of cancer; fear of disease recurrence; dysfunctional cognitions concerning fatigue; dysregulation of sleep and activity; focus on low social support and negative social interactions.	Had completed primary treatment at least 1 year prior to the study	Individual	Number of sessions was determined by the number of modules used and whether the goal of the therapy was reached. 5–26 × 1-h therapy sessions over 6-month period (*M* = 12.5 sessions; SD = 4.7 sessions).	3x therapists with previous CBT experience with patients with chronic fatigue	Waitlist control
Heckler 2016 [29]	CBT- insomnia	Sleep hygiene guidelines Study medication instructed to take the study medication (armodafinil or placebo) in a split dose (7–9 am and 12–2 pm) for a total of 47 days	Had completed primary treatment at least four weeks (1 month) prior to the study	Individual	7 weeks; CBT-I sessions 1, 2, and 4 were in person (30–60 min in duration), and sessions 3, 5, 6, and 7 (15–30 min in duration) were by phone		Compared CBT-I to a wakefulness-promoting agent, armodafinil
Hoffman 2012 [30]	Mindfulness for CRF	Body scan; sitting/ walking/ compassion meditation; gentle hatha yoga; psycho-education related to CrF; class discussion; bedtime body scan; information (relationship of stress and fatigue, influence of the perception of exhaustion on subsequent diminished physical activity and that physical activity is helpful with CrF); mindful communication practice.	Had completed primary treatment at least 2 months prior to the study (completed their initial cancer treatment no longer than 2 years before enrolment)	Group	7 weeks × 2-h classes; Guided home practices (20 min)	MBSR teaching experience	Waitlist control
Johns 2015 [49]	MBSR-CRF	Body scan, sitting meditation, gentle hatha yoga, walking meditation, and compassion meditation; protocol was adapted for the cancer context, a practice that has precedent in previous studies ; MBSR-CRF adaptations included 2-h classes, seven classes instead of eight, no retreat, brief psycho-education related to CRF, and shorter guided home practices (20 min) to accommodate fatigued participants; however, all of the core content of the standard MBSR curriculum was included. Recordings of guided meditations of body scan, sitting meditation, gentle hatha yoga with chair adaptations, and compassion meditation were created by the facilitator for home practice.	Had completed primary treatment at least 3 months prior to the study	group	7 x 2-h classes; guided home practices (20 min)	instructor had 6 years of MBSR teaching experience, completing all components of professional training leading to eligibility for MBSR Teacher Certification Review (phase 4, Oasis Institute at the Center for Mindfulness in Medicine, Health Care and Society	Waitlist control
Lengacher 2012 [31]	Mindfulness	Awareness of thoughts and feelings through meditation practice (sitting and walking meditation, body scan, and gentle hatha yoga); informal mindfulness meditation; educational material related to relaxation, meditation, and the mind–body connection; pay attention and observe responses during stressful situations; group support sessions on emotional/ psychological responses and physical symptoms; discussion of barriers to the practice of meditation and application of mindfulness in daily situations; supportive interaction between group members.	Had completed primary treatment within 18 months prior to study	Group	6 weekly, 2-h sessions; Formal exercises (15–45 min per day, 6 × days per week; increased per week); Informal home practice; 1× day × 8-h silent retreat.	Licensed clinical psychologist trained in MBSR	Usual care
Matthews 2014 [32]	CBT- insomnia	Treatment rationale; conceptual model of insomnia; sleep restriction; stimulus control; sleep schedule; sleep hygiene; cognitive therapy: altering dysfunctional beliefs about sleep and the impact of sleep loss on daytime functioning; sleep titration and treatment gains; relapse prevention and skills to cope with setbacks.	Had completed primary treatment at least four weeks (1 month) prior to the study	Group/ individual 3 × sessions in person 2× sessions via telephone.	5 weekly sessions: Session 1: 60 mins; Session 2, 3 and 6: 30–45 min; Session 4 and 5 (Telephone): 15–20 min.	An advanced practice nurse with specialized training in CBTI	Active behavioural placebo treatment (BPT).
Prinsen 2013 [33]	CBT for post-cancer fatigue.	Information on coping with the experience of cancer; fear of disease recurrence; dysfunctional cognitions concerning fatigue; dysregulation of sleep; dysregulation of activity; discussion of low social support and negative social interactions; tailored physical activity program of walking or cycling; gradually replace physical activities by other activities.	Had completed primary treatment at least 1 year prior to the study	Group	12–14 (50 min) individual sessions in 6 months. Two daily sessions of tailored physical activity program	Psychologists	Waitlist control
Reeves 2017 [34]	Combined approach of increasing physical activity, reducing energy intake and behavioral therapy,	Received a detailed workbook, self-monitoring diary, digital scales, pedometer, calorie-counter book and up to 16 telephone calls over the intervention	Any time point following primary cancer treatment	Telephone-delivered	6 months: Telephone calls (weekly for 6 weeks followed by 10 fortnightly calls)	Lifestyle coaches, who were accredited practicing dietitians trained in exercise promotion and motivational interviewing	Usual care
Reich 2017 [35]	MBSR (BC)	1) Educational material related to relaxation, meditation, the mind-body connection, and a healthy lifestyle for survivors, 2) practice of meditation in group meetings and homework assignments, and 3) group processes related to barriers to the practice of meditation and supportive group interaction. training in formal meditation techniques (sitting meditation, body scan, gentle Hatha yoga, and walking meditation), along with informal techniques of integrating mindfulness into daily life activities. BCS were requested to formally and informally practice the meditative techniques for 15–45 minutes per day and to record their practice times in a daily diary. A manual and compact discs were provided to guide home practice.	Had completed primary treatment within previous 2 weeks (completed their initial cancer treatment no longer than 2 years before enrolment)	group	Six-week, 2-h per week sessions; practice the meditative techniques for 15–45 min per day	Psychologist trained in MBSR; Intervention sessions conducted by a single instructor were monitored weekly by a research assistant, who recorded time and delivery of the components of the two-hour class sessions on a fidelity checklist.	Waitlist control
Reif 2013 [47]	Patient education program	Problem solving; goal setting and evaluation; other cognitive techniques; behaviour therapy-oriented strategies and techniques; diary-keeping; perform exercises and implement lifestyle changes.	Any time point following primary cancer treatment	Group	6 weekly 90-min sessions. 2 × additional meetings after 3 and 6 months.	Nurses/ psychologist	Waitlist control
Ritterband 2012 [36]	CBT- insomnia	Introduction and rationale; sleep restriction; stimulus control; sleep hygiene; identify and restructure unhelpful beliefs about sleep; relapse prevention; high degree of individual tailoring and feedback; interactive elements; automated emails; encourage adherence.	Had completed primary treatment at least four weeks (1 month) prior to the study	Online	Access to Shuti for 9 weeks (6 week programme). Each core: 45 and 60 min.	NA	Waitlist control
Rogers 2017 [37]	Physical activity behaviour change intervention	Self-efficacy; outcome expectations; behavioural capability; observational learning; self-control; social support; personal behavioural modification plan; overcoming exercise barriers; emotional coping (including stress management); exercise benefits; task self-efficacy by gradual advancement of the exercise prescription; self-monitoring with daily activity log; overcoming exercise barriers experienced by the participant; self-monitoring; use of the behavioural modification plan; providing positive reinforcement; setting up for maintenance	Had completed primary treatment at least 2 months prior to the study	Group/individual	12-week programme: 6 group sessions during the first 8 weeks; 12 individual exercise sessions during the first 6 weeks; 3 individual counselling sessions during the final 6 weeks.	trained facilitators Psychologist/ exercise specialist	Provided publically available, printed materials
Sandler 2017 [38]	CBT and GET (Graded exercise) or education	Activity pacing, graded exercise, psychoeducation, sleep wake management, cognitive retraining, 3 optional CBT modules = coping , depression and anxiety management	Had completed primary treatment at least 3 months prior to the study	individual	12 weeks 5 45 min sessions with exercise therapist and 6 to 8 × 55 min sessions with psychologist conduced fortnightly	Clinical Psychologist and Exercise Physiologist	Education
Savard 2005 [39]	CBT- insomnia	Stimulus control therapy; sleep restriction; cognitive restructuring; sleep hygiene; fatigue and stress management	Had completed primary treatment at least four weeks (1 month) prior to the study	Group	8 weekly sessions of approximately 90 min	Master-level psychologist.	Waitlist control
Van Der Lee 2012 [40]	MBCT	Skills that enhance the ability to raise awareness to present experiences; information and instructions about various themes; home practice (CDs with breathing instruction and awareness exercises).	Had completed primary treatment at least 1 year prior to the study	Group	9-week group therapy, weekly sessions (2.5 h); 1 × 6 h session; 1 × 2.5 h follow-up session 2 × months after the 9th session. Total duration = 28.5 h.	Both therapists had followed MBSR training with Kabat Zinn.	Waitlist control
Van Weert 2010 [41]	CBT and physical activity	Self-management, goal setting, monitoring; norms and decision making, action, self-reflection; self-efficacy: mastery of experiences and perceived success, modelling, social persuasion, physiological feedback; discussion of irrational illness perceptions; finding effective and adaptive solutions to stressful problems; dysfunctional cognition, emotions, and behaviours; discussing distress, exercise physiology, and relaxation; homework assignment, and relaxation exercises; individual fitness goal- aerobic training muscle strength training, and information; information on the benefits of exercise; illustrative ‘model of fatigue,’; restore the balance between demand and capacity during tasks and activities.	Had completed primary treatment at least 3 months prior to the study	Group	1 h twice a week for 12 weeks (24 h individual physical training and 24 × hours of group sports and games). 24 h CBT (once a week, 2 × hours per session).	2 × physical therapists experienced in the delivery of physical training interventions to patients with cancer. CBT was supervised by 2 × psychologists.	Compared physical training combined with cognitive behavioural therapy with physical training alone and with no intervention.
Willems 2017 [51]	Psychosocial and lifestyle support	Self-management training; return-to-work; fatigue; anxiety and depression; social relationship and intimacy issues; physical activity, diet, smoking cessation; general information on the most common residual symptoms	Had completed primary treatment at least 4 weeks (1 month) prior to the study (within the last year)	Online	6 months	Stand-alone online	Waitlist control
Yun 2017 [42]	Health coaching	Physical activity, dietary habits, and distress management: individual tele-coaching: a TTM-based health education booklet and workbook for cancer survivors, 2) a workshop for empowerment of patients’ leadership skills, and 3) TTM-based telephone coaching with a health coaching manual (repeated assessment of stage of change, and planning how to achieve target health levels in accordance with their preferences and abilities)	Completed their initial cancer treatment no longer than 2 years before enrolment	Group/individual tele-coaching	1-h health education workshop 3-h leadership workshop individual coaching by telephone for a 24-week period (intervention only)- 16 sessions of tele-coaching were conducted: 30 min per week for 12 sessions, 30 min per 2 weeks for 2 sessions, and 30 min per month for 2 sessions were offered for the intervention group.	Health partners: long-term cancer survivors who formed partnerships with cancer patients and helped them achieve the target levels set for their health behaviors. Health master coaches: health professionals who mentored and supervised health partners.	Usual care
Yun 2012 [43]	CBT	Based on 2008 National Comprehensive Cancer Network & on the transtheoretical model (TTM) of health behaviour change and social cognitive theory as developed by Bandura or on cognitive behavioural therapy (CBT).Personally tailored sections based on the TTM model; physical activity; sleep hygiene; pain control; general introduction; energy conservation; nutrition; distress management; self-assessment and graphic reports; health advice; online education, caregiver monitoring and support; health professional monitoring.	Completed their initial cancer treatment no longer than 2 years before enrolment	Online	12 weeks	Independent research coordinator (nurse)	Usual care

### Assessment of risk of bias in included studies

The risk of bias of each trial was assessed as high risk, low risk or unclear risk as per recommendations provided in Chapter 8 of the Cochrane Hand book for Systematic Reviews of Interventions [44]. Further details regarding the risk of bias domains was provided in the study protocol [12].

### Quality of the evidence

The Grading of Recommendations Assessment, Development, and Evaluation (GRADE) process was used to assess the evidence for the primary comparison of ‘Psychological Interventions compared to usual care for Fatigue in cancer survivors’.

## Results

**Fig. 1:**
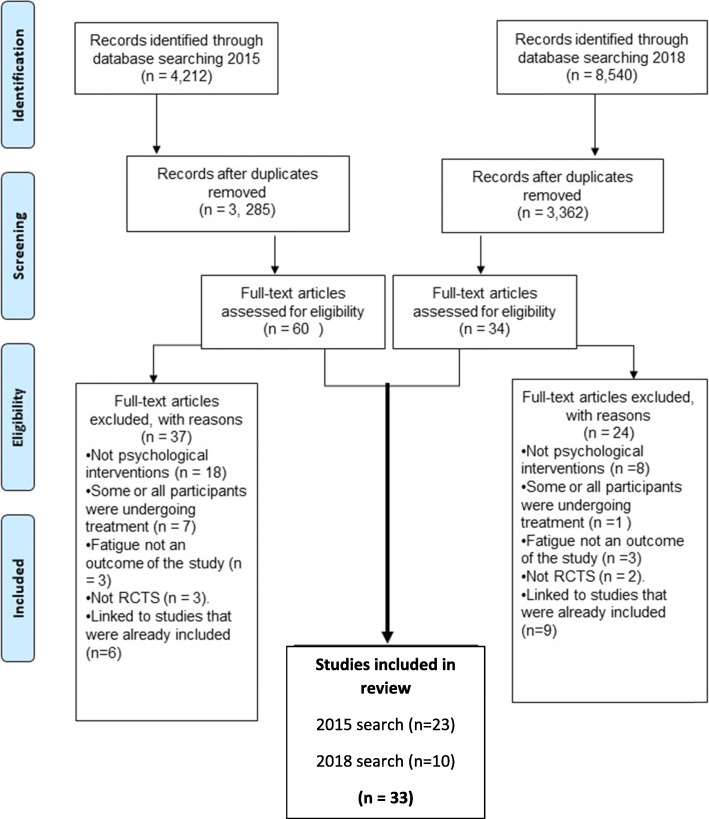
the PRISMA flow diagram of studies identified and excluded at each stage of the review

Figure 1 depicts the PRISMA flow diagram of studies identified and excluded at each stage of the review. The initial literature search of seven databases in 2015 resulted in 4212 potentially relevant articles. Following exclusion of duplicates, 3,285 articles remained. The titles and abstracts of these articles were screened and 60 full-text articles were selected to be retrieved and reviewed in detail. Following review of the full-text papers, a further 37 studies were excluded and 23 RCTs fulfilled all eligibility criteria for inclusion.

The updated search in 2018 resulted in 8540 potentially relevant articles. Once duplicates and studies prior to 2014 were removed, 3362 studies published were assessed for inclusion. Thirty-four full-text articles were reviewed, eight of which had already been included or were follow-up studies associated with papers included in the original review. Ten new papers were added to the review.

In total, 33 RCTs fulfilled all eligibility criteria for inclusion. A full description of these studies can be seen in Tables 2 and 3.

**Table 3:** Summary of findings for the main comparisons

Study	Measure used to assess fatigue	Total	*n* intervention	*n* Control	Final follow-up	Finding
Bantum 2014 [14]	Brief Fatigue Inventory (BFI)	303	156	147	6 months	*p* = 0.56 Effect size = 0.17 (Calculated by taking the differences of the means at 6 months predicted from the model, including adjustment factors, divided by the standard deviation for the difference computed from the within and between subject variance components.) Control group, • Baseline (*n* = 176); mean (95% CI) = 40.8 (38.9–42.8) • Month 6 (*n* = 156); mean (95% CI) = 40.7 (38.7–42.8) Intervention group • Baseline (*n* = 176); mean (95% CI) = 39.0 (37.0–40.9) • Month 6 (*n* = 147); mean (95% CI) = 36.4 (34.2–38.5)
Bennett 2007 [15]	Schwartz Cancer Fatigue Scale	56	28	28	6 months	On average, the level of fatigue status for all participants was 15.20 at baseline and declined 4.22 points (27%) across the study. Group × Time interaction for fatigue was significant [Λ =0.78, *F*(2,37) = 5.24, *p* = 0.010]. However, inspection of the graph showed this was an artifact of 3-month measures, whereas values at baseline and at 6 months showed no significant differences between groups, leading to the conclusion that the significant effect of the interaction was the result of measurement error.
Blaes 2016 [16]	Functional Assessment in cancer Therapy-Fatigue ( FACT-F)	42	28	14	4 months	There was an improvement in fatigue in both groups with time. Mean improvement from baseline to 4 months was 6.8 for the MBCR group and 1.3 for controls (*p* = 0.19). There was no statistically significant difference in improvement in fatigue for two groups.
Bower 2015 [17]	Fatigue Symptom Inventory	71	39	32	3 months	Mindfulness led to significant improvements in fatigue (*p* = 0.007), from pre- to post-intervention. No group differences in change from baseline to 3-month follow-up *p* = 0.57
Bruggeman-Everts 2017 [18]	Checklist Individual Strength - Fatigue Severity [CIS-FS] subscale	167	55	112	9 weeks	AAF = eMBCT = psycho-educationχ2(4)=27.63, *p* < .001 AAF = psycho-educationχ2(2)=28.28, *p* < .001 eMBCT = psycho-educationχ2(2)=10.89, *p* = .004 AAF = eMBCTχ2(2)=2.19, *p* = .34 Multiple group latent growth curve analysis, corrected for individual time between assessments, showed that fatigue severity decreased significantly more in the AAF and eMBCT groups compared to the psychoeducational group.
Carlson 2013 (2016) [19, 45]	POMS	271	113	158	6 and 12 months later.	Group-by-time effect at intervention (6months): *p* = 0.001 95% CI − 0.45 [− 0.70; − 0.20] Group-by-time effect at follow-up (12 months) *p* = 0.76
Dirksen 2008 [20]	Profile of Mood States Fatigue/Inertia Subscale (POMSF/I)	72	34	38	2 weeks	Statistically significant pre- to post-treatment change (*p* < 0.05). From pre- to post-treatment, the CBT-I group improved on fatigue. Statistically significant interaction effects were found for fatigue At post-treatment, a trend was noted towards lower fatigue [*t*(70) = 1·87, *p* = 0·07].
Dodds 2015 [21]	Medical Outcomes Study Short Form 12-Item HealthSurvey (SF-12)	28	16	12	4 weeks	Improvement in fatigue/vitality From baseline to study week 8 = 5.5, 95% CI [1.5; 9.6]; 1-month FU 0.3 95% CI [−4.2; 4.9] no significant differences at the 4- week follow-up.
Dolbeault 2009 [22]	POMSF/I and EORTC Fatigue	167	81	86	6 months	Comparison of change scores between randomisation arms (Group: n=81; Control: n=87) POMS fatigue • Group: E1 Mean (SD) 10.01 (7.38) ; E3 Mean (SD) 6.86 (5.58) ; Intra-subject *p* = -0.069 Eta^2^= 0.02 • Control: E1 Mean (SD) 8.78 (6.85); E3 Mean (SD) 8.87 (6.84) Inter-subject *p* = 0.370 Eta^2^= 0.01 • Time X group *p* = 0.000 Eta^2^= 0.07 EORTC Fatigue • Group: E1 Mean (SD) 2.24 (0.81) ; E3 Mean (SD) 2.08 (0.73) Intra-subject *p* = 0.834 Eta^2^ = 0.00 • Control E1 Mean (SD) 2.09 (0.68) ; E3 Mean (SD) 2.14 (0.77) • Inter-subject *p* = 0.408 Eta^2^ = 0.00 • Time X group *p* = 0.036 Eta^2^ = 0.03 A greater reduction of negative affects and improvement in positive affects and in quality of life functional or symptom scales were observed in the TG compared with the CG. This concerned the POMS fatigue (7% of the variance explained by the model including the time/group interaction term) and the EORTC QLQ-C30 fatigue (3%).
Espie 2008 [23]	FSI	150	100	50	6 months	*p* < 0.001 (Standardized Effect =− 0.82) CBT participants had reduced symptoms of fatigue relative to TAU. FSI Interference Post-Treatment • Standardized Effect - 0.81 • 95% CI − 1.20 to-0.42 • *p <* 0.001 6-Month follow-up • Standardized Effect − 0. 82 • 95% CI − 1.22 to − 0.42 • *p <* 0.001
Ferguson 2016 [24]	Functional Assessment of Chronic Illness Therapy-Fatigue [FACIT-F]	47	27	20	2 months	Memory and Attention Adaptation Training (MAAT) and Supportive Therapy (ST) participants did not differ with regard to fatigue (FACIT-F) at the post-treatment (F (1,28), 0.072; *p* = 0.79) or 2-month ((F (1,28), 2.35; *p* = 0 .14). The Cohen’s d effect sizes for, fatigue at the 2-month follow-up time point suggested that MAAT participants demonstrated sustained clinical gains compared with ST participants (0.46)
Fillion 2008 [25]	Multidimensional Fatigue Inventory	87	44	43	3 months	Marginal Group x Time interaction effects: *p* = 0 .07; Cohen *d* = 0.36 Significant time main effects: *p* = 0 .0001; Cohen *d* = 0.69 Significant group main effects: *p* = 0 .03; Cohen *d* = 0.49 Results showed that participants in the intervention group showed greater improvement in fatigue.
Foster 2016 [26]	Brief Fatigue Inventory (BFI)	159	83	76	12 weeks	T1 Group effect (95 % CI) 0.514 (− 0.084, 1.112) *p* = 0.09 T2 Group effect (95 % CI) 0.106 (− 0.427, 0.638) *p* = 0.70
Freeman 2015 [27]	FACIT-Fatigue and Scale (FACIT-F, version 4)	118	71	47	3 months	Group effect *p* value = 0.002 Time effect *p* value= 0.084 Group × time effect *p* value = 0.321 The Bonferroni method was used to correct for multiple comparisons, and alpha was adjusted to 0.01. Linear multilevel modelling analyses revealed less fatigue, cognitive dysfunction, and sleep disturbance for Live Delivery and Telephone Delivery compared with WL across the follow-up (*p*’s < 0.01). Changes in fatigue, cognitive dysfunction, sleep disturbance, and health-related and breast cancer-related QOL were clinically significant. There were no differences between LD and TD.
Gielissen 2006 [28]	Fatigue severity subscale of the CIS	98	50	48	6 months	Patients in the intervention condition reported a significantly greater decrease than patients in the waiting list condition in fatigue severity (difference, 13.3; 95% CI, 8.6 to 18.1)
Heckler 2016 [29]	Brief Fatigue Inventory (BFI)/ FACIT-F	96	47	49	7 weeks (post intervention)	CBT and placebo *p* = 0.0005 (95% CI) [− 2.22, − 0.74] CBT and placebo *p* ≤ 0.0001 (95% CI) [5.57, 12.90] CBT-I effect (95% CI) for BFI was − 1.00 (− 1.64, − 0.37), *p* = 0.0024, meaning that CBT-I led to a mean change one unit less than no CBT-I. The CBT-I effect (95 % CI) for FACIT-Fatigue was 7.16 (3.68, 10.64), *p* < 0.0001, meaning that CBT-I led to a mean change seven units higher than no CBT-I. No statistically significant change between post-intervention and follow-up; *p* = 0.294 (BFI), *p* = 0.145 (FACIT-Fatigue).
Hoffman 2012 [30]	pOMSF/I	214	103	111	12–14 weeks	There were statistically significant differences between treatment groups for POMS fatigue *p* = 0.002 [8 weeks only] Difference Between Groups at T2 adjusted for baseline mean = − 2.68; 95% CI = [− 4.31 to − 1.04] Difference between groups at T3 adjusted for baseline mean = − 1.84 95% CI = [− 3.45 to − 0.22] Interaction time X treatment group, *P* .324
Johns 2015 [49]	Fatigue Symptom Inventory	35	18	17	1 month	Significantly greater improvements in fatigue interference than wait-list controls. The magnitude of the effect of MBSR on this and other fatigue outcomes including fatigue severity and vitality was large at the end of the intervention and 1 month later. improvements in all symptoms were maintained for at least 6 months beyond the completion of the MBSR course for both groups after their respective courses. T2 FSI interference p* ≤ 0.001 Pooled SD = 1.73 Effect size =− 1.43 95% CI effect size = [1.96, − 0.90] FSI severity p* ≤ 0.001 Pooled SD = 1.64 Effect size =− 1.55 95% CI effect size = [− 2.09, − 1.01] T3 FSI interference p* ≤ 0.001 Pooled SD = 2.01 Effect size=− 1.34 95% CI effect size = [1.88, − 0.81] FSI severity p* ≤ 0.001 Pooled SD = 1.51 Effect size =− 1.54 95% CI effect size = [− 2.10, − 0.97]
Lengacher 2012 [31]	Symptom Inventory (MDASI)	84	41	43	6 week	*p* < 0.5 P (between-group post-assessment) *p* = 0.05 At post-intervention, the MBSR(BC) group showed greater improvement across symptoms, and especially symptom interference items, compared to the control group. For the MBSR(BC) group, statistically-significant reductions (*p* < 0.01) were observed for fatigue.
Matthews 2014 [32]	Piper Fatigue Scale	56	30	26	6 week	*p* = 0.76 *d* = 0.2 No group differences in improvement were noted relative to fatigue.
Prinsen 2013 [33]	Checklist Individual Strength (CIS-fatigue)	37	23	14	6 months	CBT resulted in a significantly larger decrease in fatigue severity compared to a period of waiting for therapy. After 6 months of follow-up, patients who underwent CBT, with a mean of 12.0±5.0 individual sessions, showed a significantly larger change in fatigue scores than patients in the waiting list group (*p* < 0.001, respectively − 49.0 ± 23.0% and − 16.4 ± 25.0%). Baseline to follow-up (within group) *p* < 0.001 *p* = 0.022
Reeves 2017 [34]	FACIT	90	45	45	6 months	Only the intervention arm showed significantly improved Fatigue- Mean change (95% CI)= 3.0 (0.7, 5.3) *p* < 0.01 Intervention – usual care- No statistically significant intervention effects were observed Mean difference (95% CI) = 1.1 (− 2.4, 4.5) *p* = 0.527
Reich 2017/ Lengacher 2016 [35, 46]	Fatigue Symptom Inventory	303	155	148	12 weeks	MBSR(BC) demonstrated greater symptom improvement in fatigue (severity and interference; *p* < 0.01). Effect sizes (Cohen’s d) were between 0.27 and 0.23. A majority of improvements in fatigue occurred during the MBSR(BC) training, with little change occurring during the follow-up period (6 to 12 weeks). Fatigue—severity (FSI) *p* = 0.002T2 week *d* = 0.33 95% CI [0.13 to 0.54] T3 week *d* = 0.27 95% CI 12 0.07 to 0.47 Fatigue—interference (FSI) *p* = 0 .006T2 week *d* = 0.3 95% CI [0.10 to 0.51 ]T3 week *d* = 0.23 95% CI [0.02 to 0.43]
Reif 2013 [47]	Fatigue Assessment Questionnaire (FAQ) and Fatigue subscale of the EORTC-QLQ-C30	234	120	114	6 months	FAQ : Significant reduction in intervention group: (*F* = 76.510, *p* < 0.001, η2 = 0.248). The control group showed almost no change in CRF levels over time. In the repeated measures ANOVA, this difference was statistically significant for the group by time interaction (F = 76.51, *p* < 0.001). The partial η2of 0.248 indicates a large effect. QLQ-C30 fatigue subscale: the IG showed a reduction from 75.37 (19.39) to 40.74 (30.60) while the values in the CG remained about the same (*F* = 57.837, partial η2 = 0.2, *p* < 0.001). This finding confirms the results of the FAQ.
Ritterband 2012 [36]	Multidimensional Fatigue Symptom Inventory- Short Form (MFSI-SF)	28	14	14	9 weeks	*p* < 0.01 Overall adjusted ES (*d*) = 1.16 A significant group × time interaction was found for the overall measure of fatigue, MFSI-SF (*F*_1,26_ = 13.88, *p* < 0.01). Participants in the Internet group had significantly improved fatigue scores from 22.86 to 9.50 (*t*(13) = 3.63, *p* < 0.01); control participants’ scores did not improve over time, changing from 13.71 to 19.79 (*t*(13) = − 1.64, *p* = 0.12). Several MFSI-SF subscales also had significant group × time interactions, including general fatigue (*F*_1,26_ = 9.46, *p* < 0.01), mental fatigue (*F*_1,26_ = .65, *p* < 0.01), and vigor (*F*_1,26_ = 14.79, *p* < 0.01), with Internet participants showing improvements compared with control participants in all cases. Although some subscales lacked significant group × time interactions (physical fatigue, *p* = 0.11; emotional fatigue, *p* = 0.08), adjusted ES for the fatigue variables ranged from a low of 0.47 to a high of 1.63, indicating a SHUTi treatment effect for fatigue.
Rogers 2017 [37]	Fatigue Symptom Inventory	222	110	112	3 months	BEAT Cancer significantly reduced fatigue intensity at both time points (mean between group difference [M] = − 0.61; 95% CI = − 1.04 to − 0.19; effect size [*d*] = − 0.32; *p* = .004 at M3 and M = − 0.46; 95% CI − 0.89 to − 0.03; *d* = − 0.26; *p* = .038 at M6). Significant and greater reductions in fatigue interference occurred (M = − 0.84; 95% CI = − 1.26 to − 0.43; *d* = − 0.40; *p* < .001 at M3 and − 0.66; CI − 1.08 to − 0.24; *d* = − 0.35; *p* = .002 at M6).
Sandler 2017 [38]		46	22	24	24 weeks	Fatigue severity improved in all subjects from a mean of 5.2 (− 3.1) at baseline to 3.9 (− 2.8) at 12 weeks, suggesting a natural history of improvement. Clinically significant improvement was observed in 7 of 22 subjects in the intervention group compared with 2 of 24 in the education group (*p* < 0.05) The whole cohort reported improvements in fatigue scores between baseline and 12 weeks (Mdiff = − 1.27; 95% CI − 2.52 to − 0.03; *p* < 0.05) and 24 weeks (Mdiff = − 1.51; 95% CI − 2.84 to − 0.18; *p* < 0.05). Change scores differed significantly in favour of the intervention (M = 2.55, SD = 3.77; *t*(36) = − 2.56; *p* < 0.05) at 12 weeks in comparison to the education arm (M = 0.10; SD = 2.55) but not at follow up (Mdiff = 1.56; 95% CI − 3.77 to 0.48; *p* = 0.13). These groupwise changes indicate an effect size in the CBT/GET group of *d* = 0.79, compared with *d* = 0.04 in the education arm.
Savard 2005 [39]	Multidimensional Fatigue Inventory (MFI)	57	27	30	12 months	Pooled data revealed significant differences between pre- and post-treatment on fatigue (*F*1,158 = 11.70; *p* < .001), No significant difference was detected between post-treatment and the follow-up evaluations. Therapeutic effects were well maintained up to 12 months after the intervention and generally were clinically significant. Pooled data (*n* = 57) 3-month follow-up : adjusted mean= 2.33; 95% CI = 2.15 to 2.51 6-month follow-up: adjusted mean = 2.25; 95% CI = 2.07 to 2.43 12-month follow-up: adjusted mean = 2.18; 95% CI = 1.98 to 2.38
Van Der Lee 2012 [40]	Multidimensional Fatigue Inventory (MFI)- General fatigue	83	59	24	6 months	*p* < 0.001 At post-treatment measurement the proportion of clinically improved participants was 30%, versus 4% in the waiting list condition (*Χ*^2^ (1) = 56.71; *p* = 0.007). The mean fatigue severity score at post-measurement was significantly lower in the intervention group (95%CI = 33.2–37.9) than in the waiting list group (95% CI = 40.0–47.4) controlled for pre-treatment level of fatigue. The effect size for fatigue is 0.74 (*d* = (mean post intervention–mean post control)/pooled SD). The treatment effect was maintained at 6-month follow-up. At follow up 39% of the participants in the intervention group showed clinically relevant improvement in fatigue severity.
Van Weert 2010 [41]	Multidimensional Fatigue Inventory (MFI)- General fatigue	209	76	133	12 weeks	In comparison with the WLC group, the PT group showed more reduction in 4 domains of fatigue, whereas the PT+CBT group showed more reduction in one domain only. Finally, the results showed that physical training combined with CBT and physical training alone were equally effective in reducing fatigue. Thus, CBT did not seem to contribute additional positive effects on fatigue to the benefits of physical training. PT + CBT (WLC = Reference) between-group change General fatigue (95% CI) = − 1.3 (− 3.1 to 0.4) Physical fatigue (95% CI) = − 2.7 (− 4.5 to − 1.0) *p* < 0.01. Mental fatigue (95% CI) = − 0.5 (− 2.3 to 1.2) Reduced motivation (95% CI) = − 0.6 (− 2.1 to 1.0) Reduced activation (95% CI) = − 0.9 (− 2.6 to 0.8)
Willems 2017 [48]	Fatigue severity subscale of the CIS	409	188	221	6 months 12 months	The intervention was effective in reducing fatigue (B =-4.36, p = 0.020, d = 0.21). Adjusted: 6 months *p* = 0.030 95% CI [− 7.87 to − 0.39] (*d* = 0.21) Adjusted: 12 months *p* = 1.000 95% CI [− 3.88 to 3.88] (*d* = 0.04) Between- group differences at 12 months from baseline on emotional ( *p* = .611, *d* = 0.04) were non-significant The intervention group remained fairly stable in fatigue between 6 and 12 months from baseline, but the control group slightly improved over time, leading to non-significant group differences at 12 months from baseline.
Yun 2017 [42]	EORTC QLQ-C30 fatigue score	174	57	117	12 months	From baseline to 12 months, the LP group, relative to the UC group, showed a significantly greater decrease in the EORTC QLQ-C30 fatigue score (*p* = 0.065) 3 months: *p* = 0.214 12 months: *p* value = 0.010**
Yun 2012 [43]	Brief Fatigue Inventory (BFI) and Fatigue Severity Scale (FSS)	273	136	137	3 months	BFI: *p* < 0.01 95% CI − 1.04 to-0.27 Cohen’s *d* = 0.29 FSS: *p* < 0.01 95% CI − 0.78 to − 0.21 Cohen’s *d* = 0.27 Compared with the control group, the intervention group had an improvement in fatigue as shown by a significantly greater decrease in BFI global score (-0.66 points; 95% CI − 1.04 to − 0.27) and FSS total score (− 0.49; 95% CI − 0.78 to − 0.21).

In cases where more than one paper was published relating to the same study, the papers were assigned to one study. Five articles were found in the grey literature and full-texts were not available online. Study authors of each of these papers were contacted. Three study authors provided full-texts in preparation for publication. The other two papers were excluded at this point, as full-texts were not available. No articles were found in snowball search.

### Description of included studies

Data were extracted from the included papers (see Table 2. for a description of the included studies). The 33 RCTs reported data on 4486 cancer survivors (2196 intervention and 2290 controls). The majority of studies were conducted in the USA [14–17, 20, 21, 24, 27, 29, 31, 32, 35–37, 49, 50]. Six were carried out in the Netherlands [18, 28, 33, 40, 41, 48], three in the UK [23, 26, 30]. The remainder were conducted in Australia, [34, 38] Canada [39, 45], Germany [47], France [22, 25] and Korea [42, 43].

### Participants

As per the inclusion criteria for this review, studies were required to include only those who have completed active medical treatment prior to taking part in the research. However, there was little consistency across the studies regarding the timing of the intervention in relation to time elapsed since completion of cancer treatment.

### Interventions

Details of interventions can be seen in Table 2, including content, strategies employed, mode of delivery, duration, who delivered the intervention and the comparison or control group used. Twelve studies reported on the effects of a CBT intervention [20, 23, 24, 28, 29, 32, 33, 36, 38, 39, 41, 43], of which six were focused specifically on CBT for insomnia (CBT-i) [20, 23, 29, 32, 36, 39]. Over half of these (*n* = 5) were studies on CBT-I [20, 23, 29, 36, 39]. Two of the CBT interventions were combined with physical activity [38, 41]. Other studies incorporated CBT strategies into the intervention. Dolbeault et al. [22] reported on a psycho-educational intervention based on CBT and another study reported on a trial of Cognitively-Based Compassion Training [21]. Van der Lee et al. used a combination of CBT and mindfulness strategies in a trial on mindfulness-based cognitive therapy [40].

Seven studies [16, 17, 19, 30, 31, 35, 49] reported on mindfulness-based interventions. Two of the studies were specifically aimed at CrF [30, 49], and three were focused on cancer [16, 19, 35].

Bruggeman-Everts [18] compared ambulant activity feedback (AAF) and psychologist-guided web-based mindfulness-based cognitive therapy groups to a psychoeducational group, showing that the psycho-education group was least effective at reducing fatigue. Other interventions included a patient education program [47], a physical activity behaviour change intervention [37] and a combined psycho-education and physical activity intervention [25]. Health coaching and motivational interviewing was employed in two studies [15, 42]. Freeman et al. tested an imagery-based intervention [27]. Three studies reported on lifestyle interventions [14, 34, 51] and one online intervention aimed to enhance self-efficacy to manage problems associated with cancer-related fatigue following primary cancer treatment [26].

### Control group

There was substantial heterogeneity in the comparison groups used within the trials. See Table 2 for further details

### Outcomes

#### Primary outcomes

A variety of different measures were used to assess fatigue. The Brief Fatigue Inventory (BFI) was used in five studies [14, 17, 26, 29, 43] and the Functional Assessment in Cancer Therapy-Fatigue (FACIT-F) was used in five studies [16, 24, 27, 29, 34]. Five studies used the Fatigue Symptom Inventory (FSI) [17, 23, 35, 37, 49] and the Multidimensional Fatigue Inventory (MFI) was used in four studies [25, 39–41]. Ritterband [36] used the short form of the Multidimensional Fatigue Symptom Inventory-Short Form(MFSI-SF). The Schwartz Cancer Fatigue Scale was used in one study [15]. Four studies [18, 28, 33, 48] employed the Checklist Individual Strength (CIS). The remaining studies used fatigue subscales of broader multi-dimensional measures. Three studies assessed fatigue using two different questionnaires. Yun et al. [43] used both the BFI and the Fatigue Severity Scale (FSS), whereas another study used the BFI in conjunction with the FACIT-F [29]. The third study used both the Fatigue Assessment Questionnaire (FAQ) and fatigue subscale of the EORTC-QLQ-C30 [47].

#### Secondary outcomes

Secondary outcomes of interest to this review were specified a priori in the study protocol [12] and are summarised in Additional file 1. These included mood (self-reported scales of depression, and/or anxiety or distress); global quality of life and functional impact of fatigue (self-report questionnaire measures assessing the impact of fatigue on quality of life and daily functioning); and fatigue self-efficacy. Most of the studies included a measure of mood, either as an outcome or a control variable. However, the mood outcomes were assessed by a wide range of psychometric tools which assessed various dimensions of mood including stress, depression, anxiety and distress. Many of the studies also included a measure of global quality of life (QoL) and functional impact of fatigue. Only two of the studies assessed self-efficacy in relation to coping with fatigue [25, 26].

In the review process, other frequently reported secondary outcomes that were not outlined in the review protocol were identified as relevant to this review. These outcomes of interest were insomnia or sleep quality and pain. Studies that assessed sleep quality or insomnia tended to be designed with the aim of impacting insomnia or quality of life after cancer treatment.

As with the measures used to assess fatigue, a variety of measures were used to assess mood-related variables, with some studies including more than one measure of mood. The most commonly used measures were the Hospital Anxiety and Depression Scale (HADS) [52], The Patient Health Questionnaire (PHQ) [53] (a measure of depression severity) and The Profile of Mood States (POMS) [54] (a measure of psychological distress). The State-Trait Anxiety Inventory (STAI) [55] was also used.

The two most commonly used scales to assess quality of life were the European Organisation for the Research and Treatment of Cancer Quality of Life Questionnaire Core 30 (EORTC QLQ-C30) [56] and the Functional Assessment of Cancer Therapy-General (FACT-G) [57]. In the study protocol, the reviewers aimed to delineate the concepts of ‘global quality of life’ and ‘functional impact of fatigue’ [12]. However, in line with Luckett et al. [58], this was not deemed appropriate in the final review. Both types of measures assess physical, emotional, social, and functional/role scales. The QLQ-C30 provides brief scales for cognitive functioning, financial impact and a range of symptoms either not assessed by the FACT-G or else subsumed within its well-being scales. The FACT-G includes both symptoms and concerns within each scale [58]. The Medical Outcomes Study (MOS) [59], Sickness Impact Profile (SIP) [60], the SF-12 [61] and the M.D. Anderson Symptom Inventory (MDSAI) [62] were also used.

A variety of outcome measures were also used to assess sleep quality or insomnia. The Insomnia Severity Index (ISI) [63] was the most commonly used. Other measures included the Women’s Health Initiative Insomnia Rating Scale (WHIIRS) [64] and the Pittsburgh Sleep Quality Index (PSQI) [65]. Broader QoL measures that assessed insomnia/sleep quality included the MDSAI [62] and the EORTC QLQ-C30 [56].

### Risk of bias assessment

The included studies were assessed for risk of bias using the Cochrane ‘Risk of Bias’ Tool [44]. Some aspects of the studies were not reported with sufficient detail to assess bias and therefore were rated as unclear risk of bias for domains where insufficient information was provided. Further details are presented in Additional file 2.

#### Random sequence generation (selection bias)

Most studies described the process of allocating participants between study groups randomly, providing details about the method of randomisation employed. Eight studies did not describe random sequence generation in enough detail to allow a definite judgement.

In the majority of studies (*n* = 24), the method of allocation concealment either was not described or not described in sufficient detail to allow a definite judgement.

#### Blinding (performance bias and detection bias)

Most of the trials included in this review were at high risk of performance bias because, owing to the nature of the intervention, it was not possible to blind the trial personnel and participants. In a number of the studies were not described in sufficient detail to allow a definite judgement as to whether or not outcome assessors were blinded about the group allocation of participants.

#### Incomplete outcome data (attrition bias)

All studies provided some details of study attrition. Many of the studies (*n* = 19) were at a low risk of attrition bias, with good completion rates.

#### Selective reporting (reporting bias)

The majority of studies were at a low risk of reporting bias as, based on the information provided by the trial authors and study protocols (where available), it was unlikely that there was selective reporting of the primary and secondary outcomes. Sixteen of the trials were provided trial registration details.

#### Other bias

Most trials were deemed to be at a low risk for other biases such as potential bias due to baseline differences, inappropriate influence of the study sponsor and early stopping for benefit [12].

### Quality of the evidence

We employed the GRADE approach to assess the evidence for the primary comparison of ‘Psychological Interventions compared to usual care for Fatigue in cancer survivors’. As seen in Table 4, the majority of the evidence relating to psychological interventions for fatigue is of low quality, largely due to the finding that the available evidence is too heterogeneous to pool across studies. Further, it due to incomplete reporting of methods, it was difficult to ascertain risk of bias in studies. There is little evidence that directly answers the questions of interest for different types of psychological therapies.

**Table 4:** Grade evidence summary

Outcomes	№ of participants (studies)	Certainty of the evidence	Explanations
Psychological Interventions compared to usual care for Fatigue in cancer survivors			
Follow up: range 2 weeks to 1 years Intervention: Psychological Interventions Comparison: usual care	2918 (22 RCTs)	⨁⨁◯◯LOW ^a, b^	a. Downgraded x 1 level for risk of bias due to all studies having high or unclear risk of performance bias. Many aspects of trial procedures were not reported in sufficient detail to adequately assess risk of bias in all domains of all included trials (e.g. unclear risk of selection bias in 18/22 studies, unclear risk of detection bias in16/22). b. Downgraded x1 level for indirectness of evidence as many studies were combined interventions, which limit our ability to draw conclusions in relation to our research question relating solely to the effectiveness of psychological interventions. Generalizability of the findings are limited due to the high proportion of studies that recruited only/mostly breast cancer survivors. The majority of studies did not specifically target fatigue or screen for fatigue as part of inclusion criteria as recommended in existing guidelines. In some studies, it was difficult to assess when exactly participants completed cancer treatment prior to participating in the study. High levels of heterogeneity in sample and methods.
Subgroups of specific psychological intervention type (e.g. cognitive behavioural therapy) vs usual care			
CBT interventions compared to usual care for Fatigue in cancer survivors Follow up: range 1 months to 1 years	648(8 RCTs)	⨁⨁◯◯LOW^a, b^	a. Downgraded x 1 level for risk of bias due to high/ unclear risk due to incomplete outcome data (attrition bias) in 5 of 8 studies Many aspects of trial procedures were not reported in sufficient detail to adequately assess risk of bias. b. Downgraded x1 level for indirectness of evidence as high levels of heterogeneity in sample and methods that limit the generalizability of the findings- While CBT was incorporated in all interventions to some degree, it was delivered in a variety of settings, modes and assessed in different ways. For example, 3 x studies were not CBT interventions but were based on CBT strategies and 3x studies were focused specifically on CBT for insomnia.
Mindfulness-based interventions compared to usual care for Fatigue in cancer survivors Follow up: range 1 months to 4 months	749(6 RCTs)	⨁⨁◯◯LOW ^a, b^	a. Downgraded x 1 level for risk of bias due to high or unclear risk of performance bias in all studies. Many aspects of trial procedures were not reported in sufficient detail to adequately assess risk of bias. b. Downgraded x1 level for indirectness of evidence as high levels of heterogeneity in sample and methods that limit the generalizability of the findings- While mindfulness was incorporated in all interventions to some degree, it was delivered in a variety of settings, modes and assessed in different ways.
Other psycho-social interventions compared to usual care for Fatigue in cancer survivors Follow up: range 3 months to 12 months	1521(8 RCTs)	⨁⨁◯◯LOW ^a, b^	a. Downgraded x 1 level for risk of bias due to high or unclear risk of performance bias in all studies Some aspects of trial procedures were not reported in sufficient detail to adequately assess risk of bias b. Downgraded x1 level for indirectness of evidence as high levels of heterogeneity - While all were psychological interventions, they were vastly different in sample and methods. Further, 4 x studies were lifestyle interventions that incorporated other interventions such as physical activity and dietary changes.

GRADE Working Group grades of evidence

High certainty: We are very confident that the true effect lies close to that of the estimate of the effect

Moderate certainty: We are moderately confident in the effect estimate: The true effect is likely to be close to the estimate of the effect, but there is a possibility that it is substantially different

Low certainty: Our confidence in the effect estimate is limited: The true effect may be substantially different from the estimate of the effect

Very low certainty: We have very little confidence in the effect estimate: The true effect is likely to be substantially different from the estimate of effect

### Effects of interventions

In the published protocol, we had planned to conduct a meta-analysis, if it was deemed clinically meaningful and appropriate to do so [12]. However, given the heterogeneity in participant groups, study design, study comparators and measures used, we synthesised data narratively, as a meta-analysis would have been inappropriate.

#### Comparison 1: psychological interventions (all types) vs. usual care

##### Primary outcome: fatigue

Eleven psychological interventions reported a significant effect of the intervention on an outcome of fatigue, compared to a waitlist control or usual care [17, 23, 28, 31, 33, 35, 36, 39, 42, 47, 49].

##### Secondary outcomes

1. Global quality of life (QoL)/functional impact of fatigue
  Global QoL/functional impact of fatigue was assessed in 19 of the 22 studies that compared a psychological intervention to a waitlist control or usual care. Thirteen of these 19 studies demonstrated a significant improvement compared to the control group, in at least one measure of QoL/functional impact of fatigue [22, 23, 25, 28, 30, 31, 33, 39, 40, 43, 47–49]. One study reported that participants assigned to the intervention group had significantly lower physical well-being compared to the control group at follow-up [21]. The remaining studies did not report any Group X Time interaction effects [15, 26, 34–36].
2. Fatigue self-efficacy
  Two studies assessed fatigue self-efficacy. Bower et al. [17] used the fatigue subscale of the HIV self-efficacy questionnaire and reported that Intervention group participants were significantly more confident than control group participants about their ability to manage fatigue and its impact on their lives at follow-up [17]. Foster et al. assessed fatigue using the Perceived Self-efficacy for Fatigue Self-management (PSEFSM). Initial evidence of improved fatigue self-efficacy at T1 in the intervention group was not maintained at final follow-up [26].
3. Mood
  Mood was assessed over time in 18 of the 22 studies that compared a psychological intervention to a waitlist control or usual care. Ten of these reported significant improvements compared to the control group, in at least one measure of mood over time [21–23, 31, 35, 39, 43, 47, 49].
4. Sleep/insomnia
  Sleep/ insomnia was assessed over time in 12 of the 22 studies that compared a psychological intervention to a waitlist control or usual care. Nine of these reported significant improvements compared to the control group, in at least one measure of sleep quality or insomnia symptoms over time [14, 16, 23, 31, 35, 36, 39, 47, 49]. Three of these studies were designed to specifically target insomnia or sleep disturbance—all were effective for reducing fatigue [23, 36, 39].

### Subgroup analysis and investigation of heterogeneity

In the original protocol, we specified that we would explore effects by subgroups of specific psychological intervention type (e.g. cognitive behavioural therapy) vs usual care.

#### Comparison 2: subgroups of specific psychological intervention type (e.g. cognitive behavioural therapy) vs. usual care

##### Cognitive-behavioural therapy vs. usual care

Five studies reported on the effects of a CBT intervention compared to waitlist control or usual care [23, 28, 33, 36, 39], of which three were focused specifically on CBT for insomnia (CBT-i) [23, 36, 39].

##### Primary outcome: fatigue

Each of the five CBT studies reported significant effect of the intervention on fatigue over time [23, 28, 33, 36, 39]. Two other studies incorporated CBT strategies into the intervention. Dolbeault et al. [22] reported a significant effect on fatigue of a psycho-educational intervention based on CBT. Another study reported no significant differences between groups on a trial of Cognitively-Based Compassion Training [21]. Van der Lee et al. reported a significant effect of intervention over time using a combination of CBT and mindfulness strategies in a trial on mindfulness-based cognitive therapy [40].

##### Secondary outcomes

1. Global quality of life/functional impact of fatigue
  Four of the five CBT studies reported significant effect of the intervention over time at least one measure of Global QoL/functional impact of fatigue [23, 28, 33, 36, 39]. Savard et al. reported a significant group-time interaction global quality of life using the EORTC QLQ-C30 [39]. Using the Functional Assessment of Cancer Therapy Scale-general (FACT-G), Espie et al. [23] reported that CBT was associated with increased physical and functional QoL compared to the control group, at post-treatment and at follow-up. Using the SIP-8, both Prinsen et al. [33] Gielissen et al. [28] stated that the intervention condition reported a significantly greater decrease than patients in the waiting list condition in functional impairment. Ritterband et al. [36] reported that the group x time interaction for either the physical or mental subscale of the SF-12 was not significant.
  Using the EORTC core quality of life questionnaire (EORTC QLQ-C30), Dolbeault et al. reported greater improvement in emotional functioning, role functioning and global health status scales in the CBT-based psycho-educational intervention group compared with the control group. Group × time interaction effects were non-significant for the other subscales of the EORTC [22]. Using the SIP-8, van der Lee et al. reported that 6 months after the intervention, the mean well-being score at post measurement was significantly higher in the mindfulness-based cognitive therapy intervention group than in the waiting list group corrected for pre-treatment level of well-being [40]. Conversely, participants assigned to cognitively-based compassion trainingh had significantly lower physical well-being compared to the control group at follow-up [21].
2. Fatigue self-efficacy
  None of the five CBT studies assessed fatigue self-efficacy.
3. Mood
  Mood was assessed over time in four of the five studies that compared a CBT intervention to a waitlist control or usual care [23, 28, 36, 39]—three of these reported a significant effect of the intervention on mood [23, 28, 39]. Gielissen et al. [28] assessed psychological distress using the Symptom Check List 90 and found that participants in the intervention condition reported a significantly greater decrease in psychological distress (95% CI, 12.7 to 30.4, *p* < 0.001) than patients in the waiting list condition. Using the Hospitals Anxiety and Depression Scale [HADS], Espie et al. [23] reported that CBT participants had reduced symptoms of anxiety, and depression relative to the control group (anxiety 95% CI − 0.92 to − 0.12, *p* = 0.011; depression 95% CI − 0.99 to − 0.19, *p* = 0.004). Also using the HADS, Savard et al. [39] reported significant group-time interactions on scores of anxiety (*p* < .05) and depression (*p* < .05). In contrast, Ritterband et al. [36] reported that the group × time interaction was not significant (*p* = .09) on the total HADS score.
  Dolbeault et al. [22] reported that a greater reduction of negative affect and improvement in positive affect was demonstrated in the intervention group compared with the control group. Significant group × time interactions indicated a positive effect of the intervention on anxiety, measured using the State-Trait Anxiety Inventory. Psychological adjustment—assessed with the Profile of Mood States (POMS)—demonstrated group × time interactions in favour of the intervention on anxiety, anger and depression. No effect of the intervention group was evidenced on The Mental Adjustment to Cancer Scale (MAC).
  Dodds et al. [21] reported that compared to controls, at follow-up, participants assigned to the CBCT group demonstrated had significantly lower levels of perceived stress in the past week (− 1.6, 95 % CI − 3.1, − 0.2)—assessed using the Perceived Stress Scale (PSS-4). The Cognitive and Affective Mindfulness Scale-Revised (CAMS-R 10) demonstrated enhanced mindful presence in participants assigned to the CBCT group compared to controls, at follow-up (3.1, 95 % CI 0.4, 5.8). There was no significant impact of the intervention on the other mood scales at final follow-up (week 12): Brief Center for Epidemiologic Studies—Depression questionnaire (CES-D-10), Fear of Cancer Recurrence Inventory (FCRI), the Impact of Events Scale—Revised (IES-R) or UCLA Loneliness Scale Version 3 (R-UCLA).
4. Sleep/insomnia
  Sleep/insomnia was assessed over time in four of the five studies that compared a CBT intervention to a waitlist control or usual car e[22, 23, 36, 39]—three of these reported significant improvement compared to the control group, in at least one measure of sleep quality or insomnia symptoms over tim e[23, 36, 39].
  Using the Insomnia Interview Schedule Insomnia Severity Index, Savard et al. [39] reported significant group-time interactions for all self-reported sleep variables, except for total sleep time. These included sleep efficiency, total wake time, sleep onset latency, wake after sleep onset.
  Ritterband et al. [36] also employed the Insomnia Severity Index and reported a significant group × time interaction effect with the intervention group showing a significant improvement in insomnia severity from pre- to post-assessment, compared to the control group. These improvements were also clinically significant. Sleep Diary Variables were also used to assess sleep efficiency, sleep onset latency, wake after sleep onset and total sleep time. A significant group × time interaction was found for sleep efficiency and sleep onset latency with medium-to-large treatment effects (*d* = .72 and *d* = .67 respectively). There was not a significant group x time interaction for wake after sleep onset, time in bed, number of awakenings or total sleep time. The intervention group also showed significantly more improvements than those in the control group on soundness of sleep and feeling restored, with large effect sizes (1.21 and 1.35, respectively).
  Espie et al. [23] also used sleep diaries to assess difficulty initiating (SOL) and maintaining (WASO) sleep. Changes in total sleep time were not statistically significant, but improvements were seen in the CBT group WASO, SOL and Sleep efficiency scores. CBT was associated with median reduction in insomnia symptoms of almost 1 h (SOL + WASO) compared with no change in the control group.
  Dolbeault et al. [22] reported that no effect of the intervention group was evident over time, assessed using the EORTC QLQ-C30 sleep.

### Mindfulness-based interventions

Six studies compared mindfulness-based interventions to waitlist control or usual care [16, 17, 30, 31, 35, 49]. Two of the studies were specifically aimed at CrF [30, 49] and another two were specifically focused on cancer [16, 35].

#### Primary outcome: fatigue

Four of the studies on mindfulness-based interventions reported a significant effect of intervention on fatigue over time [17, 31, 35, 49]. One of the effective studies one was specifically aimed at CrF [49] and one was specifically focused on cancer [35]. The effective findings were not maintained at final follow up in one of the studies [17].

#### Secondary outcomes

1. Global quality of life/functional impact of fatigue
  Four of the mindfulness assessed Global QoL/functional impact of fatigue [30, 31, 35, 49]. Three reported significant effect of the intervention over time on at least one measure of Global QoL /functional impact of fatigue [30, 31, 49]. Hoffman et al. [30] employed the breast-specific quality of- life scale FACT-B and the FACT-ES scale for endocrine symptoms and reported that mean scores in the intervention group were greater at both 8 and 12 weeks compared with the control group for all six measures (except social well-being which was significant at 8 weeks only). Using the WHO five-item well-being questionnaire (WHO-5), Hoffman et al. also reported significant increases in the intervention group compared with controls at both timepoints [30]. The authors also noted that increased hours of formal mindfulness classroom and home practice in the intervention group was associated with improved scores in FACT-ES, FACT-B, FACT physical well-being and WHO-5 at 12 weeks. Johns et al. assessed functional status using the Sheehan Disability Scale (SDS) and reported that the MBSR group demonstrated significantly lower functional disability scores than the control group at final follow-up with a large effect size (*d* = − 1.22) [49]. Lengacher et al. used the M.D. Anderson Symptom Inventory (MDASI) [31]. They reported significant improvements in favour of MBSR(BC) in the symptom interference items (i.e., general activity, work (including work around the house) relations with other people, walking) and Housework, and Relationships. Using the Medical Outcomes Study Short-Form 36 (SF-36, v.2), Reich et al. [35] reported that group × time interaction was not significant for either mental or physical health.
2. Fatigue self-efficacy
  Bower et al. used the fatigue subscale of the HIV self-efficacy questionnaire and reported that Intervention group participants were significantly more confident than control group participants about their ability to manage fatigue and its impact on their lives at follow-up [17].
3. Mood
  Mood was assessed over time in each of the six studies that compared mindfulness-based interventions to waitlist control or usual care [16, 17, 30, 31, 35, 49]—three of these reported a significant effect of the intervention on mood [31, 35, 49]. In the study by Reich et al. [35, 46], patients in the MBSR(BC) group showed significantly greater improvements in anxiety (*p* = .007) assessed using the State-Trait Anxiety Inventory, and FORs (overall and problems; *p* <.01), as measured using the Concerns About Recurrence Scale. Results for depression (measured using CES-D) showed that participants assigned to MBSR(BC) tended to report greater improvement than those in usual care; however, this trend did not reach statistical significance. The authors confirmed that improvement in both the cluster of psychological symptoms (anxiety, depression, perceived stress and quality of life (QOL), emotional well-being) (*p* = 0.007) was related to assignment [35]. Lengacher et al. [31] assessed mood, enjoyment of life, distress and sadness, using the MDASI [62]. The MBSR(BC) intervention showed an improvement in mood, but not in distress or sadness. Johns et al. [49] assessed anxiety using the Patient Health Questionnaire Generalized Anxiety Disorder Scale—the MBSR group demonstrated significantly lower anxiety scores than the control group with a large effect size (*d* = − 0.98).Depression scores (measured using PHQ-8) were also significantly lower with large differences at final follow-up (*d* = − 1.71) [49].
  Using the Beck Depression Inventory-II (BDI-II), Bower et al. [17] found that a significant Group x time interaction at post-treatment was not maintained at 3 month follow-up. Stress decreased over the assessment period in both groups, as measured using the Perceived Stress Scale (PSS). Hoffman et al. [30] reported statistically significant improvements in outcome in the MBSR group compared with control group at both 8 and 12 weeks (for POMS total mood disturbance). The subscales of anxiety, depression showed these effects only at 8 week follow-up. Anger was significantly improved at 12 weeks but not at 8 weeks. The authors found that increased hours of formal mindfulness classroom and home practice in the MBSR group was associated with improved scores in POMS total mood disturbance [30]. Using the State Trait Anxiety ( STAI), Blaes et al. [16] found no significant difference between groups in anxiety despite a trend towards improvement for MBCR.
4. Sleep/insomnia
  Sleep/insomnia was assessed over time in three studies that compared mindfulness-based interventions to waitlist control or usual care—two of these reported a significant effect of the intervention on sleep/insomnia over time [16, 49]. Two of the studies assessed sleep quality using the Pittsburgh Sleep Quality Index (PSQI). Blaes et al. [16] reported that total sleep quality improved in those who received MBCR compared to those in the control group—this was maintained at 4 months. Conversely, Bower et al. [17] reported no significant effects for subjective sleep quality. Johns et al. [49] used the Insomnia Severity Index and reported that sleep disturbance was significantly improved for intervention group compared with the control condition at both follow-up points.

### Other psycho-social interventions vs. usual care

The eight remaining interventions incorporated psycho-education, motivational strategies and lifestyle and behaviour change approaches [14, 15, 25, 26, 43, 47, 51].

#### Primary outcome: fatigue

A patient education program was reported to have improved fatigue [47], while a combined psycho-education and physical activity intervention showed that participants in the intervention group showed greater improvement in fatigue, but this was not a significant effect [25]. Health coaching was found to lead to a significant reduction on fatigue at 12 months but not at 3 months [42] and an intervention employing Motivational interviewing showed no significant differences between groups at 6 months [15]. Lifestyle interventions did reported mixed findings regarding their impact on fatigue, with one [14, 34] reporting no significant differences between groups and one a significant effect of intervention at 6 months that was not maintained at 12 months [51]. An online intervention that aimed to enhance self-efficacy to manage problems associated with cancer-related fatigue following primary cancer treatment reported no significant changes in fatigue [26].

#### Secondary outcomes

1. Global quality of life /functional impact of fatigue
  Seven of the trials on other psycho-social interventions reported on Global QoL/functional impact of fatigue [15, 25, 26, 43, 47, 51]. Four reported significant effect of the intervention over time on at least one measure of Global QoL/functional impact of fatigue [25, 43, 47, 48]. Using the SF-36, Bennett et al. [15] noted that group × time interaction was not significant for either mental or physical health. Fillion et al. [28] reported marginal group × time interaction effects for physical quality of life in favour of the intervention group using the Medical Outcomes Study Short Form 12-Item Health Survey (SF-12). While mental quality of life showed no interaction or main effects, both conditions improved overtime. Conversely, there was no effect on the intervention on mental well-being.
  Three studies used the EORTC core quality of life questionnaire (EORTC QLQ-C30). In the study by Reif et al. [47], all functional and symptom scale values as well as single items values increased significantly in the intervention compared to the control group. Willems et al. also reported that the intervention was effective in increasing emotional and social functioning at 6 months [48]; however, these findings were not maintained at 12 months [51]. Similarly, Yun et al. [43] reported a significantly greater increase in global QOL and in emotional, cognitive and social functioning scores of EORTC QLQ-C30 scales. However, significance was lost on the emotional, and social functioning scores after Bonferroni corrections were applied for 15 multiple comparisons. Using the Functional Assessment of Cancer Therapy Scale-general (FACT-G), Foster et al. [26] did not report a significant effect of the intervention over time on the Fact-G measure.
2. Fatigue self-efficacy
  Foster et al. did not reported improved fatigue self-efficacy at final follow-up [26].
3. Mood
  Mood was assessed in six of the seven studies reporting on other psycho-social interventions [14, 25, 26, 43, 47, 51]. Yun et al. [43] reported that the web-based intervention group had clinically more meaningful improvement than the control group in HADS anxiety score. However, a statistically significant greater decrease in HADS was lost after Bonferroni corrections were applied. Willems et al. reported that another online intervention was effective in reducing HADS depression scores at 6months [48], but at 12 months from baseline, the intervention group no longer differed from the control group [51]. Reif et al. [47] also used the HADS and reported group × time interactions in favour of the intervention group for both anxiety and depression. Both Foster et al. [26] and Bantum et al. [14] reported a non-significant difference in groups in change over time using the Patient Health Questionnaire (PHQ-8). Fillion et al. [25] reported that no interaction effects for emotional distress (POMS anxiety + depression) were found.
4. Sleep/insomnia
  Sleep/insomnia was assessed in three of the seven studies reporting on other psycho-social interventions [14, 43, 47]. Reif et al. reported an improvement in the intervention group, compared to the control group using the EORTC QLQ-C30 insomnia subscale [47]. Using the Women’s Health Initiative Insomnia Rating Scale (WHIIRS), Bantum et al. [14] reported that the intervention group showed reduced insomnia from baseline to 6 months compared to the control group. Finally, Yun et al [43] did not report a significant effect of the intervention on scores on the Medical Outcome Study–Sleep Scale (MOS-SS) Sleep Quality Index I and II.

### Further investigation of heterogeneity in trials comparing psychological interventions (all types) vs. usual care

In the original protocol, we hypothesised that each of the factors below has the potential to have a clinically meaningful effect on the response to a psychological intervention amongst fatigued post-treatment cancer survivors.

1. Intervention for specific cancer type only vs intervention for any cancer type
2. In-person interventions vs remote interventions
3. Interventions specifically designed to treat fatigue after cancer treatment vs interventions not specific for fatigue

We performed narrative assessment of the influence of these factors on the primary outcomes. This narrative synthesis did not reveal any clear patterns in the findings based on differential influences of these factors on the effect of psychological interventions on fatigue.

#### Comparison 3: intervention for specific cancer type only vs. intervention for any cancer type

In a previous Cochrane review [8], it was noted that many of the studies of fatigued cancer patients during cancer included only breast cancer patients. *Nine of the effective* interventions in this review only included breast cancer patients. Seven studies that focused on breast cancer did not report a reduction in fatigue. Of the 17 studies with mixed samples, 13 reported a significant reduction in fatigue. However, breast cancer patients were often overrepresented in the studies of mixed samples. For example, one study [42] noted that over 60% of their sample had had breast cancer*.* Most studies included participants who had received a variety and combinations of cancer treatments (e.g. surgery, chemotherapy, radiotherapy). In one study [15], the authors specified that targeted patients were those who had received only radiotherapy.

#### Comparison 4: in-person interventions vs. remote interventions

Sixteen of the 22 trials compared that compared a psychological intervention to waitlist control or usual care were delivered in a group setting [16, 17, 21–23, 25, 30, 31, 33, 35, 39, 40, 42, 47, 49], with 11 of these reporting a reduction in fatigue over time [17, 22, 23, 31, 33, 35, 39, 40, 42, 47, 49]. The majority of the group interventions had 6–9 weekly 1–2.5 h sessions. Six included some homework or home practice [17, 21, 30, 31, 35, 49], with four of these studies reporting an effective reduction on fatigue [17, 31, 35, 49].

Two of the 22 trials that compared psychological interventions to waitlist control or usual care of the interventions involved individual face-to-face sessions—both of these were effective [28, 30]. One [42] of the two studies [25, 42] that offered telephone support were effective at reducing fatigue. A combination in-person/telephone showed a reduction in fatigue at 3 months that was not maintained at 6 months [15]. Five of the studies reported on an online intervention [14, 26, 36, 43, 51]. The duration of these interventions varied from 6 weeks [14, 26, 36] to 6 months [51]. All of the interventions were stand-alone interventions and two reported a significant reduction in fatigue at final follow-up [36, 43, 51]

#### Comparison 5: interventions specifically designed to treat fatigue after cancer treatment vs. interventions not specific for fatigue

This review sought to interventions that were specifically designed to treat fatigue after cancer treatment and interventions not specific for fatigue. Nine of the 22 trials that compared psychological interventions to waitlist control or usual care were interventions specific for fatigue [16, 25, 26, 28, 33, 40, 43, 47, 49]*.* Of the nine studies on interventions specific for fatigue, five assessed fatigue as part of inclusion criteria ([26, 40, 43, 47, 49]. Only one of these six studies did not report a significant effect on fatigue [26]. Two of the four studies interventions specific for fatigue that did not assess fatigue as part of inclusion criteria were effective [28, 33]. Three studies were specific interventions for insomnia or sleep disturbance—all were effective for reducing fatigue [23, 36, 39]. The remaining studies aimed to address lifestyle and quality of life or physical activity. Of these, six studies were effective in reducing fatigue [15, 17, 22, 31, 42, 51] at least one follow-up point. However, the effect of the intervention on fatigue was not maintained in two of these studies at final follow-up [15, 17, 22, 31, 42, 51].

## Discussion

The aim of this review was to provide an overview of psychological interventions for fatigue after the completion of cancer treatment, and to evaluate the effectiveness of these interventions. In our search, 33 psychological interventions were identified, in which the effect on fatigue was tested in a RCT. The sample size of the included studies varied between 28 and 409, with 4525 participants overall. As with a previous review of interventions during treatment [8], the individual studies suggested that there is some evidence that psychological interventions are effective in reducing fatigue in cancer survivors. Twenty-three of the included studies reported a significant effect of the interventions on fatigue. However, the overall quality of the evidence about psychological interventions for fatigue after the completion of cancer treatment is low.

Given the heterogeneity in participant groups, study design, study comparators and measures used, we synthesised data narratively. Most interventions focused on psychoeducation, skills training, goal-setting, self-monitoring, problem-solving, identification of maladaptive cognitions and emotion-focused coping strategies. Interventions also integrated behaviour therapy-oriented strategies including stimulus control and other techniques, targeting physical activity, sleep and stress management. However, studies differed widely in terms of mode, duration and frequency of the intervention delivery. This has also been reported in other reviews of non-pharmacological interventions for fatigue [66]. There were also differences in the extent of contact across the different interventions. It was not possible to establish if certain types of intervention were superior for reducing fatigue or if there was potentially an influence of heterogeneous specific disease sites and cancer treatments. These issues have previously been reported in other studies [4, 11, 67].

Heterogeneity across the studies was also due to different definitions of fatigue criteria, various assessment tools and there were a number of different self-report measures used in the studies. As such, the same construct may not have been measured [68], as some tools were uni-dimensional, while others addressed the multi-dimensional nature of fatigue. Some of these measures were subscales of broader quality of life measures. Further, a number of these measures were designed specifically for cancer patients, while others were generic fatigue measures. Previous research has suggested that the lack of recommendations regarding fatigue measurement may be detrimental to research [68].

The strengths of this review includes the large number of studies included, a rigorous literature search based on a pre-published protocol; the use of independent raters; use of standard tools for reporting reviews and assessing bias in studies; and the presentation of a number of different variables that may be associated with intervention effectiveness. We are not aware of any studies that we have missed but acknowledge the potential for incomplete retrieval of identified research that may be a limitation of our review.

A number of limitations reduced our ability to make strong recommendations about any of the intervention strategies. In some studies, it was difficult to assess when exactly participants completed cancer treatment prior to participating in the study. As noted in similar reviews [68–70], the generalisability of the findings are limited due to the high proportion of studies that focused specifically on breast cancer or recruited a disproportionate number of breast cancer survivors. The majority of studies did not specifically target fatigue or screen for fatigue as part of inclusion criteria as recommended in existing guidelines [1, 6, 66]. Few studies described the cancer treatment received by participants in detail, such as types of treatments and total duration. In terms of trial design, most studies did not report on the adherence of participants to the intervention treatment, adverse effects or integrity checks that may allow further inferences to be made about the quality of the studies. Blinding of participants is often not possible to achieve in studies of this nature. However, as noted in other reviews of fatigue [67], it is troublesome that a number of studies did not ensure blinding of outcome assessment given the subjective and self-reported nature of the outcomes. Many aspects of trial procedures were not reported in sufficient detail to adequately assess risk of bias in all domains of all included trials. Trials with negative results might not have been published at all, and therefore may have been missed during our search.

## Conclusion

This review showed that there is some tentative support for psychological interventions for fatigue after cancer treatment based on the findings of individual studies. However, the RCTs were heterogeneous in nature and the number of high-quality studies was limited. Due to this heterogeneity, it is difficult to draw firm conclusions from the findings of this review. These findings demonstrate the need for the publication of more detailed descriptions of complex interventions, promoting methodological rigour and transparency in the design and throughout the trial process [71, 72]. Future trials need to consider the multidimensional nature of CrF in order to improve our understanding of this complex symptom [67].

## Supplementary information

**Additional file 1.** Summary of Findings for Secondary outcomes.

**Additional file 2.** Risk of Bias Assessment.

**Additional file 3.** Search strategies used in this review.

## Data Availability

The dataset supporting the conclusions of this article is included within the article (and its additional file(s)).

## Abbreviations

AMG: AnnMarie Groarke
ASCO: American Society of Clinical Oncology
BFI: Brief Fatigue Inventory
BMG: Brian E. McGuire
CIS: Checklist Individual Strength
CrF: Cancer-related fatigue
DD: Declan Devane
EC: Emma Carr
EORTC: European Organisation for Research and Treatment of Cancer
FACIT-F: Functional Assessment in Cancer Therapy – Fatigue
FACT: Functional Assessment of Cancer Therapy
FAQ: Fatigue Assessment Questionnaire
FSI: Fatigue Symptom Inventory
FSS: Fatigue Severity Scale
HADS: Hospital Anxiety and Depression Scale
IPOS: International Psycho-Oncology Society World Congress
ISI: Insomnia Severity Index
JW: Jane Walsh
MDSAI: M.D. Anderson Symptom Inventory
MeSH: Medical subject headings
MFI: Multidimensional Fatigue Inventory
MFSI-SF: Multidimensional Fatigue Symptom Inventory-Short Form
MOS: Medical outcomes study
NCCN: National Comprehensive Cancer Network
PHQ: Patient Health Questionnaire
PICO: Participants, Interventions, Comparisons, Outcome(s)
POMS: The Profile of Mood States
PRISMA: Preferred Reporting Items for Systematic Reviews and Meta-Analyses
PSQI: Pittsburgh Sleep Quality Index
QOL: Quality of life
RCT: Randomised controlled trial
SF-12: Medical Outcomes Study Short Form 12-Item Health Survey
SIP: Sickness Impact Profile
STAI: State-Trait Anxiety Inventory
TC: Teresa Corbett
VAS: Visual analogue scale
WHIIRS: Women’s Health Initiative Insomnia Rating Scale
WHO ICTRP: World Health Organization International Clinical Trials Registry Platform
