# Supplementary material for: The effectiveness of psychological interventions for fatigue in cancer survivors: systematic review of randomised controlled trials
Source: Syst Rev. 2019 Dec 13;8:324. doi: 10.1186/s13643-019-1230-2 (PMC6911282; doi:10.1186/s13643-019-1230-2)
Supplement: Supplementary file 1 — Additional file 1. Summary of Findings for Secondary outcomes. [file 13643_2019_1230_MOESM1_ESM.docx]

Additional file 1 Summary of Findings for Secondary outcomes.

| **Study ID** | **Outcome** | **Outcome Measure** | **Finding** |
| --- | --- | --- | --- |
| **Bantum 2014** | Mood | Patient Health Questionnaire (PHQ-8): depression | In the subgroup analyses looking at differences between survivors with diagnoses ≤ 2 and >2 years prior to enrollment, there were no significant differences, although there were suggested trends seen for depression (P=.09), such that people who were greater than 2 years post treatment improved slightly more on those measures (data not presented in paper).  Depression (PHQ)  Control group, mean (95% CI)  Baseline (n=176): 7.7 (7.0-8.3)  Month 6 (n=156): 7.1 (6.4-7.7)  Intervention group, mean (95% CI)  Baseline (n=176): 6.5 (5.9-7.1)  Month 6 (n=147): 6.1 (5.4-6.7)  *p*= 0 .69  Effect size Month 6= 0.19 (Calculated by taking the differences of the means at 6 months predicted from the model, including adjustment factors, divided by the standard deviation for the difference computed from the within and between subject variance components.) |
|  | Insomnia or sleep quality | Women’s Health Initiative Insomnia Rating Scale (WHIIRS) | Significant interactions between condition group and time were found for insomnia. The intervention group experienced an improvement from baseline to 6 months compared to the control group: reduced insomnia (9.6 to 9.2 compared to 9.6 to 10.1, P=.03). In the subgroup analyses looking at differences between survivors with diagnoses ≤ 2 and >2 years prior to enrollment, there were no significant differences, although there were suggested trends seen for insomnia (P=.07), such that people who were greater than 2 years post treatment improved slightly more on those measures (data not presented in paper).  Insomnia (WHIIRSe)  Control group, mean (95% CI)  Baseline (n=176): 9.6 (9.1-10.1)  Month 6 (n=156): 10.1 (9.6-10.7)  Intervention group, mean (95% CI)  Baseline (n=176): 9.6 (9.1-10.1)  Month 6 (n=147): 9.2 (8.7-9.8)  *p*=0 .03  Effect size Month 6= 0.20 (Calculated by taking the differences of the means at 6 months predicted from the model, including adjustment factors, divided by the standard deviation for the difference computed from the within and between subject variance components.) |
| **Bennett 2007** | Global quality of life / Functional impact of fatigue | [(SF-36, v.2) Physical Component Summary (PCS) and Mental Component Summary (MCS; Ware, 2005)](http://ovidsp.uk.ovid.com/sp-3.18.0b/ovidweb.cgi?QS2=434f4e1a73d37e8c3b61fcc465cfeaf23ab75d77d65257ba603f9d8fd3ead6782259710d76c3f201056b250a10399f8a05799aa3a01169bb69da301bc7ac19ff6278dc6dd2e8b6ce86e186ecd89a51502b5015f7d4bdd97c043744bc8bd86437fe8211a7940142fc1f401ce70b950fa1b403350d8a2198358aacd2116a81268981646b5f3dbd11d16cc9053d11a00f07e9987267e3fd74c053296e44fab620f6e667fbb423467904ccbea6cabf1ca40f78d26ff9301865f89ddaf6cb1accfd7fe0d2980606404c550eb10d1298cfc2a3b037bda74564eb114f136f84238938452169ab913b60c71deea74343dd5d46624031360cd890d3a75daf0dea2d6853733833aa95b449ae7a2c9dda596b1832a8233d2c8caf65f648f1dde78fb8260e955d8a77d8c8645515f112efa5ecbc143070b06b91e371052c846b7439af58268c21b6064e133134521d00edff524e7fcbe3e2a19918ae6cdd54ffd5319fe6a2c14932d55d701b283b0f244195e7169bf6e1e1831dc3949d75cbf73c9a17cdd968e404d197ce35421205dfe4a233a95750bc3cf738c441f8e5e6de3494cf9e2cfd#134) | Mental Component Summary  Results of the Level 1 MLM model showed that both the intercept (B = 45.65, p <.001) and the linear slope (B = 3.12, p <.01) were significantly different from zero. On average, the level of mental health status for all participants was 45.65 at baseline and increased 6 points (13%) across the study. There was significant individual variation in both the intercept and the slope to be explained in a Level 2 model; however, that model showed that group assignment was not associated significantly with variation around the mean slope.  Physical Component Summary (PCS)  Results of the Level 1 MLM model showed that both the intercept (B = 42.98, p <.001) and the linear slope (B = 1.57, p <.001) were significantly different from zero. On average, the level of physical health status for all participants was 42.98 at baseline and increased 3 points (7%) across the study. As there was no significant individual variation in the slope to be explained in a Level 2 model, an ANOVA analysis of group mean trajectory adequately represented the data. That analysis showed that the Group × Time interaction was not significant for physical health, [Wilk's lambda [LAMBDA] =.89, F(2,38) = 2.42, ns]. |
| **Blaes 2016** | Sleep | Pittsburgh Sleep quality index | At the 2-month assessment, sleep quality (PSQI, range 0-21, <5 = poorer sleep quality) in the MBCR group improved from the baseline 8.9 to 6.4, compared with the wait-list group (baseline 7.2 to 7.6); and at 4 months after course completion, it was 6.1 compared with 7.8, respectively (P = .03). |
|  | mood-anxiety | State Trait Anxiety ( STAI) | There was a trend toward improvement in the anxiety scores (STAI, range 20-80, higher score = greater anxiety) in the MBCR group compared with the wait-list group at 2 months (31.8 vs 39.4, respectively; P = .07) and 4 months (32.8 vs 40.7; P = .10). |
| **Bower 2015** | Fatigue self-efficacy | Fatigue subscale of the HIV self-efficacy questionnaire | Bower et al used the fatigue subscale of the HIV self-efficacy questionnaire and reported that Intervention group participants were significantly more confident than control group participants about their ability to manage fatigue and its impact on their lives at follow-up [1]. |
|  | Mood | Beck Depression Inventory-II (BDI-II) and Perceived Stress Scale (PSS) | MAPS intervention led to significant reductions in perceived stress (P = .004) and marginal reductions in depressive symptoms (P = .094)  Depressive symptoms: CES‐D  Baseline, n = 71  MAPS Group 14.50 ± 1.58 Control Group 19.25 ± 1.75  Postintervention, n = 65  MAPS Group 9.99 ± 1.64 Control Group 18.47 ± 1.80  *p*=0.095  3‐Month Follow‐Up, n = 59  MAPS Group 14.17 ± 1.70 Control Group 17.92 ± 1.82  *p*=0.664  PSS  Baseline, n = 71  MAPS Group 18.05 ± 0.99 Control Group 18.42 ± 1.12  Postintervention, n = 65  MAPS Group 14.25 ± 1.04 Control Group 19.15 ± 1.14  ***p*=0.004**  3‐Month Follow‐Up, n = 59  MAPS Group 17.42 ± 1.09 Control Group 18.21 ± 1.16  *p*=0.796 |
|  | Insomnia or sleep quality | Pittsburgh Sleep Quality Index (PSQI) | PSQI Baseline, n = 71  MAPS Group 8.13 ± 0.62 Control Group 8.39 ± 0.70  Postintervention, n = 65  MAPS Group 6.48 ± 0.65 Control Group 8.70 ± 0.71  ***p*=0.015**  3‐Month Follow‐Up, n = 59  MAPS Group 7.27 ±0.67 Control Group 7.86 ± 0.72  *p*=0.647 |
| **Bruggeman-Everts 2017** | Mental health | HADS & the Positive and Negative Affect Schedule | \| Outcome \| Condition \| Intercept at T0_b_(I) \| Linear slope factor (S) \| Two-tailed *P*value of linear slope (*P*) \| \| --- \| --- \| --- \| --- \| --- \| \| HADS \| AAF \| 13.237 (0.921) \| -0.076 (0.017) \| **<.001** \| \| eMBCT \| 13.903 (0.771) \| -0.110 (0.022) \| **<.001** \| \| Psycho-education \| 14.579 (1.012) \| -0.083 (0.024) \| **<.001** \| \| PA \| AAF \| 31.762 (0.939) \| 0.101 (0.022) \| **<.001** \| \| eMBCT \| 28.995 (0.932) \| 0.156 (0.026) \| **<.001** \| \| Psycho-education \| 29.422 (1.091) \| 0.128 (0.027) \| **<.001** \| \| NA \| AAF \| 20.330 (0.931) \| -0.068 (0.023) \| **0.003** \| \| eMBCT \| 20.718 (0.914) \| -0.071 (0.032) \| **0.03** \| \| Psycho-education \| 20.805 (1.215) \| -0.082 (0.029) \| **0.004** \| |
| **Carlson 2016** | Mood disturbance ( primary ) | POMS( anxiety, depression, anger, vigor, fatigue, confusion.) Calgary Symptoms of Stress Inventory | \| Outcomes \| Intervention \| \| \| \| \| Follow‐up \| \| \| \| \| --- \| --- \| --- \| --- \| --- \| --- \| --- \| --- \| --- \| --- \| \| Group × Time \| \| \| \| \| Group × Time \| \| \| \| \| Est \| se \| *t* \| *p* \| *d* [CI] \| Est \| se \| *t* \| *p* \| \| POMS \|  \|  \|  \|  \|  \|  \|  \|  \|  \| \| Anxiety \| −1.23 \| 0.46 \| −3.02 \| **0.003** \| **−0.39 [−0.64;−0.14]** \| 0.04 \| 0.08 \| 0.48 \| 0.63 \| \| Depression \| −1.58 \| 0.59 \| −2.72 \| **0.01** \| **−0.33 [−0.58;−0.08]** \| 0.10 \| 0.12 \| 0.86 \| 0.39 \| \| Anger \| −1.13 \| 0.44 \| −2.62 \| **0.01** \| **−0.35 [−0.60;−0.10]** \| 0.13 \| 0.13 \| 1.56 \| 0.12 \| \| Vigor \| 0.88 \| 0.41 \| 2.14 \| **0.03** \| **0.30 [0.05;0.55]** \| 0.01 \| 0.08 \| 0.14 \| 0.89 \| \| Fatigue \| −1.44 \| 0.42 \| −3.45 \| **0.001** \| **−0.45 [−0.70;−0.20]** \| −0.03 \| 0.08 \| −0.30 \| 0.76 \| \| Confusion \| −0.95 \| 0.30 \| −3.13 \| **0.0002** \| **−0.39 [−0.64;−0.14]** \| 0.02 \| 0.06 \| 0.26 \| 0.79 \| \| Total mood disturbance \| −6.29 \| 1.80 \| −3.49 \| **0.001** \| **−0.39 [−0.64;−0.14]** \| −0.06 \| 0.31 \| −0.19 \| 0.85 \| |
|  | QoL ( secondary ) | FACT-B Functional Assessment of Cancer Therapy - Breast module | \| FACT‐B \| Intervention \| \| \| \| \| Follow‐up \| \| \| \| \| --- \| --- \| --- \| --- \| --- \| --- \| --- \| --- \| --- \| --- \| \| Group × Time \| \| \| \| \| Group × Time \| \| \| \| \| Est \| se \| *t* \| *p* \| *d* [CI] \| Est \| se \| *t* \| *p* \| \|  \|  \|  \|  \|  \|  \|  \|  \|  \| \| Physical well‐being \| 0.51 \| 0.29 \| 1.73 \| **0.09** \| **0.22 [−0.03;0.47]** \| 0.02 \| 0.05 \| 0.41 \| 0.68 \| \| Social well‐being \| 0.43 \| 0.29 \| 1.49 \| 0.14 \| 0.17 [−0.08;0.42] \| −0.11 \| 0.06 \| −1.89 \| **0.06** \| \| Emotional well‐being \| 0.53 \| 0.25 \| 2.13 \| **0.03** \| **0.27 [0.02;0.52]** \| −0.02 \| 0.04 \| −0.51 \| 0.61 \| \| Functional well‐being \| 0.64 \| 0.28 \| 2.28 \| **0.02** \| **0.27 [0.02;0.52]** \| −0.03 \| 0.05 \| −0.65 \| 0.52 \| \| Breast cancer symptom scale \| 0.26 \| 0.32 \| 0.79 \| 0.43 \| 0.10 [−0.15;0.35] \| −0.04 \| 0.06 \| −0.74 \| 0.46 \| \| Total \| 2.00 \| 0.98 \| 2.03 \| **0.04** \| **0.22 [−0.03;0.47]** \| −0.14 \| 0.16 \| −0.86 \| 0.39 \| |
| **Dirksen 2008** | Global quality of life / Functional impact of fatigue | Functional Assessment of Cancer Therapy-Breast (FACT-B) (version 4) | \|  \| **Mean** \| **sd** \| **Mean** \| **sd** \| **Effect size** \| \| --- \| --- \| --- \| --- \| --- \| --- \| \| Functional Assessment of Cancer Therapy‐General \| \| \| \| \| \| \| CBT‐I \| 83·3 \| 11·9 \| 91·6 \| 15·0 \| 0·26 \| \| CC \| 84·8 \| 9·2 \| 87·7 \| 14·7 \| \| Functional Assessment of Cancer Therapy‐ Breast \| \| \| \| \| \| \| CBT‐I \| 108·5 \| 14·8 \| 118·8* \| 11·9 \| 0·37 \| \| CC \| 109·0 \| 20·0 \| 113·1* \| 18·2 \| \| FACT‐B physical well‐being \| \| \| \| \| \| \| CBT‐I \| 22·0 \| 5·6 \| 24·8* \| 3·3 \| 0·14 \| \| CC \| 23·1 \| 4·1 \| 24·3* \| 3·8 \| \| FACT‐B social well‐being \| \| \| \| \| \| \| CBT‐I \| 22·1 \| 4·9 \| 23·3 \| 3·9 \| 0·38 \| \| CC \| 22·2 \| 6·1 \| 21·4 \| 5·9 \| \| FACT‐B emotional well‐being \| \| \| \| \| \| \| CBT‐I \| 20·1 \| 2·8 \| 20·8 \| 2·3 \| 0·06 \| \| CC \| 20·4 \| 3·1 \| 20·6 \| 4·0 \| \| FACT‐B functional well‐being. \| \| \| \| \| \| \| CBT‐I \| 19·1 \| 4·0 \| 22·7* \| 4·2 \| 0·27 \| \| CC \| 19·1 \| 4·7 \| 21·5* \| 4·7 \| |
|  | Mood | • the State-Trait Anxiety Inventory (STAI) (state (STAI-S) and a trait anxiety scale (STAI-T)) • the Center for Epidemiologic Studies-Depression Scale (CES-D). | \|  \| **Mean** \| **sd** \| **Mean** \| **sd** \| **Effect size** \| \| --- \| --- \| --- \| --- \| --- \| --- \| \| State‐Trait Anxiety Inventory (state) \| \| \| \| \| \| \| \| CBT‐I \| 30·2 \| 8·7 \| 29·0 \| 8·8 \| 0·42 \| \| CC \| 31·8 \| 9·3 \| 33·7 \| 13·3 \| \| State‐Trait Anxiety Inventory (Trait) \| \| \| \| \| \| \| \| CBT‐I \| 36·5 \| 10·2 \| 32·9* \| 7·8 \| 0·24 \| \| CC \| 36·1 \| 9·3 \| 35·0 \| 9·4 \| \| Center for Epidemiologic Studies‐Depression Scale \| \| \| \| \| \| \| \| CBT‐I \| 11·6 \| 7·3 \| 7·8* \| 7·3 \| 0·15 \| \| CC \| 10·9 \| 7·8 \| 9·1 \| 9·7 \| |
| **Dodds 2015** | Mood  Pain | Five subscales of the Fear of Cancer Recurrence Inventory (FCRI)  Impact of Events Scale—Revised (IES-R)  Revised UCLA Loneliness Scale Version 3 (R-UCLA)  Brief Center for Epidemiologic Studies—Depression questionnaire (CES-D-10)  Medical Outcomes Study Short Form 12-Item Health Survey (SF-12) | \| Outcome \| Intervention–control (95 % CI) \| \| \| \| --- \| --- \| --- \| --- \| \|  \| 1-month FU (*N* = 11) \| Post \| 1-month FU \| \| Perceived stress \| **5.1 (3.0)** \| **−1.2 (−2.5, 0.2)** \| **−1.6 (−3.1, −0.2)*** \| \| Depression \| 5.5 (5.0) \| −3.7 (−6.3, −1.1)** \| −1.3 (−4.2, 1.6) \| \| FCR: triggers \| 12.5 (5.8) \| −2.2 (−6.0, 1.6) \| 1.7 (−2.4, 5.8) \| \| FCR: severity \| 13.7 (8.5) \| −0.9 (−2.9, 1.2) \| 0.6 (−1.7, 2.8) \| \| FCR: psychological distress \| 3.3 (4.5) \| −0.1 (−1.5 1.3) \| 0.4 (−1.2, 2.0) \| \| FCR: functioning impairments \| 1.7 (2.7) \| −1.3 (−2.5–0.1)* \| 1.3 (−0.1, 2.7) \| \| FCR: insight \| 1.1 (2.1) \| −0.3 (−0.8, 0.2) \| −0.3 (−0.9, 0.3) \| \| Traumatic stress: intrusion \| 0.5 (0.3) \| −0.1 (−0.3, 0.2) \| −0.1 (−0.3, 0.2) \| \| Traumatic stress: avoidance \| 0.7 (0.8) \| −0.3 (−0.6, −0.02)* \| 0.1 (−0.2, 0.4) \| \| Traumatic stress: hyperarousal \| 0.4 (0.4) \| −0.1 (−0.3, 0.2) \| −0.003 (−0.3, 0.3) \| \| Traumatic stress: global \| 1.6 (1.3) \| −0.4 (−1.0, 0.2) \| 0.04 (−0.6, 0.7) \| \| Loneliness \| 37.9 (16.6) \| −2.9 (−7.7, 2.0) \| −2.5 (−7.9, 3.0) \| \| Bodily pain \| 52.0 (7.0) \| 2.0 (−3.1, 7.0) \| −1.9 (−7.5, 3.8) \| \| Physical well-being \| **54.0 (4.9)** \| **−0.1 (−3.2, 2.9)** \| **−4.3 (−7.7, −0.9)*** \| \| Mental well-being \| 46.5 (10.4) \| 2.0 (−2.4, 6.5) \| 4.4 (−0.6, 9.3) \| |
| **Dolbeault 2009** | Global quality of life / Functional impact of fatigue  Mood  Insomnia or sleep quality  Pain | EORTC QLQ-C30 and breast cancer module (EORTC QLQ-BR23).  State-Trait Anxiety Inventory Profile of Mood States (POMS)  The Mental Adjustment to Cancer Scale (MAC) EORTC QLQ-C30 sleep  EORTC pain | In both groups changes were observed over time in the STAI trait and state anxiety scores, the POMS anxiety, anger, confusion, depression and global scores, the MAC helplessness–hopelessness and anxious preoccupations scores, in the EORTC QLQ-C30 scores for physical, emotional, cognitive and social functioning, dyspnoea, sleep and financial difficulties, and in the QLQ-BR23 body image, future prospects and breast symptom scores. Controlling for a time effect, significant group/ time interactions indicate a positive effect of the intervention on anxiety, our primary outcome measure. This was evidenced for the STAI state and trait anxiety scales, explaining 6% and 4% of the variance in the STAI-state and STAI-trait anxiety scores, respectively  Found positive results on other variables identified as secondary outcome measures. A greater reduction of negative affect and improvement in positive affect and in quality of life functional or symptom scales were observed in the TG compared with the CG. This concerned the POMS anxiety and global scores (8% of the variance explained by the model including the time/group interaction term), scores for fatigue (7%), anger (5%), interpersonal relationships (4%), vigor (3%) and depression (2%) and the EORTC QLQ-C30 scores, emotional functioning (9%), role functioning (3%), global health status (3%) and fatigue (3%). In contrast, no effect of the PEG was evidenced on the MAC scale or on the POMS confusion scores. |
| **Espie 2008** | Global quality of life / Functional impact of fatigue | Functional Assessment of Cancer Therapy Scale– general FACT-G | CBT participants had increased physical and functional QOL relative to TAU. Correlations between changes in SE from baseline to post-treatment after CBT and changes in statistically significant QOL measures were low.  **FACT** **Physical**  Post-Treatment Standardized Effect= 0.58  Post-Treatment 95% CI= - 0.19 to 0.97  Post-Treatment *p*=0.004*  6-Month Follow-Up Standardized Effect= 0.74  6-Month Follow-Up 95% CI=- 0.34 to 1.14  6-Month Follow-Up *p* <0 .001†  **FACT** **Social**  Post-Treatment Standardized Effect= 0.42  Post-Treatment 95% CI= 0.03 to 0.81  Post-Treatment *p*=0.036  6-Month Follow-Up Standardized Effect= 0.13  6-Month Follow-Up 95% CI= -0.27 to 0.53  6-Month Follow-Up *p*=0.529  **FACT** **Emotional**  Post-Treatment Standardized Effect= 0.38  Post-Treatment 95% CI= -0.01 to 0.78  Post-Treatment *p*=0.057  6-Month Follow-Up Standardized Effect= 0.16  6-Month Follow-Up 95% CI= - 0.25 to 0.57  6-Month Follow-Up *p=*0. 444  **FACT** **Functional**  Post-Treatment Standardized Effect= 0.86  Post-Treatment 95% CI= 0.47 to 1.25  Post-Treatment *p*<0 .001†  6-Month Follow-Up Standardized Effect= 1.17  6-Month Follow-Up 95% CI= 0.77 to 1.57  6-Month Follow-Up *p*<0 .001†  *Significant at 5% after adjustment for multiple comparisons within each time point using the Hochberg procedure. †Significant at 1% after adjustment for multiple comparisons within each time point using the Hochberg procedure. |
|  | Mood | Hospitals Anxiety and Depression Scale [HADS] | CBT participants had reduced symptoms of anxiety, and depression relative to TAU. Correlations between changes in SE from baseline to post-treatment after CBT and changes in statistically significant QOL measures were low.  HADS  **Anxiety**  Post-Treatment Standardized Effect= -0.57  Post-Treatment 95% CI= -0.96 to -0.18  Post-Treatment *p*=0.005*  6-Month Follow-Up Standardized Effect= -0.52  6-Month Follow-Up 95% CI=-0.92 to-0.12  6-Month Follow-Up *p*=0.011*  **Depression**  Post-Treatment Standardized Effect= -0.67  Post-Treatment 95% CI= -1.06 to -0.28  Post-Treatment *p*=0.001†  6-Month Follow-Up Standardized Effect= -0.59  6-Month Follow-Up 95% CI= -0.99 to -0.19  6-Month Follow-Up *p*=0.004*  *Significant at 5% after adjustment for multiple comparisons within each time point using the Hochberg procedure. †Significant at 1% after adjustment for multiple comparisons within each time point using the Hochberg procedure. |
|  | Insomnia or sleep quality | PSQI, Epworth sleepiness (baseline only) and sleep diary assessed the central insomnia dimensions of difficulty initiating (SOL) and maintaining (WASO) sleep. | At post-treatment, CBT was associated with median reduction in SOL of 16 minutes (95% CI, 10 to 22 minutes), and in WASO of3 8 minutes (95% CI, 28 to 59 minutes), the corresponding median reductions following TAU were 0 minutes (95% CI, -8.5 to 6.6) and 2 minutes (95% CI, -15 to 9). Effect sizes were moderate to large and were both highly statistically significant (*p*= 0.001). TST also increased by a median of 16 minutes (95% CI, -1 to 30) with CBT compared with 5 minutes (95% CI, -14 to 24) after TAU, but the difference between arms was not statistically significant. SE increased by 10% (95% CI, 9% to 12%) after CBT; the change in the TAU was 0% (95% CI, -3% to 3%). This effect size was large and highly statistically significant. This pattern of results generally held at 6 months post-treatment. Effect sizes were somewhat reduced for WASO, SOL, and SE but remained moderate and statistically significant (*p*=0.001). Changes in TST again were not statistically significant. In summary, CBT was associated with median reduction in insomnia symptoms of almost 1 hour (SOL + WASO) compared with no change following TAU. Post-treatment and follow-up SE of 85% is commonly regarded as the lower limit of normal sleep. A higher proportion of CBT participants achieved this criterion, 51% (51 of 100) versus 34% on TAU (17 of 50; *p*=0.008); at 6 months this difference was no longer significant (44%; 44/100 of patients on CBT; 48%; 24 of 50 on TAU; *p*= 0 .966). |
| **Ferguson 2016** | QoL/self-reported function | FACTCog Impact on Quality of Life scale | On the FACT‐Cog Impact on Quality of Life scale, MAAT and ST participants did not differ at the posttreatment (*F* (1,28), 0.187; *P* = .67) or 2‐month follow‐up (*F* (1,28), 1.19; *P* = .28) time points, but a moderate effect size was observed at the 2‐month follow‐up, with MAAT participants reporting higher QOL (*d* = 0.43). On other QOL measures, MAAT and ST participants did not differ with regard to general function at either the posttreatment (*F* (1,28), 0.236; *P* = .63) or 2‐month follow‐up (*F* (1,28), 1.14; *P* = .295) time points.  However, the Cohen's *d* effect sizes for function, at the 2‐month follow‐up time point suggested that MAAT participants demonstrated sustained clinical gains on this QOL measures compared with ST participants (general function, 0.50). |
|  | general anxiety and depression | Depression Anxiety Stress Scales-21 [DASS-21] | With respect to anxiety about cognitive problems in daily life (MIA‐A), the MAAT and ST participants did not differ at the posttreatment follow‐up time point (*F*(1,28), 0.089; *P* = .77). However, at 2‐month follow‐up, there was a trend toward MAAT participants having decreased anxiety regarding cognitive problems (*F*(1,28), 3.53; *P* = .07), with a large effect size noted (*d* = .90)  However, the Cohen's *d* effect sizes for anxiety at the 2‐month follow‐up time point suggested that MAAT participants demonstrated sustained clinical gains on this measures compared with ST participants (DASS‐21 anxiety scale, 0.55).  MAAT and ST participants did differ at a statistically significant level with regard to depression at baseline. This suggests that ST participants were more depressed and thus could have had more cognitive problems affecting results. |
| **Fillion 2008** | Global quality of life / Functional impact of fatigue | Medical Outcomes Study Short Form 12  Menopause-Specific Quality of Life Questionnaire | Marginal Group × Time interaction effects (ANCOVA) emerged for physical quality of life, and significant Group and Time main effects were obtained for physical quality of life.  Simple effect contrasts revealed a significant Group difference at T1 for physical quality of life. That is, women who received the intervention showed a significantly higher level of physical quality of life immediately after the intervention (T1) compared with women in the control group. The same analyses conducted on mental quality of life showed no interaction or main effects, thus demonstrating that both conditions improved in a similar manner on mental quality of life overtime (P > .05). However, an ad hoc simple effect contrast revealed a significant effect at follow-up, F1,83 = 4.37, P = .04 indicating that the experimental group's mental quality of life improvement was more important than that of the control group. |
|  | Mood | Profile of Mood States: combined anxiety and depression subscales | A reduction in emotional distress (ie, combined Profile of Mood States depression/anxiety items) was predicted both immediately after the intervention and at follow-up. A mixed-model ANCOVA (adjusting for physical menopausal symptoms) on emotional distress was conducted. No interaction or Time main effects for emotional distress emerged, meaning that, overall, the participants' level of distress did not change over time. However, a Group main effect was revealed. When examining pairwise comparisons, emotional distress significantly differed at follow-up (Control M = 13.13, SD = 5.44; Experimental M = 11.15, SD = 3.85), thus revealing that the participants exposed to the intervention experienced less distress (ie, less combined depression and anxiety symptoms) at 3-month follow-up compared with those in the control condition. |
|  | Pain | Brief Pain Inventory | Not reported |
| **Foster 2015** | Global quality of life / Functional impact of fatigue | Functional Assessment of Cancer Therapy—General (FACT-G) and Personal Wellbeing Index (PWI) | \|  \| **Time point** \| **Mean (SD)** \| \| **Group effect (95 % CI)** \| ***P*** \| \| --- \| --- \| --- \| --- \| --- \| --- \| \| **RESTORE** \| **Comparator** \| \| Personal Wellbeing Index (range 0–100)^a^ \| T0 \| 64.9 (17.2) \| 63.0 (19.8) \| – \| – \| \| T1 \| 65.3 (19.1) \| 64.6 (18.6) \| 0.622 (−3.437, 4.682) \| 0.76 \| \| T2 \| 63.8 (21.8) \| 65.1 (24.1) \| 0.244 (−5.687, 6.175) \| 0.94 \| \| FACT-G (range 0–108)^a^ \| T0 \| 72.9 (16.2) \| 71.4 (17.8) \| – \| – \| \| T1 \| 74.1 (18.0) \| 76.9 (17.4) \| −2.206 (−5.503, 1.091) \| 0.19 \| \| T2 \| 75.0 (19.4) \| 78.7 (18.5) \| −3.034 (−6.639, 0.571) \| 0.10 \| |
|  | Fatigue self-efficacy | Perceived Self-efficacy for Fatigue Self-management (PSEFSM) | There is evidence of improved fatigue self-efficacy at T1 (0.514, 95 % CI [−0.084, 1.112], P = 0.09), in the RESTORE group though the impact is lost by T2   \|  \| **Time point** \| **Mean (SD)** \| \| **Group effect (95 % CI)** \| ***P*** \| \| --- \| --- \| --- \| --- \| --- \| --- \| \| **RESTORE** \| **Comparator** \| \| Fatigue self-efficacy (range 1–11) \| T0 \| 5.376 (1.930) \| 5.373 (2.048) \| – \| – \| \| T1 \| 6.421 (1.781) \| 5.904 (2.107) \| 0.514 (−0.084, 1.112) \| 0.09 \| \| T2 \| 6.439 (2.228) \| 6.294 (2.207) \| 0.106 (−0.427, 0.638) \| 0.70 \| |
|  | Mood | Patient Health Questionnaire (PHQ-9) | \|  \| **Time point** \| **Mean (SD)** \| \| **Group effect (95 % CI)** \| ***P*** \| \| --- \| --- \| --- \| --- \| --- \| --- \| \| **RESTORE** \| **Comparator** \| \| PHQ-9 (range 0–27) \| T0 \| 9.77 (5.50) \| 8.96 (5.66) \| – \| – \| \| T1 \| 8.41 (5.58) \| 7.74 (5.82) \| −0.452 (−1.761, 0.858) \| 0.50 \| \| T2 \| 8.59 (6.37) \| 6.82 (5.50) \| 0.676 (−0.880, 2.231) \| 0.40 \| |
| **Freeman 2015** | Global quality of life / Functional impact of fatigue | Medical Outcomes Study 36-item short form survey (SF-36)  FACT-B | \|  \| **Live Delivery n = 48** \| \| **Telemedicine Delivery n = 23** \| \| **Waitlist Control n = 47** \| \| **Group Effect** \| **Time Effect** \| **Group**[**^*^**](https://www.ncbi.nlm.nih.gov/pmc/articles/PMC4379121/table/T2/?report=objectonly#TFN2)**Time Effect** \| \| --- \| --- \| --- \| --- \| --- \| --- \| --- \| --- \| --- \| --- \| \|  \| \| \| \| \| \| \| \| \|  \| **M** \| **SD** \| **M** \| **SD** \| **M** \| **SD** \| ***p*-value** \| ***p*-value** \| ***p*-value** \| \| SF-36 PCS \|  \|  \|  \|  \|  \|  \| 0.154 \| 0.529 \| 0.111 \| \| Baseline \| 47.20 \| 8.60 \| 46.54 \| 8.48 \| 45.24 \| 10.23 \|  \|  \|  \| \| 1 Month \| 48.81 \| 9.84 \| 48.64 \| 9.05 \| 43.49 \| 11.34 \|  \|  \|  \| \| 3 Months \| 50.54 \| 8.49 \| 46.95 \| 8.04 \| 45.44 \| 10.24 \|  \|  \|  \| \| Group LSM, SE[^*^](https://www.ncbi.nlm.nih.gov/pmc/articles/PMC4379121/table/T2/?report=objectonly#TFN2) \| 48.32 \| 0.91 \| 49.93 \| 1.36 \| 46.81 \| 0.91 \|  \|  \|  \| \|  \| \| \| \| \| \| \| \| \| SF-36 MCS \|  \|  \|  \|  \|  \|  \| 0.020 \| 0.612 \| 0.661 \| \| Baseline \| 42.45 \| 10.50 \| 43.45 \| 8.03 \| 42.41 \| 10.04 \|  \|  \|  \| \| 1 Month \| 48.51 \| 8.72 \| 49.25 \| 7.97 \| 46.50 \| 10.40 \|  \|  \|  \| \| 3 Months \| 49.80 \| 8.04 \| 50.84 \| 7.58 \| 43.29 \| 12.75 \|  \|  \|  \| \| Group LSM, SE[^*^](https://www.ncbi.nlm.nih.gov/pmc/articles/PMC4379121/table/T2/?report=objectonly#TFN2) \| 48.77 \| 1.24 \| 49.40 \| 1.86 \| 44.30 \| 1.25 \|  \|  \|  \| \|  \| \| \| \| \| \| \| \| \| FACT-B \|  \|  \|  \|  \|  \|  \| 0.076 \| 0.003 \| 0.208 \| \| Baseline \| 22.63 \| 5.98 \| 22.09 \| 4.03 \| 20.32 \| 6.06 \|  \|  \|  \| \| 1 Month \| 25.32 \| 5.97 \| 24.84 \| 5.29 \| 22.32 \| 6.08 \|  \|  \|  \| \| 3 Months \| 26.18 \| 5.83 \| 27.21 \| 4.22 \| 22.72 \| 5.20 \|  \|  \|  \| \| Group LSM, SE[^*^](https://www.ncbi.nlm.nih.gov/pmc/articles/PMC4379121/table/T2/?report=objectonly#TFN2) \| 24.66 \| 0.57 \| 26.03 \| 0.85 \| 23.66 \| 0.58 \|  \|  \|  \| \| There was no group effect on PCS, MCS, or FACT-B, though means were in the expected direction. There were no group*time effects that reached the adjusted alpha level of 0.011 \| \| \| \| \| \| \| \| |
|  | Mood | Psychological distress : Brief Symptom Inventory-Global Severity Index (BSI-GSI) | \|  \| **Live Delivery n = 48** \| \| **Telemedicine Delivery n = 23** \| \| **Waitlist Control n = 47** \| \| **Group Effect** \| **Time Effect** \| **Group**[**^*^**](https://www.ncbi.nlm.nih.gov/pmc/articles/PMC4379121/table/T2/?report=objectonly#TFN2)**Time Effect** \| \| --- \| --- \| --- \| --- \| --- \| --- \| --- \| --- \| --- \| --- \| \| BSI-GSI \|  \|  \|  \|  \|  \|  \| 0.051 \| 0.120 \| 0.032 \| \| Baseline \| 53.98 \| 7.75 \| 51.77 \| 7.81 \| 55.51 \| 7.26 \|  \|  \|  \| \| 1 Month \| 48.88 \| 8.31 \| 49.32 \| 8.58 \| 52.20 \| 8.44 \|  \|  \|  \| \| 3 Months \| 46.80 \| 7.82 \| 49.26 \| 7.34 \| 53.02 \| 8.95 \|  \|  \|  \| \| Group LSM, SE[^*^](https://www.ncbi.nlm.nih.gov/pmc/articles/PMC4379121/table/T2/?report=objectonly#TFN2) \| 48.24 \| 1.02 \| 47.81 \| 1.59 \| 51.51 \| 1.03 \|  \|  \|  \| |
|  |  |  | There was no group effect on BSIGSI though means were in the expected direction.  Though there were no group*time effects that reached the adjusted alpha level of 0.011, there was a group*time effect on BSIGSI scores at the p < 0.05 level (p = 0.032). Pairwise comparisons of groups at each time point revealed that neither TD or LD differed from WL at the 1-month follow-up (p’s > 0.3), both LD (p = 0.011) and TD (p = 0.004) reported lower BSIGSI than WL at the 3-month follow-up, and TD and LD did not differ from one another at either time point (p’s > 0.7). |
|  | Insomnia or sleep quality | Pittsburgh Sleep Quality Index (PSQI) | \|  \| **Live Delivery n = 48** \| \| **Telemedicine Delivery n = 23** \| \| **Waitlist Control n = 47** \| \| **Group Effect** \| **Time Effect** \| **Group**[**^*^**](https://www.ncbi.nlm.nih.gov/pmc/articles/PMC4379121/table/T2/?report=objectonly#TFN2)**Time Effect** \| \| --- \| --- \| --- \| --- \| --- \| --- \| --- \| --- \| --- \| --- \| \| PSQI \|  \|  \|  \|  \|  \|  \| **<0.001** \| 0.346 \| 0.303 \| \| Baseline \| 8.79 \| 4.11 \| 8.30 \| 3.74 \| 9.96 \| 4.74 \|  \|  \|  \| \| 1 Month \| 6.12 \| 3.74 \| 5.95 \| 3.47 \| 9.18 \| 4.61 \|  \|  \|  \| \| 3 Months \| 6.70 \| 3.83 \| 5.53 \| 2.46 \| 9.74 \| 4.32 \|  \|  \|  \| \| Group LSM, SE[^*^](https://www.ncbi.nlm.nih.gov/pmc/articles/PMC4379121/table/T2/?report=objectonly#TFN2) \| 7.09 \| 0.36 \| 6.04 \| 0.54 \| 8.74 \| 0.37 \|  \|  \|  \| |
|  |  |  | Using a Bonferroni correction for multiple QOL comparisons (alpha = 0.011), there was an effect of group on PSQI (p’s ≤ 0.002). There were no group*time effects that reached the adjusted alpha level of 0.011. |
| **Gielissen 2006** | Global quality of life / Functional impact of fatigue | Sickness Impact Profile-8 (SIP-8). | The proportion of patients with clinically significant improvement on functional impairment was significantly higher in the intervention condition than in the waiting list condition. Patients in the intervention condition reported a significantly greater decrease in functional impairment (difference, 383.2; 95% CI, 197.1 to 569.2) than patients in the waiting list condition. |
|  | Mood | Psychological distress was measured by the Symptom Check List 90 | Patients in the intervention condition reported a significantly greater decrease in psychological distress (difference, 21.6; 95% CI, 12.7 to 30.4) than patients in the waiting list condition. |
| **Heckler 2016** | No secondary outcomes reported | |  |
| **Hoffman 2012** | Global quality of life / Functional impact of fatigue | Functional Assessment of Cancer Therapy-Breast (FACT-B) FACT, Functional Assessment of Cancer Therapy   Functional Assessment of Cancer Therapy-Endocrine Symptoms (FACT-ES)   WHO five-item well-being questionnaire (WHO-5) | After adjustment for the outcome measurement made at T1, there were statistically significant treatment effects for FACT-ES, FACT-B, physical well-being, social well-being, emotional wellbeing, and functional well-being. Mean scores in the experimental group compared with the control group were greater at both T2 and T3 for all six measures (except social well-being which was significant at T2 only). For emotional well-being, there was some evidence that treatment effects at T3 were statistically significantly greater that at T2. No other interactions were statistically significant.  After adjustment for T1 measurements, there were statistically significant increases in the WHO-5 in the experimental group compared with controls, and these were apparent at T2 and T3.  For the WHO-5, the minimum clinically important difference has been suggested to be a change of 10% on standardized percentage scores, which are obtained by multiplying the raw scores by four. The adjusted mean differences, expressed as standardized percentage scores, were 8.04% at T2 and 8.60% at T3. These scores were close to the minimum clinically important difference of 10%   \| **Outcome Measure** \| **Experimental Group (n = 103)** \| \| \| \| \| **Control Group (n = 111)** \| \| \| \| \| **Difference Between Groups at T2 and T3 Adjusted for Baseline** \| \| \| \| --- \| --- \| --- \| --- \| --- \| --- \| --- \| --- \| --- \| --- \| --- \| --- \| --- \| --- \| \| **N** \| \| **Mean** \| \| **SD** \| **N** \| **Mean** \| \| \| **SD** \| **Mean** \| \| **95% CI** \| \| FACT-ES \|  \| \|  \| \|  \|  \|  \| \| \|  \|  \| \|  \| \| T1 \| 102 \| \| 127.02 \| \| 18.84 \| 107 \| 127.08 \| \| \| 23.20 \| NA \| \|  \| \| T2 \| 102 \| \| 134.97 \| \| 19.26 \| 107 \| 127.37 \| \| \| 23.58 \| 7.65 \| \| 3.95 to 11.36 \| \| T3 \| 102 \| \| 135.34 \| \| 19.54 \| 107 \| 127.42 \| \| \| 21.26 \| 7.98 \| \| 4.46 to 11.49 \| \| Interaction time × treatment group, *P* \| \| \| \| \| \| \| \| \| \| \| .814 \| \| \| \| Treatment group main effect, *P* \| \| \| \| \| \| \| \| \| \| \| **< .001** \| \| \| \| FACT-B \|  \| \|  \| \|  \|  \|  \| \| \|  \|  \| \|  \| \| T1 \| 101 \| \| 96.57 \| \| 17.22 \| 106 \| 96.68 \| \| \| 21.05 \| NA \| \| \| \| T2 \| 101 \| \| 103.56 \| \| 17.91 \| 106 \| 96.84 \| \| \| 21.14 \| 6.81 \| \| 3.48 to 10.14 \| \| T3 \| 101 \| \| 103.78 \| \| 17.85 \| 106 \| 96.22 \| \| \| 19.43 \| 7.65 \| \| 4.61 to 10.68 \| \| Interaction time × treatment group, *P* \| \| \| \| \| \| \| \| \| \| \| .493 \| \| \| \| Treatment group main effect, *P* \| \| \| \| \| \| \| \| \| \| \| **< .001** \| \| \| \| FACT PWB \|  \| \|  \| \|  \|  \|  \| \| \|  \|  \| \|  \| \| T1 \| 102 \| \| 21.88 \| \| 4.29 \| 111 \| 21.89 \| \| \| 4.35 \| NA \| \| \| \| T2 \| 102 \| \| 22.86 \| \| 4.22 \| 111 \| 21.84 \| \| \| 4.54 \| 1.03 \| \| 0.19 to 1.87 \| \| T3 \| 102 \| \| 22.97 \| \| 4.34 \| 111 \| 21.67 \| \| \| 4.87 \| 1.31 \| \| 0.49 to 2.12 \| \| Interaction time × treatment group, *P* \| \| \| \| \| \| \| \| \| \| \| .521 \| \| \| \| Treatment group main effect, *P* \| \| \| \| \| \| \| \| \| \| \| **.002** \| \| \| \| FACT SWB \|  \| \|  \| \|  \|  \|  \| \| \|  \|  \| \|  \| \| T1 \| 102 \| \| 17.59 \| \| 5.91 \| 109 \| 18.78 \| \| \| 6.01 \| NA \| \| \| \| T2 \| 102 \| \| 18.36 \| \| 5.65 \| 109 \| 18.26 \| \| \| 5.88 \| 1.06 \| \| 0.17 to 1.94 \| \| T3 \| 102 \| \| 18.09 \| \| 5.81 \| 109 \| 18.30 \| \| \| 5.75 \| 0.71 \| \| −0.24 to 1.65 \| \| Interaction time × treatment group, *P* \| \| \| \| \| \| \| \| \| \| \|  \| \| .436 \| \| Treatment group main effect, *P* \| \| \| \| \| \| \| \| \| \| \|  \| \| .032 \| \| FACT EWB \| \|  \| \|  \|  \|  \| \|  \|  \| \|  \|  \| \| \| T1 \| \| 102 \| \| 16.91 \| 3.84 \| 109 \| \| 15.97 \| 4.58 \| \| NA \| \| \| \| T2 \| \| 102 \| \| 18.14 \| 3.82 \| 109 \| \| 16.59 \| 4.40 \| \| 0.93 \| 0.09 to 1.78 \| \| \| T3 \| \| 102 \| \| 18.59 \| 3.75 \| 109 \| \| 16.28 \| 4.42 \| \| 1.72 \| 0.86 to 2.57 \| \| \| Interaction time × treatment group, *P* \| \| \| \| \| \| \| \| \| \| **.042** \| \| \| \| \| Treatment group main effect, *P* \| \| \| \| \| \| \| \| \| \| **.001** \| \| \| \| \| FACT FWB \| \|  \| \|  \|  \|  \| \|  \|  \| \|  \|  \| \| \| T1 \| \| 102 \| \| 17.83 \| 5.03 \| 110 \| \| 17.65 \| 5.83 \| \| NA \| \| \| \| T2 \| \| 102 \| \| 19.46 \| 5.27 \| 110 \| \| 17.41 \| 6.06 \| \| 1.91 \| 0.87 to 2.95 \| \| \| T3 \| \| 102 \| \| 19.45 \| 5.32 \| 110 \| \| 17.53 \| 5.37 \| \| 1.80 \| 0.77 to 2.83 \| \| \| Interaction time × treatment group, *P* \| \| \| \| \| \| \| \| \| \| .804 \| \| \| \| \| Treatment group main effect, *P* \| \| \| \| \| \| \| \| \| \| < .001 \| \| \| \| \| WHO-5 \| \|  \| \|  \|  \|  \| \|  \|  \| \|  \|  \| \| \| T1 \| \| 103 \| \| 13.04 \| 4.48 \| 111 \| \| 12.53 \| 4.68 \| \| NA \| \| \| \| T2 \| \| 103 \| \| 14.91 \| 4.23 \| 111 \| \| 12.60 \| 4.92 \| \| 2.01 \| 1.00 to 3.01 \| \| \| T3 \| \| 103 \| \| 15.08 \| 4.62 \| 111 \| \| 12.65 \| 4.30 \| \| 2.15 \| 1.16 to 3.15 \| \| \| Interaction time × treatment group, *P* \| \| \| \| \| \| \| \| \| \| \| .768 \| \| \| \| Treatment group main effect, *P* \| \| \| \| \| \| \| \| \| \| \| **< .001** \| \| \| |
|  | Mood | POMS | There were statistically significant differences between treatment groups for POMS total mood disturbance, anxiety, depression, anger, vigor, fatigue, and confusion. The T1-adjusted mean differences and 95% CIs at T2 and T3 suggested statistically significant lowermood-state scores in the experimental group than in the control group at both measurement occasions except for depression (T2 only), anger (T3 only), and confusion (T2 only). There were no statistically significant interactions between treatment group and measurement occasion.   \| Outcome Measure \| Experimental (n = 103) \| \| Control (n = 111) \| \| Difference Between Groups at T2 and T3 Adjusted for Baseline \| \| \| --- \| --- \| --- \| --- \| --- \| --- \| --- \| \| Mean \| SD \| Mean \| SD \| Mean \| 95% CI \| \| Total score \|  \|  \|  \|  \|  \|  \| \| T1 total mood disturbance \| 43.65 \| 34.73 \| 49.23 \| 39.37 \| NA \| \| \| T2 total mood disturbance \| 30.02 \| 31.60 \| 48.08 \| 39.89 \| −15.30 \| −23.75 to −6.86 \| \| T3 total mood disturbance \| 29.83 \| 34.19 \| 45.47 \| 35.67 \| −12.91 \| −21.02 to −4.81 \| \| Interaction time × treatment group, *P* \| \| \| \| \| 558 \| \| \| Treatment group main effect, *P* \| \| \| \| \| **< .001** \| \| \| Subscales \|  \|  \|  \|  \|  \|  \| \| T1 tension/anxiety \| 13.16 \| 7.20 \| 13.42 \| 7.24 \| NA \| \| \| T2 tension/anxiety \| 10.32 \| 7.0 \| 13.36 \| 7.20 \| −2.93 \| −4.67 to −1.20 \| \| T3 tension/anxiety \| 10.33 \| 7.02 \| 12.73 \| 6.59 \| −2.30 \| −3.96 to −0.63 \| \| Interaction time × treatment group, *P* \| \| \| \| \| .493 \| \| \| Treatment group main effect, *P* \| \| \| \| \| **< .001** \| \| \| T1 depression/dejection \| 12.79 \| 10.76 \| 15.70 \| 12.79 \| NA \| \| \| T2 depression/dejection \| 10.0 \| 9.95 \| 14.96 \| 13.23 \| −3.39 \| −6.06 to −0.71 \| \| T3 depression/dejection \| 10.34 \| 10.32 \| 14.10 \| 11.60 \| −2.32 \| −4.86 to 0.22 \| \| Interaction time × treatment group, *P* \| \| \| \| \| .365 \| \| \| Treatment group main effect, *P* \| \| \| \| \| **.017** \| \| \| T1 anger/hostility \| 10.75 \| 8.08 \| 11.60 \| 8.62 \| NA \| \| \| T2 anger/hostility \| 8.78 \| 7.57 \| 11.11 \| 8.88 \| −1.96 \| −3.96 to 0.05 \| \| T3 anger/hostility \| 7.87 \| 6.72 \| 11.04 \| 8.95 \| −2.69 \| −4.44 to −0.95 \| \| Interaction time × treatment group, *P* \| \| \| \| \| .458 \| \| \| Treatment group main effect, *P* \| \| \| \| \| **.005** \| \| \| T1 vigor/activity \| −14.31 \| 6.53 \| −14.06 \| 6.19 \| NA \| \| \| T2 vigor/activity \| −15.91 \| 6.0 \| −13.57 \| 6.61 \| −2.21 \| −3.67 to −0.75 \| \| T3 vigor/activity \| −16.23 \| 6.63 \| −13.47 \| 6.22 \| −2.63 \| −4.12 to −1.15 \| \| Interaction time × treatment group, *P* \| \| \| \| \| .606 \| \| \| Treatment group main effect, *P* \| \| \| \| \| **< .001** \| \| \| T1 fatigue/inertia \| 11.17 \| 6.64 \| 11.75 \| 7.20 \| NA \| \| \| T2 fatigue/inertia \| 8.71 \| 6.10 \| 11.62 \| 7.16 \| −2.68 \| −4.31 to −1.04 \| \| T3 fatigue/inertia \| 9.27 \| 6.90 \| 11.39 \| 6.73 \| −1.84 \| −3.45 to −0.22 \| \| Interaction time × treatment group, *P* \| \| \| \| \| .324 \| \| \| Treatment group main effect, *P* \| \| \| \| \| **.002** \| \| \| T1 confusion/bewilderment \| 10.11 \| 5.58 \| 10.65 \| 5.57 \| NA \| \| \| T2 confusion/bewilderment \| 8.13 \| 4.71 \| 10.33 \| 5.30 \| −1.91 \| −3.01 to −0.81 \| \| T3 confusion/bewilderment \| 8.24 \| 5.32 \| 9.63 \| 4.31 \| −1.09 \| −2.20 to 0.01 \| \| Interaction time × treatment group, *P* \| \| \| \| \| .141 \| \| \| Treatment group main effect, *P* \| \| \| \| \| **.002** \| \| |
| **Johns 2014** | Global quality of life / Functional impact of fatigue | Functional status: Sheehan Disability Scale (SDS) | Functional disability scores were lower in the MBSR group at T2 (d = −0.45), although not statistically different (p = 0.25); however, at T3 the MBSR group demonstrated significantly lower functional disability scores than controls (p = .0013) with a large effect size (d = −1.22). |
|  | Mood | Patient Health Questionnaire Generalized Anxiety Disorder Scale and Depression severity: PHQ-8. | Depression scores were significantly lower (p < .001) for MBSR than controls with large differences at T2 (d = −1.30) and T3 (d = −1.71). Sleep disturbance was significantly improved for MBSR compared to the control condition at both T2 (d = −0.74) and T3 (d = −1.00). Anxiety scores were lower in the intervention group at T2 than for the control group (d = −0.47), although not statistically different (p = 0.10). By T3, however, the MBSR group demonstrated significantly lower anxiety scores than the control group (p = 0.002) with a large effect size (d = −0.98). |
|  | Insomnia or sleep quality | Sleep disturbance: Insomnia Severity Index | The MBSR group demonstrated significantly greater improvement than the control group in fatigue interference as measured against the Bonferroni-corrected significance level of p < .00278 at T2 and T3. Effect sizes (d) for group differences (adjusted for baseline levels) in fatigue interference were large at both time points, ranging from −1.43 at T2 to −1.34 at T3. |
| **Lengacher 2012** | Global quality of life / Functional impact of fatigue | M.D. Anderson Symptom Inventory (MDASI) | \|  \| Control \| \| \| MBSR(BC) \| \| \| *P* \| \| \| --- \| --- \| --- \| --- \| --- \| --- \| --- \| --- \| --- \| \| Baseline \| 6-Week post-assessment \|  \| Baseline \| 6-Week post-assessment \| *P* \| (between-group post-assessment) \| \| Trouble remembering \| 2.9(2.7) \| 2.0(2.2) \| .03 \| 2.1(2.7) \| 1.3(1.9) \| .05 \| .07 \| \| Drowsy \| 2.6(2.7) \| 1.9(2.0) \| .13 \| 2.2(2.1) \| 1.4(2.2) \| .04 \| .05 \| \| Numbness \| 1.6(2.5) \| 1.4(2.7) \| .34 \| 1.8(2.4) \| 1.1(1.8) \| .07 \| .46 \| \| Dry mouth \| 1.5(2.5) \| 1.1(2.1) \| .08 \| 1.0(1.6) \| .68(1.6) \| .12 \| .60 \| \| Shortness of breath \| 1.1(2.4) \| .83(1.8) \| .57 \| 0.7(1.1) \| .48(1.1) \| .15 \| .21 \| \| Lack of appetite \| 1.0(2.1) \| .73(1.6) \| .11 \| 0.5(1.2) \| .25(.78) \| .15 \| .06 \| \| Nausea \| 0.4(1.7) \| .02(.15) \| .11 \| 0.2(0.6) \| .05(.22) \| .20 \| .53 \| \| Vomiting \| 0.1(0.5) \| 0.0(0.0) \| .32 \| 0.0(0.0) \| .03(.16) \| .32 \| .31 \| \| General activity \| 2.1(3.2) \| 1.6(2.4) \| .41 \| 2.1(2.6) \| .68(1.3) \| .001 \| .12 \| \| Housework \| 2.4(3.2) \| 1.5(2.3) \| .03 \| 2.0(2.7) \| .57(1.3) \| .002 \| .02 \| \| Walking \| 2.2(3.3) \| 1.0(1.8) \| .02 \| 1.5(2.6) \| 1.1(2.2) \| .14 \| .46 \| \| Relationships \| 1.8(3.0) \| .98(1.8) \| .11 \| 1.3(2.1) \| .45(1.4) \| .004 \| .05 \| |
|  | Mood | MDASI mood, enjoyment of life, distress, and sadness | \|  \| Control \| \| \| MBSR(BC) \| \| \| *P* \| \| \| --- \| --- \| --- \| --- \| --- \| --- \| --- \| --- \| --- \| \| Baseline \| 6-Week post-assessment \|  \| Baseline \| 6-Week post-assessment \| *P* \| (between-group post-assessment) \| \| Distress \| 2.2(2.8) \| 1.4(2.2) \| .01 \| 1.7(2.5) \| .82(1.5) \| .02 \| .11 \| \| Sadness \| 2.1(2.8) \| 1.2(2.1) \| .003 \| 2.1(2.6) \| .98(1.8) \| .05 \| .35 \| \| Mood \| 2.4(3.2) \| 1.6(2.4) \| .04 \| 1.8(2.4) \| .70(1.5) \| .005 \| .04 \| \| Enjoyment of life \| 2.3(3.1) \| 1.3(2.1) \| .008 \| 1.6(2.2) \| .63(1.6) \| .003 \| .06 \| |
|  | Insomnia or sleep quality | MDASI sleep disturbance and MDASI drowsiness | the MBSR(BC) group showed greater improvement across symptoms, and especially symptom interference items, compared to the control group. For the MBSR(BC) group, statistically-significant reductions (P < .01) were observed for disturbed sleep.   \|  \| Control \| \| \| MBSR(BC) \| \| \| *P* \| \| --- \| --- \| --- \| --- \| --- \| --- \| --- \| --- \| \| Baseline \| 6-Week post-assessment \|  \| Baseline \| 6-Week post-assessment \| *P* \| (between-group post-assessment) \| \| Disturbed sleep \| 3.1(3.3) \| 2.1(2.9) \| .01 \| 3.2(3.0) \| 1.9(2.5) \| .009 \| .98 \| \| Drowsy \| 2.6(2.7) \| 1.9(2.0) \| .13 \| 2.2(2.1) \| 1.4(2.2) \| .04 \| .05 \| |
|  | Pain | MDASI pain | \|  \| Control \| \| \| MBSR(BC) \| \| \| *P* \| \| \| --- \| --- \| --- \| --- \| --- \| --- \| --- \| --- \| --- \| \| Baseline \| 6-Week post-assessment \|  \| Baseline \| 6-Week post-assessment \| *P* \| (between-group post-assessment) \| \| Pain \| 1.8(2.2) \| 1.9(2.6) \| .73 \| 2.0(2.3) \| 1.4(1.8) \| .04 \| .61 \| |
| **Matthews 2014** | Global quality of life / Functional impact of fatigue | European Organisation for the Research and Treatment of Cancer Quality of Life Questionnaire– Core 30 (EORTC QLQ-C30) | No group differences in improvement were noted relative to QOL |
|  | Mood | Hospital Anxiety and Depression Scale (HADS) | No group differences in improvement were noted relative to mood. |
|  | Insomnia or sleep quality | Sleep parameters extracted from the diary included SL, SE, WASO, TST, and number of nocturnal awakenings.  The Insomnia Severity Index | Sleep efficiency and latency improved more in the CBTI group than the BPT group; this difference was maintained during follow-up. Women in the CBTI group had less subjective insomnia, greater improvements in physical and cognitive functioning, positive sleep attitudes, and increased sleep hygiene knowledge. |
| **Prinsen 2013** | Global quality of life / Functional impact of fatigue | Sickness Impact Profile-8 (SIP-8) | Functional impairment was not significantly different between the intervention and the waiting list group at baseline. The change score in functional impairment (SIP-8) was significantly different between the CBT and the waiting list group (respectively −73.0 ± 28.1 % and −9.5 ± 47.1 %). |
| **Reeves 2017** | Global quality of life / Functional impact of fatigue | SF‐36 | The between‐group intervention effects for other secondary outcomes were not statistically significant. Statistically significant improvements were observed within both arms in physical QoL scores and all body image subscales apart from social barriers. Neither arm changed significantly in mental QoL.   \| Baseline mean (SD) \| \| Mean change (95% CI)[†](https://onlinelibrary.wiley.com/doi/full/10.1111/ajco.12629#ajco12629-tbl3-note-0005_45) \| \| Intervention – usual care[†](https://onlinelibrary.wiley.com/doi/full/10.1111/ajco.12629#ajco12629-tbl3-note-0005_46)^,^[‡](https://onlinelibrary.wiley.com/doi/full/10.1111/ajco.12629#ajco12629-tbl3-note-0006_47) \| \| \| --- \| --- \| --- \| --- \| --- \| --- \| \|  \| Intervention \| Usual care \| Intervention \| Usual care \| Mean difference (95% CI) \| *P* \| \| *Quality of Life (SF‐36)* \|  \|  \|  \|  \|  \|  \| \| Physical component (0–100) \| 46.1 (8.8)[§](https://onlinelibrary.wiley.com/doi/full/10.1111/ajco.12629#ajco12629-tbl3-note-0007_54) \| 45.1 (10.4)[¶](https://onlinelibrary.wiley.com/doi/full/10.1111/ajco.12629#ajco12629-tbl3-note-0008_55) \| 3.4 (1.4, 5.4)[**](https://onlinelibrary.wiley.com/doi/full/10.1111/ajco.12629#ajco12629-tbl3-note-0003_56) \| 4.0 (1.9, 6.1)[***](https://onlinelibrary.wiley.com/doi/full/10.1111/ajco.12629#ajco12629-tbl3-note-0004_57) \| 0.4 (−3.7, 2.9) \| 0.821 \| \| Mental component (0–100) \| 49.4 (8.5)[§](https://onlinelibrary.wiley.com/doi/full/10.1111/ajco.12629#ajco12629-tbl3-note-0007_58) \| 50.5 (10.4)[¶](https://onlinelibrary.wiley.com/doi/full/10.1111/ajco.12629#ajco12629-tbl3-note-0008_59) \| 2.1 (−1.1, 5.3) \| 0.4 (−2.7, 3.5) \| 0.3 (−3.8, 4.5) \| 0.869 \| \| *Treatment‐related side‐effects* \|  \|  \|  \|  \|  \|  \| \| Fatigue (FACIT) (0–52) \| 41 (34, 46)[††](https://onlinelibrary.wiley.com/doi/full/10.1111/ajco.12629#ajco12629-tbl3-note-0009_60) \| 43 (31, 47)[¶](https://onlinelibrary.wiley.com/doi/full/10.1111/ajco.12629#ajco12629-tbl3-note-0008_61) \| 3.0 (0.7, 5.3)[**](https://onlinelibrary.wiley.com/doi/full/10.1111/ajco.12629#ajco12629-tbl3-note-0003_62) \| 1.5 (−1.0, 4.0) \| 1.1 (−2.4, 4.5) \| 0.527 \| \| Body Image (BIRS) \|  \|  \|  \|  \|  \|  \| \| Total (32–160) \| 81.6 (19.6)[¶](https://onlinelibrary.wiley.com/doi/full/10.1111/ajco.12629#ajco12629-tbl3-note-0008_63) \| 82.8 (20.8)[††](https://onlinelibrary.wiley.com/doi/full/10.1111/ajco.12629#ajco12629-tbl3-note-0009_64) \| −8.6 (−13.0, −4.1)[***](https://onlinelibrary.wiley.com/doi/full/10.1111/ajco.12629#ajco12629-tbl3-note-0004_65) \| −10.5 (−15.6, −5.4)[***](https://onlinelibrary.wiley.com/doi/full/10.1111/ajco.12629#ajco12629-tbl3-note-0004_66) \| 1.8 (−6.0, 9.7) \| 0.639 \| \| Strength and health (12–60) \| 32.9 (9.2)[¶](https://onlinelibrary.wiley.com/doi/full/10.1111/ajco.12629#ajco12629-tbl3-note-0008_67) \| 33.7 (8.1)[††](https://onlinelibrary.wiley.com/doi/full/10.1111/ajco.12629#ajco12629-tbl3-note-0009_68) \| −4.4 (−6.6, 2.2)[***](https://onlinelibrary.wiley.com/doi/full/10.1111/ajco.12629#ajco12629-tbl3-note-0004_69) \| −4.5 (−6.8, 2.2)[***](https://onlinelibrary.wiley.com/doi/full/10.1111/ajco.12629#ajco12629-tbl3-note-0004_70) \| −0.9 (−2.8, 4.5) \| 0.627 \| \| Social barriers (9–45) \| 18.5 (6.6)[¶](https://onlinelibrary.wiley.com/doi/full/10.1111/ajco.12629#ajco12629-tbl3-note-0008_71) \| 18.8 (7.9)[††](https://onlinelibrary.wiley.com/doi/full/10.1111/ajco.12629#ajco12629-tbl3-note-0009_72) \| −1.6 (−3.4, 0.2) \| −3.5 (−5.5, −1.6)[***](https://onlinelibrary.wiley.com/doi/full/10.1111/ajco.12629#ajco12629-tbl3-note-0004_73) \| 1.9 (−0.7, 4.6) \| 0.149 \| \| Appearance and sexuality (11–55) \| 30.2 (7.6)[¶](https://onlinelibrary.wiley.com/doi/full/10.1111/ajco.12629#ajco12629-tbl3-note-0008_74) \| 30.3 (8.7)[††](https://onlinelibrary.wiley.com/doi/full/10.1111/ajco.12629#ajco12629-tbl3-note-0009_75) \| −2.6 (−4.6, −0.7)[**](https://onlinelibrary.wiley.com/doi/full/10.1111/ajco.12629#ajco12629-tbl3-note-0003_76) \| −3.1 (−5.6, −0.7)[*](https://onlinelibrary.wiley.com/doi/full/10.1111/ajco.12629#ajco12629-tbl3-note-0002_77) \| −0.3 (−3.8, 3.2) \| 0.866 \| |
| **Reich 2017** | Global quality of life / Functional impact of fatigue | Medical Outcomes Studies Short-Form General Health Survey (SF-36)  M.D. Anderson Symptom Inventory | \| Cluster Psychological \| MBSR(BC) \| \| \| UC \| \| \| \| --- \| --- \| --- \| --- \| --- \| --- \| --- \| \| Mean \| SD \| *n* \| Mean \| SD \| *n* \| \| Emotional well-being (SF-36) \|  \| \| \| \| \| \| \| Baseline \| 63.83 \| 17.42 \| 167 \| 68.57 \| 18.32 \| 155 \| \| Week 6 \| 70.87 \| 18.02 \| 152 \| 69.36 \| 18.78 \| 145 \| \| Week 12 \| 71.33 \| 19.41 \| 153 \| 72.71 \| 19.13 \| 146 \|   From baseline to six weeks, the model demonstrated evidence of MBSR(BC) effectiveness in the psychological (anxiety, depression, perceived stress and QOL, emotional well-being) (P = 0.007) clusters. participants who received the MBSR(BC) training improved more than those who did not at the six-week time point.  standardized regression coefficients (β) for psychological symptoms (.17), effect sizes of 0.35 |
|  | Mood | CES-D  Perceived Stress Scale  State-Trait Anxiety Inventory  Concerns About Recurrence Scale | \| Cluster Psychological \| MBSR(BC) \| \| \| UC \| \| \| \| --- \| --- \| --- \| --- \| --- \| --- \| --- \| \| Mean \| SD \| *n* \| Mean \| SD \| *n* \| \| Depression (CESD) \|  \| \| \| \| \| \| \| Baseline \| 10.87 \| 6.89 \| 167 \| 10.04 \| 6.46 \| 155 \| \| Week 6 \| 8.12 \| 5.45 \| 154 \| 8.82 \| 6.05 \| 146 \| \| Week 12 \| 8.66 \| 6.26 \| 155 \| 8.95 \| 6.80 \| 148 \| \| Anxiety (STAI) \|  \| \| \| \| \| \| \| Baseline \| 38.62 \| 12.30 \| 167 \| 35.86 \| 11.29 \| 155 \| \| Week 6 \| 30.62 \| 12.80 \| 159 \| 31.76 \| 13.20 \| 152 \| \| Week 12 \| 31.82 \| 12.10 \| 155 \| 32.99 \| 13.40 \| 148 \| \| Stress (PSS) \|  \| \| \| \| \| \| \| Baseline \| 17.57 \| 7.71 \| 167 \| 15.39 \| 7.62 \| 155 \| \| Week 6 \| 20.19 \| 5.06 \| 156 \| 20.21 \| 5.33 \| 151 \| \| Week 12 \| 12.90 \| 7.75 \| 158 \| 12.89 \| 8.20 \| 154 \|   From baseline to six weeks, the model demonstrated evidence of MBSR(BC) effectiveness in both the psychological (anxiety, depression, perceived stress and QOL, emotional well-being) (P = 0.007) clusters. participants who received the MBSR(BC) training improved more than those who did not at the six-week time point.  standardized regression coefficients (β) for psychological symptoms (.17), effect sizes of 0.35 |
|  | Insomnia or sleep quality | Pittsburgh Sleep Quality Index | \|  \| MBSR(BC) \| \| \| UC \| \| \| \| --- \| --- \| --- \| --- \| --- \| --- \| --- \| \| Mean \| SD \| *n* \| Mean \| SD \| *n* \| \| Sleep (PSQI) \|  \| \| \| \| \| \| \| Baseline \| 9.12 \| 5.04 \| 165 \| 8.25 \| 4.23 \| 155 \| \| Week 6 \| 7.26 \| 4.47 \| 148 \| 7.52 \| 4.11 \| 145 \| \| Week 12 \| 7.08 \| 4.42 \| 150 \| 7.02 \| 4.12 \| 145 \| \| Drowsiness (MDASI) \|  \| \| \| \| \| \| \| Baseline \| 3.14 \| 3.10 \| 167 \| 2.92 \| 3.14 \| 155 \| \| Week 6 \| 2.32 \| 2.46 \| 152 \| 2.61 \| 2.97 \| 145 \| \| Week 12 \| 2.16 \| 2.85 \| 152 \| 2.33 \| 2.78 \| 147 \| |
|  | Pain | Brief Pain Inventory | Pain (P = 0.97) cluster improvement was not related to assignment.   \| **Cluster Pain** \| **MBSR(BC)** \| \| \| **UC** \| \| \| \| --- \| --- \| --- \| --- \| --- \| --- \| --- \| \| **Mean** \| **SD** \| ***n*** \| **Mean** \| **SD** \| ***n*** \| \| **Quality of life (SF-36)** \|  \| \| \| \| \| \| \| Baseline \| 62.44 \| 27.52 \| 167 \| 62.74 \| 24.68 \| 155 \| \| Week 6 \| 65.76 \| 26.18 \| 152 \| 66.24 \| 24.76 \| 145 \| \| Week 12 \| 68.43 \| 27.76 \| 153 \| 70.36 \| 22.70 \| 146 \| \| **Severity (BPI)** \|  \| \| \| \| \| \| \| Baseline \| 11.30 \| 10.12 \| 167 \| 9.69 \| 8.60 \| 155 \| \| Week 6 \| 9.59 \| 9.44 \| 157 \| 8.28 \| 8.16 \| 151 \| \| Week 12 \| 8.46 \| 9.41 \| 161 \| 8.66 \| 8.40 \| 155 \| |
| **Reif 2012** | Global quality of life / Functional impact of fatigue | EORTC QLQ-C30 | Secondary outcomes also showed significant improvements in all measures, including quality of life (F = 29.607, p < 0.001, η2 = 0.113)   \|  \| **Group** \| **Pre-intervention** \| **Post-intervention** \| **Follow-up at 6 months** \| **Group × time** \| \| **Partial eta-squared** \| \| --- \| --- \| --- \| --- \| --- \| --- \| --- \| --- \| \| **Mean (SD)** \| **Mean (SD)** \| **Mean (SD)** \| ***F*** \| ***p*** \| **Group ×  time** \| \| Global Health Status (range: 0–100) \| IG \| 44.17 (18.32) \| 57.08 (22.93) \| 63.82 (21.67) \| 29.607 \| **<0.001** \| 0.113 \| \| CG \| 43.06 (18.97) \| 40.35 (19.16) \| 39.91 (18.57) \| \| Physical functioning (range: 0–100) \| IG \| 59.28 (20.92) \| 72.33 (19.28) \| 78.55 (20.55) \| 32.432 \| **<0.001** \| 0.123 \| \| CG \| 58.60 (19.92) \| 57.48 (22.74) \| 56.78 (24.15) \| \| Role functioning (range: 0–100) \| IG \| 41.39 (25.20) \| 59.58 (29.36) \| 69.58 (28.96) \| 33.906 \| **<0.001** \| 0.128 \| \| CG \| 39.18 (23.46) \| 37.86 (26.17) \| 38.16 (27.93) \| \| Emotional functioning (range: 0–100) \| IG \| 37.64 (24.89) \| 58.82 (26.42) \| 68.96 (27.14) \| 51.826 \| **<0.001** \| 0.183 \| \| CG \| 37.28 (24.92) \| 36.77 (25.81) \| 33.77 (25.37) \| \| Cognitive functioning (range: 0–100) \| IG \| 41.25 (24.82) \| 60.97 (28.21) \| 68.61 (28.92) \| 48.974 \| **<0.001** \| 0.174 \| \| CG \| 42.25 (26.16) \| 39.77 (27.20) \| 36.70 (27.17) \| \| Social functioning (range: 0–100) \| IG \| 37.08 (28.04) \| 58.33 (31.53) \| 66.11 (32.40) \| 31.282 \| **<0.001** \| 0.119 \| \| CG \| 39.62 (31.39) \| 37.86 (31.14) \| 35.09 (28.60) \| \| CG \| 61.11 (36.28) \| 64.33 (34.84) \| 66.67 (33.77) \| |
|  | Mood | Hospital Anxiety and Depression Scale (HADS-D) | Secondary outcomes also showed significant improvements in all measures, including anxiety (F = 33.194, p < 0.001, η2 = 0.125), and depression (F = 24.604, p < 0.001, η2 = 0.096)   \|  \| Group \| Pre-intervention \| Post-intervention \| Follow-up at 6 months \| Group × time \| \| Partial eta-squared \| \| --- \| --- \| --- \| --- \| --- \| --- \| --- \| --- \| \| Mean (SD) \| Mean (SD) \| Mean (SD) \| *F* \| *p* \| Group × time \| \| Anxiety scale (range: 0–21) \| IG \| 9.16 (3.92) \| 6.73 (4.40) \| 5.32 (4.39) \| 33.194 \| <0.001 \| 0.125 \| \| CG \| 9.51 (3.98) \| 9.47 (3.94) \| 9.81 (4.43) \|  \| \| Depression scale (range: 0–21) \| IG \| 8.32 (3.85) \| 6.09 (4.72) \| 5.04 (4.71) \| 24.604 \| <0.001 \| 0.096 \| \| CG \| 8.71 (3.58) \| 8.77 (3.88) \| 8.86 (4.01) \|  \| |
|  | Insomnia or sleep quality | EORTC QLQ-C30 insomnia subscale | \|  \| **Group** \| **Pre-intervention** \| **Post-intervention** \| **Follow-up at 6 months** \| **Group × time** \| \| **Partial eta-squared** \| \| --- \| --- \| --- \| --- \| --- \| --- \| --- \| --- \| \| **Mean (SD)** \| **Mean (SD)** \| **Mean (SD)** \| ***F*** \| ***p*** \| **Group × time** \| \| Insomnia (range: 0–100) \| IG \| 64.44 (33.12) \| 45.83 (37.44) \| 38.89 (36.24) \| 22.727 \| **<0.001** \| 0.089 \| \| CG \| 61.11 (36.28) \| 64.33 (34.84) \| 66.67 (33.77) \| |
| **Ritterband 2012** | Global quality of life / Functional impact of fatigue | SF-12 | \| Variable \| Internet Participants (n=14) \| \| Control Participants (n=14) \| \| F_1,26_ \| *P* Value \| Overall Adjusted ES (d) \| \| --- \| --- \| --- \| --- \| --- \| --- \| --- \| --- \| \| Mean (SD) \| Pre-Post ES (d)[^*^](https://www.ncbi.nlm.nih.gov/pmc/articles/PMC3424270/table/T3/?report=objectonly#TFN10) \| Mean (SD) \| Pre-Post ES (d)[^*^](https://www.ncbi.nlm.nih.gov/pmc/articles/PMC3424270/table/T3/?report=objectonly#TFN10) \| \| SF-12: Mental \| \| \| \| \| \| \| \| \| Pre \| 43.02 (13.51) \| 0.48 \| 46.86 (7.95) \| 0.00 \| 3.14 \| 0.09 \| 0.48 \| \| Post \| 48.51 (8.73) \| 46.82 (10.06) \| \| SF-12: Physical \| \| \| \| \| \| \| \| \| Pre \| 48.96 (10.36) \| 0.15 \| 45.56 (7.22) \| −0.06 \| 0.44 \| 0.52 \| 0.21 \| \| Post \| 50.36 (9.76) \| 44.96 (10.34) \|   Regarding the SF-12, a measure of quality of life, the group x time interaction for the mental subscale was not significant (p=.09), but the adjusted ES indicated a small-to-medium treatment effect (d=.48). On the physical subscale of the SF-12, the group x time interaction also did not reach significance (p=.52), but the adjusted ES indicated a small treatment effect for SHUTi (d=.21). |
|  | Mood | The Hospital Anxiety and Depression Scale (HADS) | On the total HADS score, a measure of anxiety and depression, the group x time interaction was not significant (p=.09). However, the adjusted effect sizes for the total was d=.52; and the subscales, depression and anxiety, were d=.54 and d=.42, respectively. Regarding the SF-12, a measure of quality of life, the group x time interaction for the mental subscale was not significant (p=.09), but the adjusted ES indicated a small-to-medium treatment effect (d=.48). On the physical subscale of the SF-12, the group x time interaction also did not reach significance (p=.52), but the adjusted ES indicated a small treatment effect for SHUTi (d=.21).   \| Variable \| Internet Participants (n=14) \| \| Control Participants (n=14) \| \| F_1,26_ \| *P* Value \| Overall Adjusted ES (d) \| \| --- \| --- \| --- \| --- \| --- \| --- \| --- \| --- \| \| Mean (SD) \| Pre-Post ES (d)[^*^](https://www.ncbi.nlm.nih.gov/pmc/articles/PMC3424270/table/T3/?report=objectonly#TFN10) \| Mean (SD) \| Pre-Post ES (d)[^*^](https://www.ncbi.nlm.nih.gov/pmc/articles/PMC3424270/table/T3/?report=objectonly#TFN10) \| \| HADS: Total \| \| \| \| \| \| \| \| \| Pre \| 14.64 (7.45) \| 0.73 \| 14.00 (5.19) \| 0.21 \| 3.18 \| 0.09 \| 0.52 \| \| Post \| 9.93 (5.53) \| 12.64 (6.01) \| \| HADS: Depression \| \| \| \| \| \| \| \| \| Pre \| 5.21 (3.58) \| 0.63 \| 5.43 (2.65) \| 0.09 \| 2.08 \| 0.16 \| 0.54 \| \| Post \| 3.21 (2.42) \| 5.14 (4.02) \| \| HADS: Anxiety \| \| \| \| \| \| \| \| \| Pre \| 9.43 (4.29) \| 0.70 \| 8.57 (3.27) \| 0.28 \| 3.15 \| 0.09 \| 0.42 \| \| Post \| 6.71 (3.85) \| 7.50 (2.98) \| |
|  | Insomnia or sleep quality | Pittsburgh Sleep Quality Index | There was a significant group x time interaction effect with the Internet group showing a marked improvement in insomnia severity from pre- to post-assessment, and the control group showing no significant change (F1,26=22.8; p<.01). More specifically, the Internet group dropped from an ISI score of 17.1 at pre-assessment to 8.2 at post-assessment, (t(13)=10.15, p<.01), while the control group showed no significant change: ISI of 15.9 at pre-assessment and 14.4 at post-assessment, (t(13)=1.24, p=0.2;). Per Cohen’s guidelines [54], the adjusted ES indicates a large SHUTi treatment effect for insomnia severity (d=1.85). Gains made by participants who used SHUTi were also clinically significant. At baseline, 9 out of 14 participants (64%) in each group had ISI scores in the “clinically significant” range of insomnia, as defined by an ISI score of greater than 14. The remaining five participants in each group all had ISI scores in the “subthreshold insomnia” range (ISI score in the range of 8 to 14); no participant had an ISI score in the “no insomnia” range (ISI <8). After using SHUTi, only 2 of the 14 (14%) Internet participants still had “clinically significant” levels of insomnia symptoms (ISI >14), compared to 8 of 14 control participants (57%). In addition, 7 of 14 (50%) Internet participants had ISI scores in the “no insomnia” range, compared to just 2 of 14 (14%) control participants.   \| Sleep Variable and Period \| Internet Participants (n=13) \| \| Control Participants (n=13) \| \| F_1,24_ \| *P* Value \| Overall Adjusted ES (d) \| \| --- \| --- \| --- \| --- \| --- \| --- \| --- \| --- \| \| Mean (SD) \| Pre-Post ES (d)[^*^](https://www.ncbi.nlm.nih.gov/pmc/articles/PMC3424270/table/T2/?report=objectonly#TFN4) \| Mean (SD) \| Pre-Post ES (d)[^*^](https://www.ncbi.nlm.nih.gov/pmc/articles/PMC3424270/table/T2/?report=objectonly#TFN4) \| \| Sleep Efficiency, % \| \| \| \| \| \| \| \| \| \| Pre \| 72.16 (9.56) \| 1.05 \| 75.55 (14.13) \| 0.33 \| 11.45 \| < 0.01 \| 0.72 \| \| Post \| 85.67 (6.50)[^a^](https://www.ncbi.nlm.nih.gov/pmc/articles/PMC3424270/table/T2/?report=objectonly#TFN5) \| 79.75 (11.45)[^b^](https://www.ncbi.nlm.nih.gov/pmc/articles/PMC3424270/table/T2/?report=objectonly#TFN6) \| \| Total Sleep Time, min \| \| \| \| \| \| \| \| \| \| Pre \| 361.62 (68.36) \| 0.46 \| 362.46 (73.39) \| 0.14 \| 2.11 \| 0.16 \| 0.32 \| \| Post \| 396.05 (49.64) \| 373.05 (63.60) \| \| SOL, min \| \| \| \| \| \| \| \| \| \| Pre \| 48.42 (32.37) \| 0.83 \| 40.73 (30.57) \| 0.16 \| 5.18 \| 0.03 \| 0.67 \| \| Post \| 19.88 (16.79)[^a^](https://www.ncbi.nlm.nih.gov/pmc/articles/PMC3424270/table/T2/?report=objectonly#TFN5) \| 35.23 (22.31) \| \| WASO, min \| \| \| \| \| \| \| \| \| \| Pre \| 55.88 (30.52) \| 0.72 \| 47.54 (31.25) \| 0.50 \| 1.03 \| 0.32 \| 0.22 \| \| Post \| 31.99 (21.76) \| 30.99 (19.72) \| \| Time In Bed, min \| \| \| \| \| \| \| \| \| \| Pre \| 498.69 (47.45) \| 0.63 \| 481.04 (58.58) \| 0.23 \| 2.56 \| 0.12 \| 0.40 \| \| Post \| 461.42 (39.55) \| 467.31 (42.56) \| \| Awakenings, no. \| \| \| \| \| \| \| \| \| \| Pre \| 2.64 (1.19) \| 0.69 \| 1.98 (.51) \| 0.26 \| 3.05 \| 0.09 \| 0.43 \| \| Post \| 1.87 (.90) \| 1.69 (.59) \| \| Soundness of sleep, scale score[^c^](https://www.ncbi.nlm.nih.gov/pmc/articles/PMC3424270/table/T2/?report=objectonly#TFN7) \| \| \| \| \| \| \| \| \| \| Pre \| 2.55 (.61) \| 1.42 \| 2.85 (.43) \| 0.21 \| 9.34 \| < 0.01 \| 1.21 \| \| Post \| 3.38 (.59)[^a^](https://www.ncbi.nlm.nih.gov/pmc/articles/PMC3424270/table/T2/?report=objectonly#TFN5) \| 2.98 (.69) \| \| Restored, scale score[^d^](https://www.ncbi.nlm.nih.gov/pmc/articles/PMC3424270/table/T2/?report=objectonly#TFN8) \| \| \| \| \| \| \| \| \| \| Pre \| 2.38 (.38) \| 1.51 \| 2.82 (.54) \| 0.16 \| 11.95 \| < 0.01 \| 1.35 \| \| Post \| 3.21 (.60)[^a^](https://www.ncbi.nlm.nih.gov/pmc/articles/PMC3424270/table/T2/?report=objectonly#TFN5) \| 2.91 (.58) \| |
| **Rogers 2017** | Mood | Hospital Anxiety and Depression Scale | Adjusted linear mixed‐model analyses demonstrated significant effects of BEAT Cancer vs usual care on depressive symptomatology (M3 M = −1.3; CI = −2.0 to −0.6; d = −0.38; P < .001), and anxiety (M3 M = −1.3; CI = −2.0 to −0.5; d = −0.33; P < .001). BEAT Cancer effects remained significant at M6 for all outcomes (all P values <.05; d = −0.21 to −.35).   \|  \|  \| Unadjusted Means \|  \| Adjusted[**a**](https://onlinelibrary.wiley.com/doi/full/10.1002/pon.4254#pon4254-note-0002_38) Between‐group Differences Estimated Least Square Mean with (95% CI); *P* Value \| \| \| --- \| --- \| --- \| --- \| --- \| --- \| \| Baseline mean (SD) \| Month 3 mean (SD) \| Month 6 mean (SD) \| BEAT Cancer vs usual care at month 3 (postintervention) \| BEAT Cancer vs usual care at month 6 (3 mo postintervention) \| \| Depression \|  \|  \|  \| −1.3 (−2.0 to −0.6); <.001 \| −0.7 (−1.4 to −0.0); .042 \| \| BEAT Cancer \| 4.8 (3.3) \| 3.0 (2.6) \| 3.5 (3.3) \|  \|  \| \| Usual care \| 4.7 (3.5) \| 4.3 (3.1) \| 4.3 (3.5) \|  \|  \| \| Anxiety \|  \|  \|  \| −1.3 (−2.0 to −0.5); <.001 \| −0.8 (−1.5 to −0.0); .044 \| \| BEAT Cancer \| 7.1 (3.9) \| 5.6 (3.4) \| 5.8 (3.9) \|  \|  \| \| Usual care \| 7.0 (3.9) \| 6.8 (3.5) \| 6.5 (3.7) \|  \|  \| |
| **Sandler 2017** | Mood | Sphere Psychological health subscale | There was no statistically significant change in mood disturbance designated on the PSYCH subscale of the SPHERE over time for the whole sample (F(2,44) = 2.42; P = 0.10) (Fig. 4). In addition, no significant difference was found between groups at end treatment (MEdu = 0.50, SD = 1.62; MInt = 0.65, SD = 2.83; t(44) = −0.23; P = 0.82) or follow-up (MEdu = 0.33, SD = 1.68; MInt = 0.88, SD = 2.41; t(44) = −0.92; P = 0.36). |
|  | Functional status | The 36-item Short Form Health Survey ( SF - 36; RAND ) | A clinically significant improvement in fatigue was observed in 7 of 22 participants in the intervention arm compared with 2 of 24 in the education arm (P < 0.05; χ2) at end treatment. In support of this response designation, these participants had a mean improvement in functional status (role limitation physical—SF-36; M = 34.72 and SD = 35.50) compared with nonresponders (M = 6.89; SD = 17.22; t(43) = 3.4; P < 0.01). By follow-up, 5 of 22 participants in the intervention arm and 6 of 24 in the education arm reported a clinically significant improvement. From the education arm, two participants deteriorated (by 1 SD) at end treatment and four participants at follow-up. No participants from the intervention arm reported deterioration.  Consistent improvements in physical functioning status (SF-36) were observed in all participants between baseline and 12 week (Mdiff = 12.45; 95% CI 3.43–21.48; P < 0.01) and 24 weeks (Mdiff = 14.40; 95% CI 3.86–24.93; P < 0.05). Similarly, improvements in fatigue were mirrored in the interviewer-designated outcomes via the SCIN, with significant decreases in scores from baseline to 12 weeks (Mdiff = −4.05; 95% CI −5.42 to −2.69; P < 0.001) and 24 weeks (Mdiff = −5.23; 95% CI −6.51 to −3.95; P < 0.001). No significant differences in the change scores of physical functioning were observed between the groups at any time point |
|  | Sleep Disturbance | Pittsburgh Sleep Quality Index | An improvement in global sleep scores was observed (F(2,34) = 8.20; P < 0.01) between baseline and end treatment (Mdiff = −2.07; 95% CI −3.43 to −0.72; P < 0.01) and sustained at follow-up 24 weeks (Mdiff = 1.80; 95% CI −3.07 to −0.52; P < 0.01). Insomnia also decreased, evidenced by reductions in the mean time taken to fall asleep (F(2,41) = 4.89; P < 0.05) between baseline and end treatment (Mdiff = −10.62; 95% CI −19.34 to −1.91; P < 0.05), which was maintained at follow-up (Mdiff = 10.49; 95% CI −19.00 to −2.03; P < 0.05). Participants also rated their overall sleep quality as better between baseline and end treatment (Mdiff = −1.73; 95% CI −2.89 to −0.57; P < 0.01) and at follow-up (Mdiff = −1.57; 95% CI −2.66 to −0.49; P < 0.001). No significant difference in global sleep change scores was evident between the education (M = 1.52; SD = 2.96) and intervention groups (M = 2.26; SD = 3.85) at postintervention (t(35) = −0.66, P = 0.51) or follow-up (MEdu = 1.51, SD = 3.37; MInt = 2.18; SD = 2.36; t(37) = −0.69, P = 0.49). |
| **Savard 2005** | Global quality of life / Functional impact of fatigue | European Organization for Research and Treatment of Cancer Quality of Life Questionnaire (QLQ-C30+3) | Significant group-time interactions were obtained on scores of global quality of life (F_1,48_ = 5.69; *P* < .05). A priori contrasts revealed significant time effects in the global quality-of-life scale (F_1,48_ = 16.27; *P* < .001), whereas no significant time effect was found on any variable in the control condition.  Pooled data revealed significant differences between pre- and post-treatment on the global quality-of-life scale (F1,159 = 15.63; P < .0001). No significant difference was detected between post-treatment and the follow-up evaluations.   \| **Variable** \|  \| **Cognitive-Behavioral Therapy (n = 27)** \|  \| **Waiting-List Control (n = 30)** \| \|  \| \| --- \| --- \| --- \| --- \| --- \| --- \| --- \| \| **Mean** \| **95% CI** \| **Mean** \| **95% CI** \| **Mean** \| **95% CI** \| \| QLQ-C33 (global) \|  \|  \|  \|  \|  \|  \| \| Prewaiting \| — \| — \| 67.08 \| 60.10 to 74.06 \| — \| — \| \| Pretreatment[*](javascript:popRef('T4FN1')) \| 52.88 \| 45.80 to 59.96 \| 70.10 \| 63.18 to 77.02 \| 61.49 \| 56.55 to 66.43 \| \| Post-treatment \| 67.56 \| 60.07 to 75.05 \| 74.93 \| 68.01 to 81.85 \| 71.24 \| 66.14 to 76.34 \| \| 3-month follow-up \| 70.79 \| 62.81 to 78.77 \| 75.68 \| 68.39 to 82.97 \| 73.23 \| 67.82 to 78.64 \| \| 6-month follow-up \| 69.83 \| 61.85 to 77.81 \| 73.77 \| 66.26 to 81.28 \| 71.80 \| 66.33 to 77.27 \| \| 12-month follow-up \| 75.51 \| 66.67 to 84.35 \| 73.47 \| 65.98 to 80.96 \| 74.49 \| 68.71 to 80.27 \| |
|  | Mood | Hospital Anxiety and Depression Scale. | Significant group-time interactions were obtained on scores of anxiety (F_1,45_ = 5.19; *P* < .05) and depression (F_1,48_ = 4.14; *P* < .05). A priori contrasts revealed significant time effects in the treatment condition on anxiety (F_1,46_ = 4.77; *P* < .05), and the depression (F_1,49_ = 9.03; *P* < .01) scale, whereas no significant time effect was found on any variable in the control condition.  Pooled data revealed significant differences between pre- and post-treatment on anxiety (F1,150 = 11.10; P < .001), depression (F1,146 = 11.87; P < .001). No significant difference was detected between post-treatment and the follow-up evaluations on any of these variables.   \|  \|  \| **Cognitive-Behavioral Therapy (n = 27)** \|  \| **Waiting-List Control (n = 30)** \| \|  \| \| --- \| --- \| --- \| --- \| --- \| --- \| --- \| \| **Mean** \| **95% CI** \| **Mean** \| **95% CI** \| **Mean** \| **95% CI** \| \| HADS-A \|  \|  \|  \|  \|  \|  \| \| Prewaiting \| — \| — \| 6.57 \| 5.14 to 8.00 \| — \| — \| \| Pretreatment[*](javascript:popRef('T4FN1')) \| 8.61 \| 7.14 to 10.08 \| 7.21 \| 5.90 to 8.52 \| 7.91 \| 6.93 to 8.89 \| \| Post-treatment \| 7.23 \| 5.74 to 8.72 \| 5.99 \| 4.68 to 7.30 \| 6.61 \| 5.61 to 7.61 \| \| 3-month follow-up \| 5.86 \| 4.37 to 7.35 \| 5.66 \| 4.29 to 7.03 \| 5.76 \| 4.74 to 6.78 \| \| 6-month follow-up \| 5.34 \| 3.83 to 6.85 \| 5.71 \| 4.30 to 7.12 \| 5.52 \| 4.48 to 6.56 \| \| 12-month follow-up \| 6.19 \| 4.52 to 7.86 \| 4.78 \| 3.37 to 6.19 \| 5.48 \| 4.38 to 6.58 \| \| HADS-D \|  \|  \|  \|  \|  \|  \| \| Prewaiting \| — \| — \| 2.83 \| 1.93 to 3.73 \| — \| — \| \| Pretreatment[*](javascript:popRef('T4FN1')) \| 4.64 \| 3.74 to 5.54 \| 2.62 \| 1.82 to 3.42 \| 3.63 \| 3.02 to 4.24 \| \| Post-treatment \| 2.90 \| 1.96 to 3.84 \| 2.29 \| 1.49 to 3.09 \| 2.60 \| 1.97 to 3.23 \| \| 3-month follow-up \| 2.66 \| 1.72 to 3.60 \| 1.99 \| 1.15 to 2.83 \| 2.33 \| 1.70 to 2.96 \| \| 6-month follow-up \| 2.37 \| 1.45 to 3.29 \| 1.83 \| 0.95 to 2.71 \| 2.10 \| 1.45 to 2.75 \| \| 12-month follow-up \| 2.41 \| 1.35 to 3.47 \| 1.68 \| 0.82 to 2.54 \| 2.04 \| 1.35 to 2.73 \| |
|  | Insomnia or sleep quality | The Insomnia Interview Schedule Insomnia Severity Index And sleep diary  sleep onset latency  sleep onset  total wake time  total sleep time  sleep efficiency  use of sleep-promoting medications | Significant group-time interactions were obtained on all sleep variables, with the exception of total sleep time: sleep efficiency (F_1,52_ = 22.59; *P* < .0001), total wake time (F_1,52_ = 22.77; *P* < .001), sleep onset latency F_1,53_ = 4.16; *P* < .05), wake after sleep onset (F_1,52_ = 16.70; *P* < .001), ISI-P (F_1,52_ = 25.31; *P* < .0001), ISI-C (F_1,52_ = 79.37; *P* < .0001), and ISI-SO (F_1,48_ = 4.54; *P* < .05). A priori contrasts revealed significant time effects on all variables in the treatment condition and all variables with the exception of two in the control condition (sleep onset latency and wake after sleep onset). Significant time effects found in the control condition were always of a lower magnitude compared with those of the treatment condition. For instance, sleep efficiency increased from 69.5% to 84.4% at post-treatment in the experimental condition, whereas it increased only from 71.1% to 74.5% in the control condition during the waiting period. An analysis was conducted to investigate whether hypnotic use at pretreatment had a moderating role in the effect of CBT on subjective sleep measures at post-treatment. No significant hypnotic use-group-time interaction was found on any of these sleep variables (*P* from .28 to .93). |
| **Van Der Lee 2012** | Global quality of life / Functional impact of fatigue | Sickness Impact Profile. Dutch Health and Disease Inventory questionnaire | \| Functional impairment \| Follow up \| \| Difference with baseline \| \| Difference with post-measurement \| \| \| --- \| --- \| --- \| --- \| --- \| --- \| --- \| \|  \| Mean \| SD \| 95% CI \| p \| 95% CI \| p \| \| MBCT (N= 56) \| 11.9 \| 12.9 \| 1.4 to 8.4 \| 0.01 \| 1.5 to 4.8 \| 0.30 \| \| Well-being MBCT (N = 56) \| 54.2 \| 9.2 \| 9.8 to 5.4 \| 0.00 \| 4.2 to 0.4 \| 0.02 \|   Six months after the intervention, participants reported significantly functional impairment than at baseline. Treatment effects at postmeasurement were maintained for functional impairment. |
|  | Mood | Hospital Anxiety Depression Scale  **Control variable | About a quarter of all participants (25.8%) scored above the cut-of score of the HADS at baseline. A Chi-square test revealed no differences in percentage of depressive cases between the intervention and the waiting-list control group: (p 5 0.371). |
|  | Insomnia or sleep quality | Sleep Quality Scale—SQS  **Control variable | One-third of all participants (30.6%) suffered from sleep disturbances (25% in the waiting-list control group; 32% in the intervention group). A Chi-square test revealed no differences in percentage of cases of sleep disturbance between the intervention and the waiting-list control group (p 5 0.718). |
| **Van Weert 2010** |  | | |
| **Willems 2016** | Global quality of life / Functional impact of fatigue | EORTC QLQ-C30 | \|  \| \| Mixed models (*n* = 414) \| \| \| \| Imputed data (*n* = 462) \| \| \| --- \| --- \| --- \| --- \| --- \| --- \| --- \| --- \| \| 95% CI \| *p* \| *p_fdr_* \| *d* [95% CI] \| 95% CI \| *p* \| \| Emotional functioning \|  \|  \|  \|  \|  \|  \|  \| \| 6 months \| Crude \| 0.18–6.25 \| .038 \| .038 \| −0.15 [−0.34–0.05] \| −0.77–5.48 \| .139 \| \| Adjusted \| 0.02–6.07 \| .049 \| .049 \|  \| −1.15–5.00 \| .221 \| \| 12 months \| Crude \| −0.35–5.93 \| .081 \| .661 \| −0.08 [−0.28–0.12] \| −3.01–3.56 \| .871 \| \| Adjusted \| −0.47–5.78 \| .096 \| .384 \|  \| −3.43–3.11 \| .923 \| \| Social functioning \|  \|  \|  \|  \|  \|  \|  \| \| 6 months \| Crude \| 0.41–6.87 \| .027 \| .037 \| −0.15 [−0.35–0.04] \| −2.22–4.96 \| .453 \| \| Adjusted \| 0.35–6.66 \| .030 \| .048 \|  \| −2.45–4.53 \| .562 \| \| 12 months \| Crude \| −1.97–4.73 \| .421 \| .661 \| −0.02 [−0.22–0.18] \| −6.57–0.54 \| .096 \| \| Adjusted \| −1.97 – 4.59 \| .435 \| .580 \|  \| −6.80–0.10 \| .057 \| |
|  | Mood | Hospital Anxiety and Depression Scale (HADS) | \|  \| \| Mixed models (*n* = 414) \| \| \| \| Imputed data (*n* = 462) \| \| \| --- \| --- \| --- \| --- \| --- \| --- \| --- \| --- \| \| 95% CI \| *p* \| *p_fdr_* \| *d* [95% CI] \| 95% CI \| *p* \| \|  \| Adjusted \| −1.97 – 4.59 \| .435 \| .580 \|  \| −6.80–0.10 \| .057 \| \| Depression \|  \|  \|  \|  \|  \|  \|  \| \| 6 months \| Crude \| −0.90–−0.11 \| .011 \| .037 \| 0.21 [0.01–0.40] \| −0.93–−0.10 \| .014 \| \| Adjusted \| −0.86–−0.07 \| .021 \| .048 \|  \| −0.82–−0.00 \| .049 \| \| 12 months \| Crude \| −0.70–0.10 \| .145 \| .661 \| 0.10 [−0.11–0.30] \| −0.60–0.23 \| .375 \| \| Adjusted \| −0.66 – 0.16 \| .227 \| .454 \|  \| −0.50–0.33 \| .684 \| \| Adjusted \| −4.90–2.88 \| .611 \| .661 \|  \|  \|  \| |
| **Yun 2017** | Mood | PTGI/ Hospital Anxiety and Depression Scale (HADS) | The LP group showed a significantly greater decrease in the HADS anxiety score (p = 0.025).   \|  \|  \| **Unadjusted estimates, mean (SD)** \| \| **Adjusted analysis for intervention vs usual care^a^** \| \| \| \| --- \| --- \| --- \| --- \| --- \| --- \| --- \| \| **Intervention group** \| **Control group** \| **Intervention group** \| **Control group** \| ***P*value^1)^** \| \| HADS \| \| \| \| \| \| \| \| Anxiety \| Baseline \| 5.7 (3.4) \| 5.9 (3.1) \|  \|  \|  \| \| 3 months \| 5.0 (3.0) \| 6.1 (3.1) \| 5.2 (0.2) \| 6.0 (0.3) \| **0.025**** \| \| 12 months \| 5.1 (3.0) \| 5.8 (2.9) \| 5.2 (0.3) \| 5.7 (0.4) \| 0.228 \| \| Depression \| Baseline \| 6.4 (3.5) \| 6.1 (3.1) \|  \|  \|  \| \| 3 months \| 5.5 (3.3) \| 5.4 (2.8) \| 5.6 (0.2) \| 5.6 (0.3) \| 0.986 \| \| 12 months \| 5.4 (3.4) \| 5.6 (3.1) \| 5.3 (0.3) \| 5.7 (0.4) \| 0.428 \| |
|  | Global quality of life / Functional impact of fatigue | EORTC Quality of Life Questionnaire (EORTC QLQ-C30) | A significantly greater increase in the social functioning score of the EORTC QLQ-C30 (p = 0.018), and a significantly greater decrease in the appetite loss (p = 0.048) and financial difficulties scores (p = 0.036) of the EORTC QLQ-C30 from baseline to 3 months. From baseline to 12 months, the LP group, relative to the UC group, showed a significantly greater decrease in the EORTC QLQ-C30 fatigue score (p = 0.065)   \|  \|  \| **Unadjusted estimates, mean (SD)** \| \| **Adjusted analysis for intervention vs usual care^a^** \| \| \| \| --- \| --- \| --- \| --- \| --- \| --- \| --- \| \| **Intervention group** \| **Control group** \| **Intervention group** \| **Control group** \| ***P*value^1)^** \| \| EORTC QLQ-C30 \| \| \| \| \| \| \| \| Functional scales \| \| \| \| \| \| \| \| Global health status \| Baseline \| 64.5 (19.9) \| 63.4 (18.7) \|  \|  \|  \| \| 3 months \| 67.7 (18.7) \| 65.7 (17.5) \| 67.0 (1.6) \| 66.0 (2.3) \| 0.705 \| \| 12 months \| 70.1 (17.1) \| 65.3 (17.9) \| 69.0 (1.6) \| 66.0 (2.2) \| 0.269 \| \| Physical functioning \| Baseline \| 78.6 (13.5) \| 77.9 (11.1) \|  \|  \|  \| \| 3 months \| 80.0 (12.1) \| 78.4 (12.0) \| 79.4 (0.9) \| 79.3 (1.3) \| 0.942 \| \| 12 months \| 82.9 (13.1) \| 78.2 (12.4) \| 81.9 (1.2) \| 78.7 (1.6) \| 0.123 \| \| Role functioning \| Baseline \| 79.4 (21.4) \| 77.9 (19.8) \|  \|  \|  \| \| 3 months \| 80.9 (18.1) \| 77.3 (18.4) \| 80.3 (1.5) \| 78.5 (2.2) \| 0.497 \| \| 12 months \| 82.7 (19.8) \| 79.9 (18.9) \| 80.9 (1.8) \| 81.1 (2.4) \| 0.958 \| \| Emotional functioning \| Baseline \| 76.8 (19.4) \| 73.0 (23.0) \|  \|  \|  \| \| 3 months \| 78.0 (19.1) \| 74.5 (16.5) \| 76.7 (1.5) \| 75.3 (2.2) \| 0.602 \| \| 12 months \| 78.0 (19.9) \| 75.9 (18.3) \| 76.2 (1.9) \| 77.7 (2.4) \| 0.625 \| \| Cognitive functioning \| Baseline \| 76.7 (19.9) \| 72.6 (20.9) \|  \|  \|  \| \| 3 months \| 80.1 (17.2) \| 72.5 (20.2) \| 77.6 (1.4) \| 75.1 (2.4) \| 0.322 \| \| 12 months \| 78.1 (14.9) \| 76.5 (19.2) \| 76.8 (1.6) \| 78.4 (2.1) \| 0.552 \| \| Social functioning \| Baseline \| 75.8 (26.8) \| 73.1 (23.4) \|  \|  \|  \| \| 3 months \| 85.4 (19.3) \| 76.3 (20.2) \| 84.8 (1.8) \| 77.4 (2.5) \| 0.018 \| \| 12 months \| 85.3 (19.5) \| 78.2 (22.4) \| 84.8 (2.2) \| 79.0 (2.9) \| 0.123 \| |
|  | Insomnia or sleep quality | EORTC Quality of Life Questionnaire (EORTC QLQ-C30)  Medical Outcome Study–Sleep Scale (MOS-SS) Sleep Quality Index I and II | \|  \|  \| **Unadjusted estimates, mean (SD)** \| \| **Adjusted analysis for intervention vs usual care^a^** \| \| \| \| --- \| --- \| --- \| --- \| --- \| --- \| --- \| \| **Intervention group** \| **Control group** \| **Intervention group** \| **Control group** \| ***P*value^1)^** \| \| EORTC QLQ-C30 \| \| \| \| \| \| \| \| Symptom scales \| \| \| \| \| \| \| \| Insomnia \| Baseline \| 28.8 (30.0) \| 30.3 (28.9) \|  \|  \|  \| \| 3 months \| 24.1 (24.50 \| 26.7 (26.9) \| 25.0 (2.1) \| 25.7 (3.1) \| 0.850 \| \| 12 months \| 26.2 (27.9) \| 32.0 (27.2) \| 27.6 (2.5) \| 29.1 (3.4) \| 0.732 \| \| The MOS-SSS \| Baseline \| 65.6 (20.9) \| 65.6 (20.6) \|  \|  \|  \| \| 3 months \| 66.3 (21.1) \| 67.9 (19.3) \| 66.9 (1.4) \| 65.7 (2.0) \| 0.621 \| \| 12 months \| 66.6 (21.1) \| 68.0 (19.7) \| 67.1 (1.7) \| 65.3 (2.4) \| 0.535 \| |
|  | Pain | EORTC Quality of Life Questionnaire (EORTC QLQ-C30) | \|  \|  \| **Unadjusted estimates, mean (SD)** \| \| **Adjusted analysis for intervention vs usual care^a^** \| \| \| \| --- \| --- \| --- \| --- \| --- \| --- \| --- \| \| **Intervention group** \| **Control group** \| **Intervention group** \| **Control group** \| ***P*value^1)^** \| \| EORTC QLQ-C30 \| \| \| \| \| \| \| \| Symptom scales \| \| \| \| \| \| \| \| Pain \| Baseline \| 15.4 (19.2) \| 21.4 (19.0) \|  \|  \|  \| \| 3 months \| 11.9 (16.0) \| 19.6 (19.6) \| 13.6 (1.5) \| 17.4 (2.1) \| 0.146 \| \| 12 months \| 13.1 (17.6) \| 19.7 (21.4) \| 15.5 (1.8) \| 16.2 (2.3) \| 0.810 \| |
| **Yun 2012** | Global quality of life / Functional impact of fatigue | EORTCQLQ-C30 | the intervention group experienced a significantly greater improvement in global quality of life (5.22; 95% CI, 0.93 to 9.50)   \| Outcome \| Intervention Group (n = 136) \| \| \| \| Control Group (n = 137) \| \| \| \| Group Difference[*](javascript:popRef('TF2-1')) \| \| Adj *P* [*](javascript:popRef('TF2-1')) \| Effect Size[†](javascript:popRef('TF2-2')) \| \| --- \| --- \| --- \| --- \| --- \| --- \| --- \| --- \| --- \| --- \| --- \| --- \| --- \| \| Baseline \| \| Change at3 Months \| \| Baseline \| \| Change at3 Months \| \| \| Mean \| SD \| Mean \| SD \| Mean \| SD \| Mean \| SD \| Mean \| 95% CI \| \| EORTC-C30 \|  \|  \|  \|  \|  \|  \|  \|  \|  \|  \|  \|  \| \| Global health status/QOL \| 61.15 \| 19.41 \| 7.60 \| 19.42 \| 59.79 \| 19.17 \| 2.62 \| 19.59 \| 5.22 \| 0.93 to 9.50 \| .**017** \| 0.26 \| \| Functional scales \|  \|  \|  \|  \|  \|  \|  \|  \|  \|  \|  \|  \| \| Physical \| 72.01 \| 15.11 \| 6.86 \| 11.92 \| 72.80 \| 16.05 \| 4.57 \| 13.38 \| 2.13 \| −0.45 to 4.72 \| .106 \| 0.18 \| \| Role \| 71.45 \| 24.76 \| 6.50 \| 19.37 \| 72.51 \| 23.45 \| 4.01 \| 20.16 \| 1.90 \| −2.02 to 5.83 \| .340 \| 0.13 \| \| Emotional \| 70.16 \| 21.31 \| 5.02 \| 17.98 \| 67.21 \| 21.77 \| 1.64 \| 18.58 \| 4.69 \| 0.69 to 8.69 \| **.022** \| 0.19 \| \| Cognitive \| 73.41 \| 19.18 \| 5.15 \| 16.29 \| 69.59 \| 23.04 \| 0.73 \| 18.62 \| 6.09 \| 2.23 to 9.94 \| **.002‖** \| 0.25 \| \| Social \| 76.84 \| 23.50 \| 7.97 \| 21.75 \| 76.28 \| 22.75 \| 3.04 \| 19.62 \| 4.73 \| 0.53 to 8.93 \| **.027** \| 0.24 \| |
|  | Mood | HADS | the intervention group experienced a significantly greater decrease in HADS anxiety score (-0.90; 95% CI,-1.51 to --0.29)   \| Outcome \| Intervention Group (n = 136) \| \| \| \| Control Group (n = 137) \| \| \| \| Group Difference[*](javascript:popRef('TF2-1')) \| \| Adj *P* [*](javascript:popRef('TF2-1')) \| Effect Size[†](javascript:popRef('TF2-2')) \| \| --- \| --- \| --- \| --- \| --- \| --- \| --- \| --- \| --- \| --- \| --- \| --- \| --- \| \| Baseline \| \| Change at3 Months \| \| Baseline \| \| Change at3 Months \| \| \| Mean \| SD \| Mean \| SD \| Mean \| SD \| Mean \| SD \| Mean \| 95% CI \| \| HADS score \|  \|  \|  \|  \|  \|  \|  \|  \|  \|  \|  \|  \| \| Anxiety \| 6.42 \| 3.83 \| −0.79 \| 2.79 \| 6.52 \| 3.86 \| 0.11 \| 2.59 \| −0.90 \| −1.51 to −0.29 \| **.004** \| 0.33 \| \| Depression \| 5.65 \| 3.09 \| −0.39 \| 3.08 \| 5.73 \| 3.30 \| −0.12 \| 2.81 \| −0.28 \| −0.93 to 0.36 \| .387 \| 0.09 \| |
|  | Insomnia or sleep quality | Medical Outcome Study–Sleep Scale (MOS-SS) Sleep Quality Index I and II | \| Outcome \| Intervention Group (n = 136) \| \| \| \| Control Group (n = 137) \| \| \| \| Group Difference[*](javascript:popRef('TF2-1')) \| \| Adj *P* [*](javascript:popRef('TF2-1')) \| Effect Size[†](javascript:popRef('TF2-2')) \| \| --- \| --- \| --- \| --- \| --- \| --- \| --- \| --- \| --- \| --- \| --- \| --- \| --- \| \| Baseline \| \| Change at3 Months \| \| Baseline \| \| Change at3 Months \| \| \| Mean \| SD \| Mean \| SD \| Mean \| SD \| Mean \| SD \| Mean \| 95% CI \| \| MOS-SS \|  \|  \|  \|  \|  \|  \|  \|  \|  \|  \|  \|  \| \| Sleep Quality Index I \| 31.52 \| 15.70 \| −3.11 \| 12.58 \| 33.33 \| 17.82 \| −1.58 \| 10.67 \| −2.04 \| −4.53 to 0.44 \| .106 \| 0.13 \| \| Sleep Quality Index II \| 32.16 \| 16.01 \| −3.08 \| 12.11 \| 33.76 \| 18.02 \| −1.40 \| 11.37 \| −2.04 \| −4.57 to 0.49 \| .114 \| 0.14 \| |
|  | Pain | Brief Pain Inventory | \| Outcome \| Intervention Group (n = 136) \| \| \| \| Control Group (n = 137) \| \| \| \| Group Difference[*](javascript:popRef('TF2-1')) \| Adj *P* [*](javascript:popRef('TF2-1')) \| Effect Size[†](javascript:popRef('TF2-2')) \| \| \| --- \| --- \| --- \| --- \| --- \| --- \| --- \| --- \| --- \| --- \| --- \| --- \| --- \| \| Baseline \| \| Change at3 Months \| \| Baseline \| \| Change at3 Months \| \| \| Mean \| SD \| Mean \| SD \| Mean \| SD \| Mean \| SD \| Mean \| 95% CI \|  \| \| BPI score \|  \|  \|  \|  \|  \|  \|  \|  \|  \|  \|  \|  \| \| Severity \| 2.12 \| 1.76 \| −0.45 \| 1.46 \| 2.35 \| 2.00 \| −0.42 \| 2.00 \| −0.13 \| −0.49 to 0.22 \| .458 \| 0.01 \| \| Interference \| 1.86 \| 1.96 \| −0.49 \| 1.76 \| 2.02 \| 2.16 \| −0.27 \| 1.75 \| −0.28 \| −0.63 to 0.06 \| .110 \| 0.13 \| |
