# Supplementary material for: The effectiveness of psychological interventions for fatigue in cancer survivors: systematic review of randomised controlled trials
Source: Syst Rev. 2019 Dec 13;8:324. doi: 10.1186/s13643-019-1230-2 (PMC6911282; doi:10.1186/s13643-019-1230-2)
Supplement: Supplementary file 2 — Additional file 2. Risk of Bias Assessment. [file 13643_2019_1230_MOESM2_ESM.docx]

Additional file 2. Risk of Bias Assessment

| **Bantum 2014** | | |
| --- | --- | --- |
| **Bias** | **Authors' judgement** | **Support for judgement** |
| Random sequence generation (selection bias) | Low risk | Randomized using a random number table |
| Allocation concealment (selection bias) | Unclear risk | Not specified |
| Blinding of participants and personnel (performance bias) | High risk | Not possible |
| Blinding of outcome assessment (detection bias) | Unclear risk | Not specified |
| Incomplete outcome data (attrition bias) | Low risk | (<20%) Roughly 14% (13.9%, 49/352) of participants who were randomized did not provide any data at 6 months, which did not differ by condition (11.4%, 20/176 and 16.5%, 29/176) for control and intervention, respectively). |
| Selective reporting (reporting bias) | Low risk | All outcomes pre-specified by authors reported.  Trial registered: Clinicaltrials.gov NCT00962494 |
| Other bias | Low risk | The trial appears to be free of other problems that could put it at a high risk of bias |
| **Bennett 2007** | | |
| **Bias** | **Authors' judgement** | **Support for judgement** |
| Random sequence generation (selection bias) | Low risk | “a physical activity counselor assigned each participant to either the intervention or the control group according to a computer-generated randomization scheme.” |
| Allocation concealment (selection bias) | Unclear risk | “assignments were placed in sealed envelopes prior to study.” |
| Blinding of participants and personnel (performance bias) | High risk | “The physical activity counselor who conducted the MI intervention was not blinded to group assignment.” |
| Blinding of outcome assessment (detection bias) | High risk | “The physical activity counselor who conducted the outcome measurements was not blinded to group assignment.” |
| Incomplete outcome data (attrition bias) | Low risk | **<**20% attrition from both arms at follow-up |
| Selective reporting (reporting bias) | Low risk | All outcomes pre-specified by authors reported |
| Other bias | Unclear risk | The trial appears to be free of other problems that could put it at a high risk of bias |
| **Blaes 2016** |  |  |
| **Bias** | **Authors' judgement** | **Support for judgement** |
| Random sequence generation (selection bias) | Low risk | Randomization was done by a SAS random number generator. |
| Allocation concealment (selection bias) | Unclear risk | Not specified |
| Blinding of participants and personnel (performance bias) | Unclear risk | Not specified. (Faculty delivered the intervention ) |
| Blinding of outcome assessment (detection bias) | Unclear risk | Blinding of outcome assessors not specified |
| Incomplete outcome data (attrition bias) | Low risk | 3/27 MBCR; 2/13 CONTROL  One participant withdrew from the study because of progressive disease. |
| Selective reporting (reporting bias) | Low risk | All outcomes pre-specified by authors reported  Trial registered: Clinicaltrials.gov NCT01601548 |
| Other bias | Unclear risk | The trial appears to be free of other problems that could put it at a high risk of bias |
| **Bower 2015** |  |  |
| **Bias** | **Authors' judgement** | **Support for judgement** |
| Random sequence generation (selection bias) | Unclear risk | Not specified |
| Allocation concealment (selection bias) | Unclear risk | condition assignments were kept in sealed envelopes in the research office, |
| Blinding of participants and personnel (performance bias) | High risk | Not possible |
| Blinding of outcome assessment (detection bias) | Unclear risk | Not specified |
| Incomplete outcome data (attrition bias) | Low risk | Follow up of 92% at the primary endpoint. 83% completed the 3-month follow-up questionnaire |
| Selective reporting (reporting bias) | Low risk | All outcomes pre-specified by authors reported.  Trial registered Clinicaltrials.gov NCT01558258. |
| Other bias | Unclear risk | Participants were recruited through invitations to women who had enrolled in an earlier study |
| **Bruggeman-Everts 2017** |  |  |
| **Bias** | **Authors' judgement** | **Support for judgement** |
| Random sequence generation (selection bias) | Low risk | Randomized via a computerized tool: an embedded automated randomization function |
| Allocation concealment (selection bias) | Unclear risk | Researchers could neither influence nor predict the outcome of the randomization process. |
| Blinding of participants and personnel (performance bias) | High risk | Neither researchers, participants, nor therapists were blind to treatment, as the medical ethical committee insisted that we announced the minimal intervention as our control group. |
| Blinding of outcome assessment (detection bias) | Low risk | independent statistician (RvdS) was blind to allocation while checking all analyses. |
| Incomplete outcome data (attrition bias) | High risk | Proportion of participants who dropped out the intervention before completing 6 weeks of the protocol, was 18% (11/62) in the AAF condition, 38% (21/55) in the eMBCT condition, and 6% (3/50) in the psycho-education condition. |
| Selective reporting (reporting bias) | Low risk | All outcomes pre-specified by authors reported.  Trial registered: Trialregister.nl NTR3483 |
| Other bias | Unclear risk | The trial appears to be free of other problems that could put it at a high risk of bias |
| **Carlson 2016** |  |  |
| **Bias** | **Authors' judgement** | **Support for judgement** |
| Random sequence generation (selection bias) | Low risk | Women were assigned randomly using the Research Randomiizer website( <http://www.randomizer.org/>)  2:2:1 ( 2 conditions and a control group ) by the Statistician |
| Allocation concealment (selection bias) | Low risk | Central allocation by random generator used by Statistician |
| Blinding of participants and personnel (performance bias) | Unclear risk | At the time of initial assessment, participants as well as RAs were blind to condition. |
| Blinding of outcome assessment (detection bias) | Unclear risk | Not specified |
| Incomplete outcome data (attrition bias) | High risk | 165 women completed their pre‐intervention and post‐intervention assessment (MBCR: 74 + 13 = 87; SET: 73 + 5 = 78), 65% of the original sample. At 6 months, 130 women completed the follow‐up assessment (51.5%), and 128 women completed the 12‐month follow‐up assessment (50.8%). |
| Selective reporting (reporting bias) | Unclear risk | All outcomes pre-specified by authors reported.  Trial registered Clinicaltrials.gov NCT00390169  The follow up study assessed a broader range of outcome measures than the primary study |
| Other bias | Unclear risk | The trial appears to be free of other problems that could put it at a high risk of bias |
| **Dirksen 2008** |  |  |
| **Bias** | **Authors' judgement** | **Support for judgement** |
| Random sequence generation (selection bias) | Low risk | A random numbers table. |
| Allocation concealment (selection bias) | Unclear risk | Not specified- assigned to treatment groups by the research assistant |
| Blinding of participants and personnel (performance bias) | High risk | “The research assistant was not blinded to the group assignment”  Participants: due to the nature of the intervention content, participants could not have been blinded |
| Blinding of outcome assessment (detection bias) | Unclear risk | Not specified |
| Incomplete outcome data (attrition bias) | Low risk | <20% attrition |
| Selective reporting (reporting bias) | Low risk | All outcomes specified in methods reported in results |
| Other bias | Low risk | The trial appears to be free of other problems that could put it at a high risk of bias |
| **Dodds 2015** |  |  |
| **Bias** | **Authors' judgement** | **Support for judgement** |
| Random sequence generation (selection bias) | Low risk | Randomization was performed by the study biostatistician using stratified block randomization using random block size, as implemented in the ralloc module of the Stata statistical software package |
| Allocation concealment (selection bias) | Unclear risk | Not specified |
| Blinding of participants and personnel (performance bias) | Unclear risk | Study participants were blinded to group assignment until completion of all baseline assessments. The interventionist delivering CBCT could not be blinded. |
| Blinding of outcome assessment (detection bias) | Unclear risk | Not specified |
| Incomplete outcome data (attrition bias) | High risk | Of the 33 randomized participants, 22 had follow-up data (67 %, 95 % CI 48, 82 %), slightly less than the targeted proportion of 70 %. |
| Selective reporting (reporting bias) | Unclear risk | No published protocol but reported everything they said they would in the paper |
| Other bias | Unclear risk | No correction was made for multiple comparisons |
| **Dolbeault 2009** |  |  |
| **Bias** | **Authors' judgement** | **Support for judgement** |
| Random sequence generation (selection bias) | Low risk | Randomization by sealed letter was performed at each site, with a readjustment of the number of subjects in each group after every eighth subject. |
| Allocation concealment (selection bias) | Unclear risk | a readjustment of the number of subjects in each group after every eighth subject |
| Blinding of participants and personnel (performance bias) | Unclear risk | Not possible |
| Blinding of outcome assessment (detection bias) | Unclear risk | Not specified |
| Incomplete outcome data (attrition bias) | High risk | Patients who missed four group sessions were excluded from the analyses.  Completed in treatment group n = 81 (79 %)  Completed in control group n = 87 (86 %)  lack of complete data for one-fifth of the patients, who did not complete the questionnaires at all three evaluation times |
| Selective reporting (reporting bias) | Low risk | All pre-specified outcomes reported |
| Other bias | Low risk | The trial appears to be free of other problems that could put it at a high risk of bias |
| **Espie 2008** |  |  |
| **Bias** | **Authors' judgement** | **Support for judgement** |
| Random sequence generation (selection bias) | Low risk | Centralized computer-based registration/randomization service available within the Cancer Research UK Clinical Trials Unit, Glasgow. |
| Allocation concealment (selection bias) | Unclear risk | Not specified |
| Blinding of participants and personnel (performance bias) | High risk | Due to the nature of the intervention, it was not possible to blind participants or therapists to allocation. |
| Blinding of outcome assessment (detection bias) | Unclear risk | Not specified |
| Incomplete outcome data (attrition bias) | Unclear risk | Not specified |
| Selective reporting (reporting bias) | Low risk | All outcomes pre-specified by authors reported |
| Other bias | Low risk | The trial appears to be free of other problems that could put it at a high risk of bias |
| **Ferguson 2016** |  |  |
| **Bias** | **Authors' judgement** | **Support for judgement** |
| Random sequence generation (selection bias) | Low risk | Computer randomization to treatment type (MAAT or ST) was performed for participant numbers |
| Allocation concealment (selection bias) | Unclear risk | Not specified |
| Blinding of participants and personnel (performance bias) | High risk | Computer randomization was performed and was revealed to the participant after baseline assessment |
| Blinding of outcome assessment (detection bias) | Low risk | The psychometrist responsible for all assessments remained blind to each participant’s assigned treatment condition throughout the study. |
| Incomplete outcome data (attrition bias) | Unclear risk | 7/20 participants dropped out of ST and 5/27 withdrew from MAAT. Reasons for withdrawal included an inability to commit time, personal problems (eg, family illness), or moving. The final sample for analyses was 22 participants for MAAT and 13 participants for ST. |
| Selective reporting (reporting bias) | Unclear risk | All outcomes pre-specified by authors reported |
| Other bias | Unclear risk | The trial appears to be free of other problems that could put it at a high risk of bias |
| **Fillion 2008** |  |  |
| **Bias** | **Authors' judgement** | **Support for judgement** |
| Random sequence generation (selection bias) | Low risk | The sequence of randomization was computer generated, after a preliminary stratification, according to the adjuvant treatments received. |
| Allocation concealment (selection bias) | Unclear risk | Not specified |
| Blinding of participants and personnel (performance bias) | High risk | Not possible in this study |
| Blinding of outcome assessment (detection bias) | Unclear risk | Not specified |
| Incomplete outcome data (attrition bias) | Low risk | (<20%) 3 x control group lost to follow up; 4 x experimental group lost to follow up |
| Selective reporting (reporting bias) | High risk | Pain outcomes pre-specified by authors not reported |
| Other bias | Low risk | The trial appears to be free of other problems that could put it at a high risk of bias |
| **Foster 2015** |  |  |
| **Bias** | **Authors' judgement** | **Support for judgement** |
| Random sequence generation (selection bias) | Low risk | A statistician independently generated a random allocation sequence, using ‘R’ for each NHS Centre, and participants were randomised in blocks of four [20]. |
| Allocation concealment (selection bias) | Unclear risk | Not specified |
| Blinding of participants and personnel (performance bias) | High risk | Not possible |
| Blinding of outcome assessment (detection bias) | Low risk | Statisticians and members of the research team not involved in recruitment were blinded during analysis. |
| Incomplete outcome data (attrition bias) | High risk | 36% attrition |
| Selective reporting (reporting bias) | Low risk | All outcomes pre-specified by authors reported.  Trial registered ISRCTN67521059. |
| Other bias | Low risk | The trial appears to be free of other problems that could put it at a high risk of bias |
| **Freeman 2015** |  |  |
| **Bias** | **Authors' judgement** | **Support for judgement** |
| Random sequence generation (selection bias) | Unclear risk | Assignment by adaptive randomization (minimization) was balanced by age, gender, stage, chemotherapy, surgery, radiation, and hormone use. |
| Allocation concealment (selection bias) | Unclear risk | Not specified |
| Blinding of participants and personnel (performance bias) | High risk | Not possible |
| Blinding of outcome assessment (detection bias) | Unclear risk | Not specified |
| Incomplete outcome data (attrition bias) | Unclear risk | <20% |
| Selective reporting (reporting bias) | Low risk | All outcomes pre-specified by authors reported |
| Other bias | Low risk | The trial appears to be free of other problems that could put it at a high risk of bias |
| **Gielissen 2006** |  |  |
| **Bias** | **Authors' judgement** | **Support for judgement** |
| Random sequence generation (selection bias) | Unclear risk | Random assignment was done by means of a sequence of labeled cards contained in sealed, numbered envelopes prepared by a statistical adviser. |
| Allocation concealment (selection bias) | Unclear risk | Envelopes prepared by a statistical adviser. The envelopes were opened by the researcher (M.G.) in the presence of the patient. |
| Blinding of participants and personnel (performance bias) | Unclear risk | Not possible in this study |
| Blinding of outcome assessment (detection bias) | Unclear risk | Not specified |
| Incomplete outcome data (attrition bias) | High risk | Experimental group: 9 lost to follow-up (<20%)  Control group: 12 lost to follow-up (44 out of 56… 20% = 11 people) |
| Selective reporting (reporting bias) | Low risk | All outcomes pre-specified by authors reported |
| Other bias | Low risk | The trial appears to be free of other problems that could put it at a high risk of bias |
| **Heckler 2016** |  |  |
| **Bias** | **Authors' judgement** | **Support for judgement** |
| Random sequence generation (selection bias) | Low risk | A computer-generated randomization schedule with a block size of eight, stratified by city and sex, was used to assign participants to one of four groups (from other paper: Roscoe JA, Garland SN, Heckler CE, Perlis ML, Peoples AR, Shayne M, Savard J, Daniels NP, Morrow GR (2014) Randomized placebo-controlled trial of cognitive behavioral therapy and armodafinil for insomnia after cancer treatment. J Clin Oncol. doi:  [10.1200/JCO.2014.57.6769](https://doi.org/10.1200/JCO.2014.57.6769)) |
| Allocation concealment (selection bias) | Low risk | Random assignment was conveyed to a pharmacist, who provided the study coordinator with the appropriate study medications. |
| Blinding of participants and personnel (performance bias) | Unclear risk | All study personnel and subjects were blinded regarding medication (armodafinil, placebo) assignment but not CBT-I (yes, no) condition. Random assignment was conveyed to a pharmacist, who provided the study coordinator with the appropriate study medications. |
| Blinding of outcome assessment (detection bias) | Low risk | All study personnel and subjects were blinded regarding medication (armodafinil, placebo) assignment but not CBT-I (yes, no) condition. |
| Incomplete outcome data (attrition bias) | High risk | 29 (30 %) of the 96 randomized eligible subjects did not provide post-intervention data. |
| Selective reporting (reporting bias) | Unclear risk | All outcomes pre-specified by authors reported.  Trial registered Clinicaltrials.gov NCT01091974. |
| Other bias | Unclear risk | The original grant application was approved, with modafinil 100 mg twice per day as the active medication. A switch to A 50 mg twice per day was made at the suggestion of Cephalon, which manufactured both medications and supplied the drug and matching placebo |
| **Hoffman 2012** |  |  |
| **Bias** | **Authors' judgement** | **Support for judgement** |
| Random sequence generation (selection bias) | Low risk | Random assignment was performed by operations director of the organization, who was independent from the study, by using an externally computer generated randomization program in blocks of four, which ensured allocation concealment because no clinician/researcher could anticipate or direct the allocation of participants. |
| Allocation concealment (selection bias) | Unclear risk | “No clinician/researcher could anticipate or direct the allocation of participants.” |
| Blinding of participants and personnel (performance bias) | High risk | The clinician-researcher conducting the study and delivering MBSRcould not be blinded to the allocation of participants to either the treatment or control group |
| Blinding of outcome assessment (detection bias) | Low risk | Anonymized data were collected by a research assistant who was blinded to group assignment and independent from MBSR deliver |
| Incomplete outcome data (attrition bias) | High risk | There were three instances (two patients in the intervention group and one patient in the control group) in which more than 20%of data was missing from participants at T1, and thus, according to rules set by the questionnaire manuals, their data was excluded because it was too sparse to analyze. |
| Selective reporting (reporting bias) | Low risk | All outcomes pre-specified by authors reported |
| Other bias | Low risk | The trial appears to be free of other problems that could put it at a high risk of bias |
| **Johns 2014** |  |  |
| **Bias** | **Authors' judgement** | **Support for judgement** |
| Random sequence generation (selection bias) | Low risk | The randomization sequence was generated by coin toss in blocks of four by the principal investigator. |
| Allocation concealment (selection bias) | Low risk | Research assistants and participants were blinded to the randomization sequence using sequentially numbered and sealed envelopes. |
| Blinding of participants and personnel (performance bias) | High risk | Not possible |
| Blinding of outcome assessment (detection bias) | Unclear risk | All outcomes were self-reported on study questionnaires and therefore not subject to bias by assessor interpretation. |
| Incomplete outcome data (attrition bias) | Low risk | No drop-out |
| Selective reporting (reporting bias) | Low risk | All outcomes pre-specified by authors reported.  Trial registered Clinicaltrials.gov NCT01247532 |
| Other bias | Low risk | The trial appears to be free of other problems that could put it at a high risk of bias |
| **Lengacher 2012** |  |  |
| **Bias** | **Authors' judgement** | **Support for judgement** |
| Random sequence generation (selection bias) | Unclear risk | A two-armed randomized controlled design, with randomization stratified by stage of cancer (0, I, III, and III) and treatment received (radiation treatment only or radiation treatment and chemotherapy), was used to randomly assign enrolled participants to either an MBSR(BC) group or a wait-listed control group. |
| Allocation concealment (selection bias) | Unclear risk | Not specified |
| Blinding of participants and personnel (performance bias) | Unclear risk | patients were not blinded to treatment group, |
| Blinding of outcome assessment (detection bias) | Unclear risk | Data on measures of presence of symptoms (MDASI), patient demographics, and clinical history were collected at baseline (1 week prior to the MBSR (BC) intervention) and within 2 weeks after the 6-week MBSR(BC) intervention. Participant randomization was done after baseline assessments were complete. |
| Incomplete outcome data (attrition bias) | Low risk | 1 per group loss to follow-up |
| Selective reporting (reporting bias) | Low risk | All outcomes pre-specified by authors reported |
| Other bias | Low risk | The trial appears to be free of other problems that could put it at a high risk of bias |
| **Matthews 2014** |  |  |
| **Bias** | **Authors' judgement** | **Support for judgement** |
| Random sequence generation (selection bias) | Unclear risk | Adaptive randomization program, controlling for age, insomnia severity, recruitment site, and breast cancer stage (Matthews, Cook, Terada, & Aloia, 2010). |
| Allocation concealment (selection bias) | Unclear risk | Not specified |
| Blinding of participants and personnel (performance bias) | High risk | Participants, but not the study therapist, were blind to treatment condition. |
| Blinding of outcome assessment (detection bias) | Unclear risk | Not specified |
| Incomplete outcome data (attrition bias) | Low risk | 2 loss to follow up in each group |
| Selective reporting (reporting bias) | Low risk | All outcomes pre-specified by authors reported |
| Other bias | Low risk | The trial appears to be free of other problems that could put it at a high risk of bias |
| **Prinsen 2013** |  |  |
| **Bias** | **Authors' judgement** | **Support for judgement** |
| Random sequence generation (selection bias) | Low risk | Random assignment was done by means of a sequence of labelled cards contained in sealed, numbered envelopes prepared by a statistical adviser. The envelopes were opened by the psychologists in the presence of the patient. Randomization took place per patient |
| Allocation concealment (selection bias) | Low risk | The envelopes were opened by the psychologists in the presence of the patient. Randomization took place per patient |
| Blinding of participants and personnel (performance bias) | High risk | Not possible |
| Blinding of outcome assessment (detection bias) | Unclear risk | Not reported |
| Incomplete outcome data (attrition bias) | High risk | Control: 0 loss to follow-up  Experimental: 27 lost to follow-up (>20%) |
| Selective reporting (reporting bias) | High risk | Functional impairment not in original protocol.  Trial registered Clinicaltrials.gov NCT01096641. |
| Other bias | Low risk | The trial appears to be free of other problems that could put it at a high risk of bias |
| **Reeves 2017** |  |  |
| **Bias** | **Authors' judgement** | **Support for judgement** |
| Random sequence generation (selection bias) | Low risk | The randomization sequence and group allocation were generated by a staff member not involved with the study using a computer‐generated random number sequence, with block sizes of six. |
| Allocation concealment (selection bias) | Unclear risk | Not specified |
| Blinding of participants and personnel (performance bias) | High risk | Not possible |
| Blinding of outcome assessment (detection bias) | Low risk | Data were collected by research staff, blinded to randomization assignment, at baseline and 6 months. |
| Incomplete outcome data (attrition bias) | High risk | 5/45 INTERVENTION DROPOUT  11/45 CONTROL (24%) |
| Selective reporting (reporting bias) | Low risk | All outcomes pre-specified by authors reported |
| Other bias | Unclear risk | One reported adverse event (musculoskeletal injury) was attributable to the intervention. |
| **Reich 2017** |  |  |
| **Bias** | **Authors' judgement** | **Support for judgement** |
| Random sequence generation (selection bias) | Unclear risk | Method of randomisation not clear: "were randomly assigned to either a six-week MBSR(BC) program or usual care."  Participants were randomly assigned at a one-to-one ratio to MBSR(BC) or UC with waitlisted MBSR(BC). An SPSS macro (version 17.0; SPSS, Chicago, IL) was used to create a stratified block randomization scheme,(C.A. Lengacher, R.R. Reich, C.L.Paterson, et al.Examination of broad symptom improvement due to Mindfulness-Based Stress Reduction for Breast Cancer Survivors: a randomized controlled trialJ Clin Oncol, 34 (2016), pp. 2827-2834) |
| Allocation concealment (selection bias) | Unclear risk | Method of randomisation not clear |
| Blinding of participants and personnel (performance bias) | High risk | Blinding to group assignment after the baseline assessment by the assessors was not possible with use of the waitlisted control design. (C.A. Lengacher, R.R. Reich, C.L.Paterson, et al.Examination of broad symptom improvement due to Mindfulness-Based Stress Reduction for Breast Cancer Survivors: a randomized controlled trialJ Clin Oncol, 34 (2016), pp. 2827-2834) |
| Blinding of outcome assessment (detection bias) | Unclear risk | not clear |
| Incomplete outcome data (attrition bias) | Low risk | 152/167 (92%) participants in the intervention group and 147/155 (94%) in the usual care group completed |
| Selective reporting (reporting bias) | Unclear risk | All outcomes pre-specified by authors reported.  Trial registered Clinicaltrials.gov NCT01177124. |
| Other bias | Unclear risk | The trial appears to be free of other problems that could put it at a high risk of bias |
| **Reif 2012** |  |  |
| **Bias** | **Authors' judgement** | **Support for judgement** |
| Random sequence generation (selection bias) | Low risk | Computer-generated randomisation lists were used for concealed allocation by central telephone calls. |
| Allocation concealment (selection bias) | Low risk | concealed allocation by central telephone calls. |
| Blinding of participants and personnel (performance bias) | High risk | Patients and tutors could not be blinded to treatment allocation for practical reasons. |
| Blinding of outcome assessment (detection bias) | Low risk | data entry and analysis was performed by blinded researchers |
| Incomplete outcome data (attrition bias) | Low risk | 6% loss to intervention, 15% loss to control |
| Selective reporting (reporting bias) | Low risk | All outcomes pre-specified by authors reported.  Trial registered Clinicaltrials.gov NCT00552552 |
| Other bias | Low risk | The trial appears to be free of other problems that could put it at a high risk of bias |
| **Ritterband 2012** |  |  |
| **Bias** | **Authors' judgement** | **Support for judgement** |
| Random sequence generation (selection bias) | Unclear risk | Random group assignment was based on a computer-generated randomization schedule managed by the project coordinator |
| Allocation concealment (selection bias) | Unclear risk | Not specified |
| Blinding of participants and personnel (performance bias) | High risk | participants received an email with notification of their assignment to either the experimental (Internet) or waitlist control group. |
| Blinding of outcome assessment (detection bias) | Unclear risk | Not specified |
| Incomplete outcome data (attrition bias) | Low risk | No dropout |
| Selective reporting (reporting bias) | Low risk | All outcomes pre-specified by authors reported |
| Other bias | Unclear risk | Drs. Ritterband and Thorndike are equity holders of BeHealth Solutions, Inc, which is negotiating a license for the software described in this paper. |
| **Rogers 2017** |  |  |
| **Bias** | **Authors' judgement** | **Support for judgement** |
| Random sequence generation (selection bias) | Low risk | randomization was based on computer‐generated numbers in blocks of 4 within each recruiting site. |
| Allocation concealment (selection bias) | Low risk | Randomization occurred in the order in which the participants completed baseline testing with study staff being unaware of the randomization until the moment the randomization result was revealed by opening an opaque sealed envelope. |
| Blinding of participants and personnel (performance bias) | High risk | participant blinding to study group was not possible, |
| Blinding of outcome assessment (detection bias) | Low risk | data entry and management were performed by individuals blinded to the participant's group allocation |
| Incomplete outcome data (attrition bias) | Low risk | 222 participants completed baseline testing and were randomized (110 to BEAT Cancer and 112 to usual care). Retention was similar in both groups (97% at M3 and 96% at M6). |
| Selective reporting (reporting bias) | Unclear risk | All outcomes pre-specified by authors reported.  Trial registered Clinicaltrials.gov NCT00929617  Original protocol (written in 2009) proposed assessing depression and anxiety as sources of physical activity self-efficacy [2]. In this paper these are reported as health outcomes because “updated literature reviews indicate a significant burden of suffering caused by psychosocial symptoms and a clear knowledge gap regarding the ability of behavior change interventions to translate exercise training benefits.” |
| Other bias | Unclear risk | Only one related serious adverse event occurred (intervention group; pelvic stress fracture). Related expected adverse events in the BEAT Cancer group included back or lower extremity musculoskeletal pain or injury (n = 14), heart rate monitor rash (n = 1), fall while walking (n = 1), breast reconstruction (n = 3), and chest pain during treadmill fitness test (n = 1). Related adverse events in the UC group included arm tingling (n = 1) during the treadmill test and knee tendonitis (n = 1) [3]. |
| **Sandler 2017** |  |  |
| **Bias** | **Authors' judgement** | **Support for judgement** |
| Random sequence generation (selection bias) | Low risk | randomly allocated (computer-generated sequence) |
| Allocation concealment (selection bias) | Unclear risk | Allocation was concealed from the coordinator until intervention commencement. |
| Blinding of participants and personnel (performance bias) | High risk | Personnel, were not blind to allocated interventions |
| Blinding of outcome assessment (detection bias) | Unclear risk | Insufficient nformation to judge of risk on blinding of outcome assessments. An independent psychologist who was blinded to group allocation conducted the Semistructured Clinical Interview for Neurasthenia (SCIN) |
| Incomplete outcome data (attrition bias) | Low risk | 2/24 control and 3/22 intervention. A total of five participants discontinued because of work or family commitments. All participants completed baseline self-report questionnaires allowing an intention-to-treat analysis to be conducted. |
| Selective reporting (reporting bias) | Unclear risk | All outcomes pre-specified by authors reported.  Trial registered Australian New Zealand Clinical Trials Registry ACTRN12611000338965 |
| Other bias | High | Only provided 70% of the original statistical power estimate, and Type II error is therefore plausible.  The protocol steering committee provided a waiver for one participant who had completed adjuvant therapy 17 months before being screened. |
| **Savard 2005** |  |  |
| **Bias** | **Authors' judgement** | **Support for judgement** |
| Random sequence generation (selection bias) | Unclear risk | Not clear: "randomly assigned" |
| Allocation concealment (selection bias) | Unclear risk | Not specified |
| Blinding of participants and personnel (performance bias) | High risk | Not possible |
| Blinding of outcome assessment (detection bias) | Unclear risk | Not specified |
| Incomplete outcome data (attrition bias) | Low risk | <20% |
| Selective reporting (reporting bias) | Low risk | All outcomes pre-specified by authors reported |
| Other bias | Low risk | The trial appears to be free of other problems that could put it at a high risk of bias |
| **Van Der Lee 2012** |  |  |
| **Bias** | **Authors' judgement** | **Support for judgement** |
| Random sequence generation (selection bias) | Low risk | First, the researcher used SPSS syntax to randomly select 12 participants out of all eligible candidates in file at that moment. |
| Allocation concealment (selection bias) | High risk | Not concealed |
| Blinding of participants and personnel (performance bias) | High risk | Not possible |
| Blinding of outcome assessment (detection bias) | Unclear risk | Not reported |
| Incomplete outcome data (attrition bias) | Low risk | (<20%) Intervention: 82% completed T2 questionaire; Control: 97% completed T2 questionaire |
| Selective reporting (reporting bias) | High risk | HADs means not reported |
| **van Weert 2010** |  |  |
| **Bias** | **Authors' judgement** | **Support for judgement** |
| Random sequence generation (selection bias) | Low risk | Randomization was conducted at the group level by an independent researcher using a randomization list. |
| Allocation concealment (selection bias) | Unclear risk | Not specified |
| Blinding of participants and personnel (performance bias) | High risk | Therapists could not be blinded, as they had to schedule the intervention sessions. Until the first session, participants were blinded to the intervention they were allocated to receive |
| Blinding of outcome assessment (detection bias) | High risk | Main investigators were not blinded to group assignment |
| Incomplete outcome data (attrition bias) | Low risk | <20% for all groups |
| Selective reporting (reporting bias) | Low risk | All pre-specified outcomes reported. |
| Other bias | Low risk | The trial appears to be free of other problems that could put it at a high risk of bias |
| **Willems 2016** |  |  |
| **Bias** | **Authors' judgement** | **Support for judgement** |
| Random sequence generation (selection bias) | Low risk | the computer randomly assigned |
| Allocation concealment (selection bias) | Unclear risk | Fully automated |
| Blinding of participants and personnel (performance bias) | High risk | Not possible |
| Blinding of outcome assessment (detection bias) | Low risk | Fully automated |
| Incomplete outcome data (attrition bias) | Low risk | <20% |
| Selective reporting (reporting bias) | Low risk | All prespecified outcomes reported.  Trial registered Dutch Trial Register (NTR3375) |
| Other bias | Unclear risk | Change to protocol: Originally, this criterion was set to 6-52 weeks [13]. After recruitment, we noted that participants were included outside this criterion. The lower limit of 6 weeks was set so participants had had a sufficient recovery period after treatment before participating in the study; the upper limit of one year was set to include participants highest in their distress. Since participants voluntarily participated and can decide whether they are able to participate and levels of distress are still high 56 weeks after treatment [22], we adjusted this criterion to 4-56 weeks. This led to an additional 13 participants in the control condition and 7 in the intervention condition. |
| **Yun 2017** |  |  |
| **Bias** | **Authors' judgement** | **Support for judgement** |
| Random sequence generation (selection bias) | Low risk | With the aid of a computerized random number generator (SAS 9.1.3, Proc plan), we randomly assigned eligible participants, two-to-one, to the intervention or the usual care group. To minimize the effects of potentially confounding variables on outcomes, we performed block randomization with 8 strata defined by type of cancer (breast, stomach, colon, or lung) and number of behavior goals practiced at the study entry (0 or 1 out of 3 defined possible behaviors). |
| Allocation concealment (selection bias) | Unclear risk | not clear |
| Blinding of participants and personnel (performance bias) | High risk | Masking:None (Open Label) |
| Blinding of outcome assessment (detection bias) | Unclear risk | not clear |
| Incomplete outcome data (attrition bias) | High risk | In the LP group, 115 (69.3%) participants completed the 12-month course at 3 months and 117 (70.5%) at 6–12 months. In the UC group, 60 (73.2%) participants completed the course at 3 months and 57 (71.3%) at 12 months. |
| Selective reporting (reporting bias) | Low risk | All outcomes prespecified by author reported  Trial registered Clinicaltrials.gov NCT01527409 |
| Other bias | Unclear risk | The trial appears to be free of other problems that could put it at a high risk of bias |
| **Yun 2012** |  |  |
| **Bias** | **Authors' judgement** | **Support for judgement** |
| Random sequence generation (selection bias) | Low risk | An independent statistician generated a randomization table with NQuery Advisor 6.01 (Statistical Solutions, Saugus, MA) and used the table to assign each patient to either the intervention group or the usual care group. |
| Allocation concealment (selection bias) | Unclear risk | Independent statistician used the table to assign each patient to either the intervention group or the usual care group. |
| Blinding of participants and personnel (performance bias) | High risk | Not possible |
| Blinding of outcome assessment (detection bias) | Low risk | An independent research coordinator (nurse) managed both groups |
| Incomplete outcome data (attrition bias) | Low risk | 23 of 136 loss to follow-up on intervention arm |
| Selective reporting (reporting bias) | Low risk | All outcomes prespecified byy authors reported.  Trial registered Clinicaltrials.gov NCT01228773 |
| Other bias | Low risk | The trial appears to be free of other problems that could put it at a high risk of bias |
