# Supplementary material for: The effectiveness of psychological interventions for fatigue in cancer survivors: systematic review of randomised controlled trials
Source: Syst Rev. 2019 Dec 13;8:324. doi: 10.1186/s13643-019-1230-2 (PMC6911282; doi:10.1186/s13643-019-1230-2)
Supplement: Supplementary file 3 — Additional file 3. Search strategies used in this review. [file 13643_2019_1230_MOESM3_ESM.docx]

Additional file 3 Search strategies used in this review

|  | | **Ovid MEDLINE(R); Embase; CancerLit** |
| --- | --- | --- |
|  | | **Search Terms** |
| S5 | | S1 AND S2 AND S3 AND S4 |
| S4 | | (randomized controlled trial or controlled clinical trial or 'random assignment').mp. [mp=ti, ab, hw, tn, ot, dm, mf, dv, kw, fx, nm, kf, px, rx, an, ui, sy] |
| S3 | | (fatigue or asthenia or asthenic or asthenia or (exhaustion or exhausted) or 'loss of energy' or 'loss of vitality' or (weary or weariness or weakness) or (apathy or apathetic or lassitude or lethargic or lethargy) or (sleepy or sleepiness or drowsy or drowsiness) or (tired or tiredness)).mp. [mp=ti, ab, hw, tn, ot, dm, mf, dv, kw, fx, nm, kf, px, rx, an, ui, sy] |
| S2 | | (Psych* or Behav* or Therap*or hypnosis or relaxation or imagery or cogniti*).mp. [mp=ti, ab, hw, tn, ot, dm, mf, dv, kw, fx, nm, kf, px, rx, an, ui, sy] |
| S1 | | ('cancer survivors' or neoplasm or survivor or cancer or remission).mp. [mp=ti, ab, hw, tn, ot, dm, mf, dv, kw, fx, nm, kf, px, rx, an, ui, sy] |
|  | **EBSCOhost Research Databases**  **CINAHL Plus with Full Text; Psychinfo** | |
|  | **Search Terms** | |
| S5 | S1 AND S2 AND S3 AND S4 | |
| S4 | (randomized controlled trial or controlled clinical trial or “random assignment”) | |
| S3 | (fatigue or asthenia or asthenic or asthenia or (exhaustion or exhausted) or “loss of energy” or “loss of vitality” or (weary or weariness or weakness) or (apathy or apathetic or lassitude or lethargic or lethargy) or (sleepy or sleepiness or drowsy or drowsiness) or (tired or tiredness)) | |
| S2 | Behav* OR Therap* or hypnosis or relaxation or imagery or cognition or psych* or cognit* | |
| S1 | 'cancer survivors' or neoplasm or survivor or cancer or remission | |

|  | **Web of Science** |
| --- | --- |
|  | **Search Terms** |
| S5 | #4 AND #3 AND #2 AND #1 |
| S4 | **TOPIC:** ((randomized controlled trial or controlled clinical trial or “random assignment”)) |
| S3 | **TOPIC:** ((fatigue or asthenia or asthenic or asthenia or (exhaustion or exhausted) or “loss of energy” or “loss of vitality” or (weary or weariness or weakness) or (apathy or apathetic or lassitude or lethargic or lethargy) or (sleepy or sleepiness or drowsy or drowsiness) or (tired or tiredness))) |
| S2 | **TOPIC:** (Behavi* or Therap* or hypnosis or relaxation or imagery or psych* or cognit*) |
| S1 | **TOPIC:** ('cancer survivors' or neoplasm or survivor or cancer or remission) |
